# “Precision on Two Wheels”—Structural Refinement of ^64^Cu- and ^68^Ga-Labeled Bicyclic Peptides Targeting Nectin‑4 for Improved Tumor Imaging: From Preclinical Development to First-in-Human Application

**DOI:** 10.1021/acs.jmedchem.5c02371

**Published:** 2025-10-13

**Authors:** Tobias Krönke, Johanna Trommer, Martin Ullrich, Markus Laube, Reik Löser, Jérôme Kretzschmar, Marie Urbanova, Sven Stadlbauer, Florian Brandt, Ivan Platzek, Sebastian Hoberück, Jörg Kotzerke, Christian Thomas, Matthias Miederer, Ralph A. Bundschuh, Klaus Kopka, Jens Pietzsch, Robert Wodtke

**Affiliations:** † Institute of Radiopharmaceutical Cancer Research, 28414 Helmholtz-Zentrum Dresden-Rossendorf, Bautzner Landstraße 400, 01328 Dresden, Germany; ‡ Institute of Resource Ecology, 28414 Helmholtz-Zentrum Dresden-Rossendorf, Bautzner Landstraße 400, 01328 Dresden, Germany; § Department of Physics and Measurements, University of Chemistry and Technology, Technická 5, Prague 16628, Czech Republic; ∥ Klinik und Poliklinik für Nuklearmedizin, University Hospital Carl Gustav Carus at the Technische Universität Dresden, Fetscherstraße 74, 01307 Dresden, Germany; ⊥ Klinik und Poliklinik für Urologie, University Hospital Carl Gustav Carus at the Technische Universität Dresden, Fetscherstraße 74, 01307 Dresden, Germany; # School of Science, Faculty of Chemistry and Food Chemistry, Technische Universität Dresden, Mommsenstraße 4, 01069 Dresden, Germany; ∇ National Center for Tumor Diseases (NCT), NCT/UCC Dresden, a Partnership between DKFZ, Faculty of Medicine and University Hospital Carl Gustav Carus, TUD Dresden University of Technology & Helmholtz-Zentrum Dresden-Rossendorf (HZDR), 01307 Dresden, Germany; ○ German Cancer Consortium (DKTK), Partner Site Dresden, 01307 Dresden, Germany; ◆ German Cancer Research Center (DKFZ), 69120 Heidelberg, Germany

## Abstract

The cell adhesion protein nectin-4 emerged as a valid therapeutic target for antibody- and peptide-drug conjugates in cancer. To support patient stratification for such targeted therapies, there is a clinical need for molecular imaging agents capable of quantifying nectin-4 levels noninvasively in vivo. For this purpose, we developed ^64^Cu- and ^68^Ga-labeled ligands derived from bicyclic peptide-drug conjugate **BT8009**. A library of peptides was prepared with a major focus on the bioisosteric replacement of the original methionine residue due to its susceptibility to oxidation. The peptides were characterized for their binding behavior to nectin-4, and radiopharmacological characterization of selected radioligands was performed using urothelial carcinoma cell lines and tumor xenograft models derived thereof. The suitability of the most promising ligand from the preclinical studies, **NECT-224**, for PET imaging purposes was also demonstrated in a first-in-human application using **[** ^**68**^ **Ga]­Ga-NECT-224**. The results suggest its further clinical development, but also that of **[** ^**64**^ **Cu]­Cu-NECT-224**.

## Graphical abstract

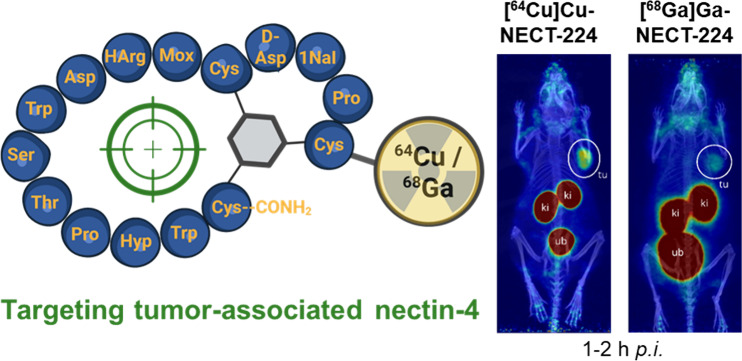

## Introduction

For several years now, the field of radiopharmacy and, in particular, the development of novel targeted radiopharmaceuticals for the radionuclide theranostics of tumor diseases has witnessed a significant increase in interest and growth. This is mainly attributed to the successful clinical translation of both diagnostic and therapeutic radioligands targeting the somatostatin receptor subtype 2 (SST_2_) to treat neuroendocrine tumors and prostate-specific membrane antigen (PSMA) to treat prostate cancer. Motivated by these prime examples, there is an ongoing search to expand the radionuclide theranostic opportunities for cancer patients suffering also from other organ-specific tumor entities. Accordingly, besides further optimizing existing radioligands, current radiopharmaceutical developments encompass the identification of novel molecular targets, the exploration of appropriate targeting molecules that can be radiolabeled, and the application of novel radionuclides along with strategies for their introduction into these molecules.^1–3^

Regarding the nature of the targeting molecule, the broad range of vectors from small molecules, peptidomimetics, and peptides up to antibodies and analogs thereof is pursued. Although linear and especially monocyclic peptides are frequently used for radioligands, there are only a few examples reported for radiolabeled bicyclic peptides so far.^4,5^ In recent years, bicyclic peptides, in which three cysteine residues are attached through their thiol functionality to a central organic scaffold, have gained increasing attraction as drug modalities. This is either based solely on their mode of action (e.g., enzyme inhibitors) or on their use as vector molecules for attaching payloads such as cytotoxic agents, fluorophores, or radionuclides.^6–9^ While the extraordinary bioactive potential of bicyclic peptides was recognized around the middle of the past century^10^ and peptides with triple cysteine-bridges were already reported more than 40 years ago,^11,12^ an important step forward for their use as targeting molecules was provided by Heinis et al.^13^ who developed a phage display-based approach for their target-directed identification. In addition to a highly specific and affine target binding, a (bi)­cyclic scaffold generally promises a good metabolic stability, i.e., stability toward proteolytic degradation, and a rapid clearance from the organism via the kidneys,^14^ which comply with the common requirements for theranostic radioligands. Moreover, the moderate size of bicyclic peptides enables their structural optimization by chemical synthesis to further improve metabolic stability and binding affinity.^14–17^

Eder et al.^16^ reported the first translation of a bicyclic peptide hit from phage screening toward the membrane type 1 matrix metalloproteinase MT1-MMP (or MMP-14) into radioconjugates for imaging and therapeutic purposes, including the introduction of non-natural amino acids to improve the proteolytic stability and the attachment of a fatty acid to modify the blood circulation time. Subsequently, a series of other studies aimed at developing bicyclic radioconjugates toward the erythropoietin-producing hepatocellular receptor A2 (EphA2) for imaging purposes.^18–20^

Considering the promising results for radiolabeled bicyclic peptides as imaging probes, the broadening to other molecular targets is obvious, which might also advance our understanding of how to further optimize these molecules for radiotheranostic applications in general. In addition to MMP-14 and EphA2, bicyclic peptides were mainly identified for enzymes,^15,21^ but recently also for more challenging target proteins such as thymic stromal lymphopoietin^22^ and nectin-4.^17^ The latter protein is particularly interesting for the development of targeted radioligands due to its high abundance in various tumor entities but low to moderate abundance in healthy tissues, with the exception of embryonic and placental tissues.^23–26^ Apart from nectin-4, three further family members (nectin-1, -2, and -3) have been identified, which are all type I transmembrane polypeptides and act primarily as Ca^2+^-independent cell adhesion proteins. They exhibit a cytoplasmic tail, a transmembrane region, and three immunoglobulin-like domains (Ig-like V/D_1_/D_2_) in their ectodomain. Dimerization of two nectin molecules on the same plasma membrane (*cis*-dimers) can occur, followed by the formation of *trans*-homo/heterodimers with *cis-*dimers on opposing cells, which represents the basis for their function as cell adhesion proteins.^27^

With the antibody drug conjugate **enfortumab vedotin** (**Padcev**), a nectin-4-directed therapy has been clinically approved for patients with locally advanced or metastatic urothelial carcinoma who have already received immunotherapy (targeting PD-1 or PD-L1) and a platinum-based chemotherapy.^28^ In the meantime, **enfortumab vedotin** in combination with PD-1 targeting **pembrolizumab** has been approved as a first-line treatment.^29,30^ As an alternative targeted therapy toward nectin-4, Mudd et al.^17^ reported the discovery of **BT8009** (**zelenectide vedotin**, Figure 1), a bicyclic nectin-4 targeting peptide (**BCY8126**, Figure 1) that uses TATA (1,3,5-triacryloyl-1,3,5-triazinane) as cross-linking unit and the same cleavable linker (maleimidocaproyl-valine-citrulline-*p*-aminobenzyloxycarbonyl) and cytotoxic agent (MMAE, monomethyl auristatin E) as **enfortumab vedotin**, but harbors an additional sarcosin_10_ spacer between the bicyclic peptide and the cleavable linker. **BT8009** showed significant antitumor activity in preclinical tumor models, which was even superior, or at least comparable to, the activity of an analog of **enfortumab vedotin**.^31^ **BT8009** is currently investigated in clinical trials for the treatment of locally advanced or metastatic urothelial (NCT06225596) and breast cancer (NCT06840483).

**Figure 1:**
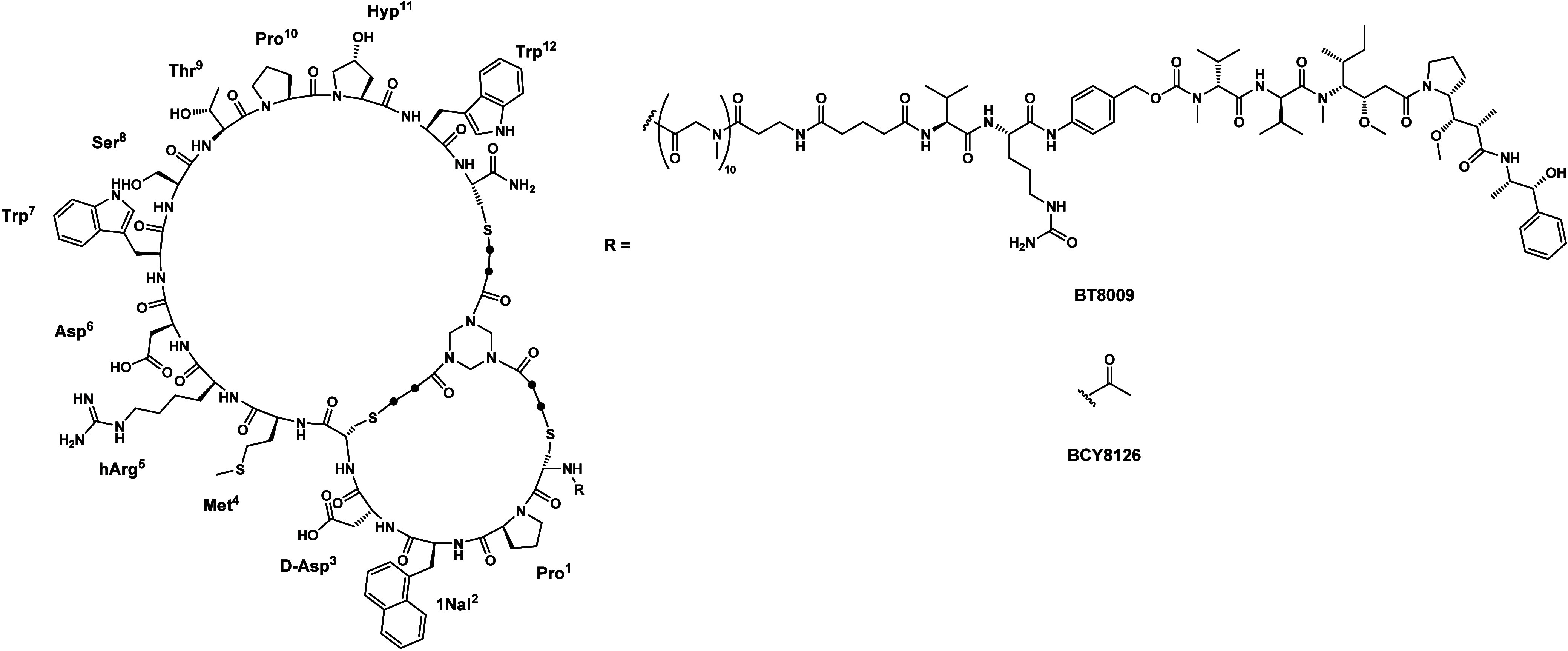
Structures of BT8009 and BCY8126, which served as the basis for the radioligand design herein. For a better overview, the CH_2_ groups of the cross-linking unit are depicted as black dots.

Although a first targeted therapy toward nectin-4 is approved (**enfortumab vedotin**) and a second one might likely receive approval (**BT8009**), there is a clinical need to assess the nectin-4 status in patients prior to these therapies, as not all patients will benefit from such therapies.^32^ In this context, for administration of **enfortumab vedotin** to patients with urothelial carcinoma the nectin-4 status does not need to be determined beforehand, which was judged from previous clinical trials showing that most of these patients exhibit a high level of nectin-4.^33^ However, Klümper et al.^33^ recently discovered that the abundance of nectin-4 is often strongly decreased or even absent in metastases compared to the primary tumor. Furthermore, a low or absent nectin-4 level was associated with less efficient therapy with **enfortumab vedotin**. The proportion of nectin-4-positive patients might be lower for other cancer types, and the changes in the protein level upon metastatic spread are not yet explored.

The clinical demand for assessing the nectin-4 status in patients prompted us to translate **BT8009** into a bicyclic radioligand that might possess more favorable properties for imaging purposes compared to reported nectin-4-directed immunoPET and immunoSPECT approaches.^34–37^ Herein, we describe the synthesis and radiopharmacological characterization of bicyclic peptides derived from **BT8009**, in which we omitted MMAE, the cleavable linker, and the sarcosin_10_ spacer but introduced suitable chelating units for labeling with ^64^Cu and ^68^Ga. From a radiochemical perspective, we noted that the methionine residue in position 4 of the bicyclic peptide is generally amenable to an undesired oxidation during peptide synthesis and/or subsequent radiolabeling and, therefore, decided to focus on bioisosteric replacements to improve the synthesis yield and radiochemical purity. Furthermore, during the course of our work, Duan et al.^38^ were the first to report on a radiolabeled bicyclic peptide targeting nectin-4 ([^**68**^ **Ga]­Ga–N188**, denoted as ^**68**^ **Ga–N188** in the original publication), including preclinical and clinical results for imaging of urothelial carcinoma, which was later also expanded to other tumor entities.^39^ The design of [^**68**^ **Ga]­Ga–N188** corresponds to our approach; however, compared to the parent nectin-4-targeting peptide **BCY8126**, the original homoarginine in position 5 and 1-naphthylalanine in position 2 were substituted by arginine and 2-naphthylalanine, respectively. Furthermore, [^**68**^ **Ga]­Ga–N188** contains a free C-terminus compared to the C-terminal primary amide functionality of **BCY8126**. To shed light on the influence of the amino acid substitution and the kind of C-terminus, **N188** and corresponding analogs were synthesized. We characterized all peptides regarding their binding affinity to nectin-4 in more detail by using a fluorescence anisotropy-based competitive binding assay and surface plasmon resonance (SPR) spectroscopy. Selected ^64^Cu- and ^68^Ga-labeled ligands were then radiopharmacologically characterized with a focus on chemical and metabolic stability and nectin-4-specific binding on intact cells using human urothelial cancer cell lines. A series of radioligands was then evaluated in vivo for targeting tumor-associated nectin-4 by small animal PET/CT imaging. Of all studied radioligands, **[** ^**64**^ **Cu]­Cu-4**, also named **[** ^**64**^ **Cu]­Cu-NECT-224,** turned out to exhibit the best performance in terms of tumor uptake and tumor-to-tissue ratios. Further, **[** ^**68**^ **Ga]­Ga-NECT-224** was progressed to a first-in-human application.

## Results and Discussion

### Synthesis of the Bicyclic Peptides and Characterization of Their Binding Affinity to Recombinant Human Nectin-4

The parent linear amino acid sequence of the Bicyclic scaffold (**BCY8126**) derived from **BT8009** is as follows: Cys-Pro^1^-1NaI^2^-d-Asp^3^-Cys-Met^4^-hArg^5^-Asp^6^-Trp^7^-Ser^8^-Thr^9^-Pro^10^-Hyp^11^-Trp^12^-Cys-CONH_2_. This 15mer linear peptide, as well as its analogs, was assembled onto the Rink-Amide resin using an automated microwave peptide synthesizer (Biotage Initiator+ Alstra) with standard conditions for Fmoc removal (20% piperidin/DMF) and amino acid coupling (HATU/DIPEA in DMF). Subsequently, the N-terminal groups, i.e., acetyl, chelating units, and fluorophores, were manually coupled, and TFA-mediated cleavage from the resin provided the unprotected linear peptides. For the peptides harboring methionine, *S*-ethylcysteine, or selenomethionine in position 4, partial oxidation to the respective sulfoxides and selenoxides was observed. Cyclization to the bicyclic scaffold (3 × 9 format) with TATA via Thia-Michael addition of the three thiol groups was performed under aqueous-basic conditions as previously described for **BT8009** (Scheme S1).^17^ For compound **8d** (**N188**), Fmoc-l-Cys­(Trt)–OH was manually loaded onto the 2-ClTrtCl resin, and subsequent steps were performed as done for the peptides with C-terminal amide functionality. Purification of all peptides was done by RP-HPLC, which afforded the final bicyclic peptides in overall yields ranging between 4 and 13% (based on the initial resin loading) and in good chemical purities (>95%, Table 1) with the exception of bicyclic peptide **5**, which bears selenomethionine in position 4. Immediately after purification, the respective selenoxide emerged, which resulted in low chemical purity (61%). It is worth noting that the use of PEG-based Rink amide resin (ChemMatrix) compared to polystyrol-based resin did not improve the synthesis yield. To investigate the influence of the methionine sulfoxide on the binding to nectin-4, the authentic compound (as a mixture of diastereomers) was synthesized by incubation of peptide **1a** in a H_2_O_2_ solution (100 mM).^40^ Figure 2 provides an overview of the different structural modifications applied to the original bicyclic scaffold, including the introduced N-terminal modifications.

**Figure 2:**
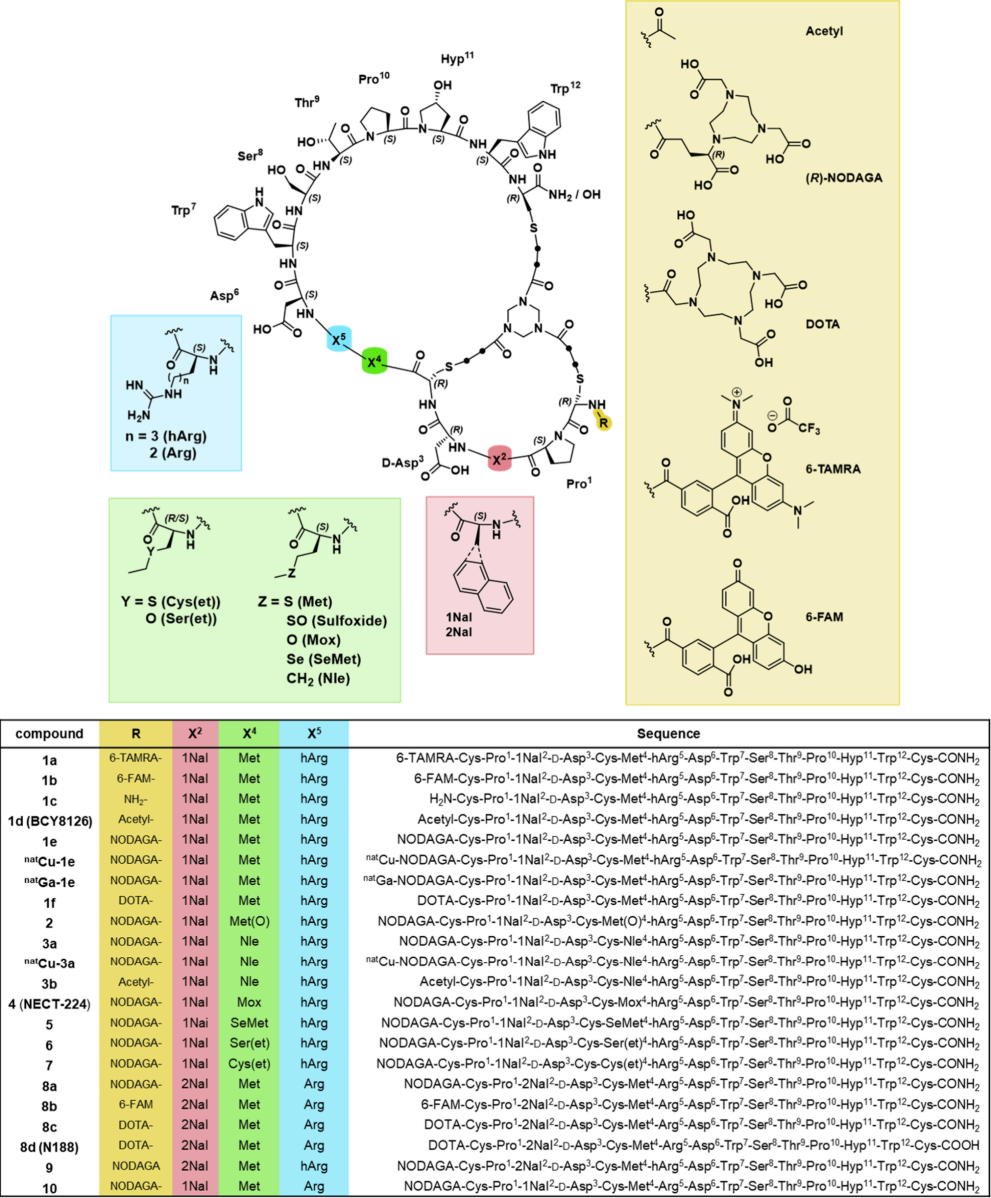
Overview of the structures of the bicyclic peptides studied herein. For a better overview, the CH_2_ groups of the cross-linking unit are depicted as black dots.

**Table 1:** Analytical Data of the Bicyclic Peptides

compound	chemical formula	*m*/*z* calculated for [M+2H]^2+^	*m*/*z* found^a^ for [M+2H]^2+^	purity (%)^b^
**1a**	C_123_H_151_N_26_O_29_S_4_	1293.0084	1293.0058	>98
**1b**	C_119_H_140_N_24_O_31_S_4_	1265.4572	1265.4556	>98
**1c**	C_98_H_130_N_24_O_25_S_4_	1086.4334	1086.4333	>98
**1d (BCY8126)**	C_100_H_132_N_24_O_26_S_4_	1107.4387	1107.4377	>97
**1e**	C_113_H_153_N_27_O_32_S_4_	1265.0102	1265.0091	>95
^**nat**^ **Cu-1e**	C_113_H_151_CuN_27_O_32_S_4_	1295.4671	1295.4665	>95
^**nat**^ **Ga-1e**	C_113_H_151_GaN_27_O_32_S_4_	1298.4629	1298.4632	>96
**1f**	C_114_H_156_N_28_O_32_S_4_	1279.5234	1279.5251	>99
**2**	C_113_H_153_N_27_O_33_S_4_	1273.0076	1273.0090	>95
**3a**	C_114_H_155_N_27_O_32_S_3_	1256.0320	1256.0310	>99
^**nat**^ **Cu-3a**	C_114_H_153_CuN_27_O_32_S_3_	1286.4889	1286.4868	>98
**3b**	C_101_H_134_N_24_O_26_S_3_	1098.4604	1098.4588	>97
**4 (NECT-224)**	C_113_H_153_N_27_O_33_S_3_	1257.0216	1257.0205	>98
**5**	C_113_H_153_N_27_O_32_S_3_Se	1288.9824	1288.9850	>61
**6**	C_113_H_153_N_27_O_33_S_3_	1257.0216	1257.0223	>98
**7**	C_113_H_153_N_27_O_32_S_4_	1265.0102	1265.0103	>97
**8a**	C_112_H_151_N_27_O_32_S_4_	1258.0023	1258.0018	>95
**8b**	C_118_H_138_N_24_O_31_S_4_	1258.4494	1258.4465	>99
**8c**	C_113_H_154_N_28_O_32_S_4_	1272.5156	1272.5147	>99
**8d (N188)**	C_113_H_153_N_27_O_33_S_4_	1273.0076	1273.0062	>96
**9**	C_112_H_151_N_27_O_32_S_4_	1258.0023	1258.0027	>97
**10**	C_113_H_153_N_27_O_32_S_4_	1265.0102	1265.0095	>97

a High-resolution mass spectra using electrospray ionization were recorded.

b Purity was determined by analytical RP-HPLC and is given for 230 nm.

For characterizing the binding affinity of the bicyclic peptides toward nectin-4, we envisaged a fluorescence anisotropy-based competitive binding assay. Mudd et al. also used such an assay for the development of the bicycle drug conjugate **BT8009**.^17^ Herein, the peptidic scaffold of **BT8009** was used as a basis for the required fluorescent nectin-4-ligand (probe) with either 6-TAMRA (**1a**) or 6-FAM (**1b**) being attached at the N-terminal Cys residue. The binding affinities of these two probes were assessed by measuring the changes in fluorescence anisotropy (FA) over a range of recombinant human nectin-4 concentrations at a constant concentration of the respective probe (1 nM; Figure 3A). In this context, the binding of the probe to nectin-4 was started by the addition of nectin-4, followed by continuous fluorescence measurements over 20 min. It is worth noting that a slight initial increase in the FA values could be observed for both probes (FA values at <300 s in Figure S1); however, this putative time-dependent association appeared to be too fast to analyze it. For the 6-TAMRA probe **1a**, a dissociation constant (*K*_d_) of 0.94 (±0.09) nM and a dynamic range (ΔFA = mA of bound probe – mA of free probe)^41^ of 100 (±3) mA was derived, while for the 6-FAM probe **1b** a *K*_d_ value of 0.17 (±0.01) nM and a dynamic range of 86 (±7) mA was obtained. Considering the fact that the probe concentration should be in the range of or lower than 2 × *K*_d_ to avoid a stoichiometric titration,^42^ the obtained *K*_d_ value of **1b** was determined with just sufficient accuracy under the applied conditions ([**1b**] ≈ 6 × *K*_d_). For the competitive binding assay, we decided to use the 6-TAMRA probe **1a** due to the slightly greater dynamic range as well as a more favorable synthetic access (fewer synthesis steps and higher yield compared to **1b**).

**Figure 3:**
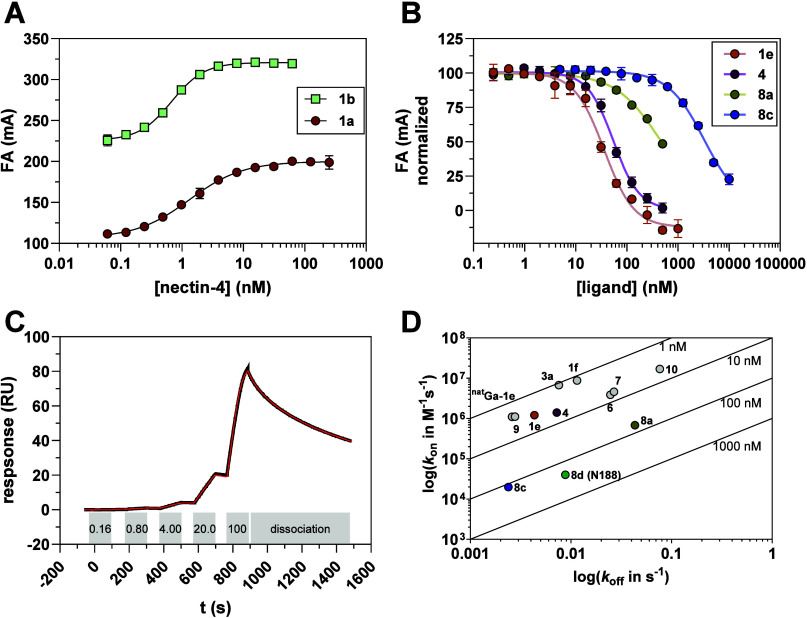
Binding of peptides to recombinant human nectin-4. (A) Binding curves of fluorescent probes **1a** and **1b** to human nectin-4 as determined by FA change. Conditions: 1 nM **1a** and **1b**, 0.06–250 nM (for **1a**) and 0.06–62.5 nM (for **1b**) recombinant human nectin-4, HEPES buffer (20 mM, pH 7.4, 0.01% Tween20, 150 mM NaCl, 1% DMSO). Data shown are mean values (±SD) of three experiments, each performed in duplicate. (B) Competitive binding curves of selected nonfluorescent peptides using probe **1a**. Data shown are mean values (±SD) of two experiments, each performed in duplicate. Conditions: 1 nM **1a**, 20 nM nectin-4, 0.24–1000 nM **1e**, 0.24–500 nM **4**, 0.24–500 nM **8a**, 4.88–10,000 nM **8c**, same buffer as in (A). (C) Exemplary SPR sensorgram for single-cycle-kinetic analysis of **4** (red) as analyte to immobilized human nectin-4, as well as the fit (black) as obtained according to a 1:1 binding model. The concentrations (in nM) and intervals of additions of compound **4**, as well as the dissociation phase, are indicated below the sensorgram. Conditions: HBS-P+ buffer (pH 7.4). (D) Plot of log­(*k*_off_) vs log­(*k*_on_) for the rate constants derived from SPR experiments. The diagonal lines reflect dissociation constants of 1, 10, 100, and 1.000 nM. For peptides **1e**, **4**, **8a**, and **8c**, the same color coding as in B was used.

For the competitive binding assay, a constant concentration of probe **1a** (1 nM) and of nectin-4 (20 nM) was used, and the concentration of the competitors was varied over at least 3 orders of magnitude. Competitive binding curves for selected nonfluorescent peptides are shown in Figure 3B (see Figure S2 for the curves of all other peptides). Analysis of the curves by nonlinear regression according to dose–response inhibition with variable slope, as implemented in GraphPad Prism (see Experimental section), provided the IC_50_ value for displacing the probe from nectin-4. The IC_50_ values were then transformed into *K*_i_ or *K*_d_ values (we decided to use *K*_d_ instead of *K*_i_ due to the 1:1 binding) according to the mathematical equation derived by Nikolovska-Coleska et al.^41^ This approach is superior for transforming the IC_50_ values compared to the classic Cheng–Prusoff equation (*K*_i_ = IC_50_/(1 + [*L*]/*K*_d_) as it considers the special characteristics of FA-binding assays and provides thus more reasonable data.^41^

All nonfluorescent ligands were characterized by the competitive binding assay, and the obtained structure–activity relationships (SARs) are subsequently discussed. Omitting the bulky 6-TAMRA moiety of **1a** (*K*_d_ = 0.94 nM), as in **1c** (*K*_d_ = 1.51 nM), or replacing it with an acetyl moiety, as in **1d** (**BCY8126**, *K*_d_ = 0.73 nM), had no significant influence on the binding affinity. Similarly, the N-terminal replacement with (*R*)-NODAGA was well tolerated (**1e**, *K*_d_ = 0.77 nM). The ^nat^Cu- and ^nat^Ga-complexes of **1e** were also prepared and characterized for their binding to nectin-4. While ^**nat**^ **Cu-1e** exhibited a comparable binding affinity (*K*_d_ = 0.23 nM) to **1e**, the binding affinity of ^**nat**^ **Ga-1e** was significantly lower (*K*_d_ = 4.47 nM). It is worth noting in this context that the Ga-NODAGA and Cu-NODAGA complexes differ not only in the charge (±0 vs −1), but likely also in the complex geometry.^43,44^ Apart from (*R*)-NODAGA, we were also interested in testing other chelating units, which are suitable for labeling with ^64^Cu and ^68^Ga. For this purpose, compound **1f** was prepared, which bears DOTA. The presence of DOTA led to a 10-fold lower binding affinity (*K*_d_ = 7.22 nM) compared to the (*R*)-NODAGA pendant, which indicates that the size of the chelating unit and thus the potential orientation of the carboxylate groups are somehow crucial for binding to nectin-4.

Upon ^64^Cu- and ^68^Ga-labeling of **1e**, the formation of a radiolabeled side-product was noted (see below), which we interpreted to originate from the oxidation of the methionine at position 4 to the respective methionine sulfoxide. To verify this hypothesis, an authentic peptide with a methionine sulfoxide residue was synthesized (**2**). Furthermore, we assessed its binding affinity to nectin-4 and were surprised to see that this peptide was 34-fold less potent (*K*_d_ = 25.0 nM) compared to its methionine pendant. Previously, a similar result was obtained by Mudd et al.^17^ for a nonoptimized bicyclic precursor of **BT8009** with either methionine or methionine sulfone in position 4 of the peptidic scaffold. However, only a 2.5-fold drop in binding affinity was observed for the peptide with methionine sulfone. Given the susceptibility of the methionine residue in **1e** to oxidation during the synthesis and radiolabeling, a major motivation of the present work from a (radio)­chemical perspective was the replacement of this particular amino acid by bioisosteres that are less or not prone to such a transformation. Close analogs of methionine are norleucine (Nle, **3a**) and methoxinine (Mox, **4**) in which the −S− group is replaced by −CH_2_– or −O–, respectively. The characterization with the FA assay revealed binding affinities to nectin-4 comparable to that of **1e** (*K*_d_ values of 1.04 and 1.64 nM for **3a** and **4**, respectively). For the sake of completeness, we also prepared the bicyclic peptide with selenomethionine (SeMet) in position 4 (**5**), although it is known that selenomethionine is more readily oxidized to the respective selenoxide,^45,46^ which was also noted herein during the synthesis and isolation of **5** (see above). Despite a significant contamination of product **5** with the respective selenoxide, even after purification by RP-HPLC, its binding affinity was assessed, revealing a *K*_d_ value of 1.37 nM. Consequently, all bioisosteric methionine replacements in position 4 were equally recognized by nectin-4 and comparable to methionine itself. For compound **3a** with norleucine in position 4, we also prepared the ^nat^Cu-complex (^**nat**^ **Cu-3a**) and replaced (*R*)-NODAGA by an acetyl residue (**3b**). These changes were similarly well tolerated for nectin-4 binding, as observed for **1e**. In addition to methionine analogs with a heteroatom at the same side-chain position, *S*-ethylcysteine (Cys­(et), **6**) and *O*-ethylserine (Ser­(et), **7**) were considered as potential bioisosteric replacements for methionine. However, the binding affinities of these two compounds were approximately 10-fold lower than that of the methionine analogue **1e**, suggesting that an electron-donating heteroatom should either be absent from the side-chain or ideally positioned at the δ position to achieve a favorable binding affinity to nectin-4.

During our work on radiolabeled nectin-4 ligands, Duan et al.^38^ reported preclinical and clinical data for a radiolabeled bicyclic peptide derived from **BT8009**, called [^**68**^ **Ga]­Ga–N188**. This peptide bears DOTA as an N-terminal group but features also two amino acid changes compared to the original bicyclic scaffold of **BT8009**: substitution of 1-naphthylalanine (1NaI) by 2-naphthylalanine (2NaI) in position 2 and homoarginine (hArg) for arginine (Arg) in position 5. Furthermore, **N188** bears a free C-terminus compared to the C-terminal primary amide of **BT8009**. Unfortunately, the particular reasons for these structural changes were not provided by the authors. To shed light on the potential implications of the amino acid substitutions on nectin-4 binding, we synthesized a compound series **8**, which shares the 2NaI^2^/Arg^5^ residues and has (*R*)-NODAGA (**8a**), 6-FAM (**8b**), or DOTA (**8c**) incorporated. Furthermore, compound **8d** was synthesized, which is identical to **N188** and differs from **8c** only in the kind of C-terminus (−CONH_2_ for **8c** and −COOH for **8d**). Compared to compound **1e** with 1NaI^2^/hArg^5^ and NODAGA, the binding affinities of **8a** (*K*_d_ = 21.0 nM) and **8c** (*K*_d_ = 149 nM) were reduced by factors of 30 and 213. In line with these results, **8c** and **8d** (**N188**) were also less potent (factors of 21 and 26, respectively) than the DOTA conjugate **1f** bearing 1NaI^2^/hArg^5^. In this context, Duan et al. determined a binding affinity for **N188** of 23.7 nM, which was determined by SPR spectroscopy. The discrepancy in the present data might originate from the different buffer conditions (pH 7.4 herein vs 4.0). In accord with the results for **8a** and **8c**, the 6-FAM conjugate **8b** exhibited an 8-fold lower binding affinity (determined by direct binding to nectin-4) compared to the 6-FAM conjugate **1b**. To characterize the single substitutions with either 2NaI or Arg, compounds **9** and **10** were synthesized (both with (*R*)-NODAGA) and characterized. While the substitution of hArg by Arg was well tolerated (*K*_d_ = 0.61 nM for **10**), the substitution of 1NaI by 2NaI caused a significant decrease in the binding affinity (*K*_d_ = 11.5 nM for **9**). Accordingly, the lower binding affinity of **8a** compared to **1e** might be mainly a result of different regioisomeric naphthylalanines incorporated into the peptidic scaffold.

For the phage screening approach used to identify the bicyclic scaffold of **BT8009**, the bicyclic peptide library was screened against the soluble extracellular domain of nectin-4, with the exact binding site not yet explored.^17^ In contrast, for the antibody enfortumab, it was shown that the binding site is located within the V-domain of nectin-4.^24^ To shed light on the potential binding site of the bicyclic peptides, we envisaged the competition of probe **1a** with enfortumab. Indeed, enfortumab displaced probe **1a** in a dose-dependent manner, and a *K*_d_ value of 0.26 nM (0.26–0.29 nM for a 68% confidence interval) has been calculated. Furthermore, we measured the binding of **1a** to the recombinant N-terminal Ig-like V domain of nectin-4. A *K*_d_ value of 1.30 nM (0.78–2.12 nM for a 68% confidence interval) was determined, which is close to the value determined for the complete extracellular domain of nectin-4 (0.94 nM). Consequently, the bicyclic peptides bind at the Ig-like V domain of nectin-4, and their binding site overlaps at least with the binding site of enfortumab.

To verify the binding affinities determined with the FA assay and to get insight into the underlying changes of association and dissociation that lead to the different binding affinities, we sought to establish a SPR method. For this purpose, recombinant human nectin-4 was covalently immobilized on a CM5 sensor chip by EDC/NHS coupling. For analyses, the buffer composition (HBS-P+, pH 7.4) was similar to that of the FA assay; however, DMSO was completely omitted (1% DMSO for the FA assay). Selected compounds were characterized by SPR, and the data for *K*_d_, *k*_on_, and *k*_off_ are summarized in Table 2. An exemplary sensorgram for compound **4** is shown in Figure 3C (for the other compounds, see Figure S3), and a *k*_on_-*k*_off_ map is depicted in Figure 3D.

**Table 2:** Summary of Binding Data to Recombinant Nectin-4

compound	N-terminal group	AA changes^a^	*K*_d_ (FA, nM)^b^	*K*_d_ (SPR, nM)^c^	*k*_on_ (SPR, *10^6^ M^–1^ s^–1^)^c^	*k*_off_ (SPR, *10^–3^ s^–1^)^c^
**1a**	6-TAMRA-		0.94 (0.09)	n.d.	n.d.	n.d.
**1b**	6-FAM-		0.17 (0.01)	n.d.	n.d.	n.d.
**1c**	NH_2_-		1.51 (1.46–1.56)	n.d.	n.d.	n.d.
**1d (BCY8126)**	Acetyl-		0.73 (0.69–0.77)	n.d.	n.d.	n.d.
**1e**	NODAGA-		0.77 (0.54–0.99)	3.60	1.21 (±0.06)	4.34 (±0.14)
^**nat**^ **Cu-1e**	NODAGA-		0.23 (0.15–0.32)	n.d.	n.d.	n.d.
^**nat**^ **Ga-1e**	NODAGA-		4.47 (4.16–4.84)	2.39	1.10 (±0.05)	2.60 (±0.19)
**1f** ^d^	DOTA-		7.22 (5.41–11.0)	1.31	8.76	11.5
**2**	NODAGA-	MetO^4^	25.0 (24.2–26.1)	n.d.	n.d.	n.d.
**3a**	NODAGA-	Nle^4^	1.04 (0.88–1.23)	1.31	6.73 (±0.20)	7.59 (±0.26)
^**nat**^ **Cu-3a**	NODAGA-	Nle^4^	1.22 (1.13–1.32)	n.d.	n.d.	n.d.
**3b**	Acetyl-	Nle^4^	0.58 (0.50–0.67)	n.d.	n.d.	n.d.
**4** (**NECT-224**)	NODAGA-	Mox^4^	1.64 (1.50–1.79	5.17	1.40 (0.13)	7.24 (±0.56)
**5**	NODAGA-	SeMet^4^	1.37 (1.21–1.55)	n.d.	n.d.	n.d.
**6**	NODAGA-	Ser(et)^4^-	16.4 (14.6–18.8)	6.29	3.86 (±0.29)	24.7 (±3.95)
**7**	NODAGA-	Cys(et)^4^-	6.01 (4.76–8.64)	6.19	4.61 (±0.93)	26.8 (±4.43)
**8a**	NODAGA-	2Nal^2^/Arg^5^	21.0 (16.2–40.9)	63.0	0.68 (0.05)	43.3 (±5.23)
**8b**	6-FAM	2Nal^2^/Arg^5^	1.38 (0.06)	n.d.	n.d.	n.d.
**8c** ^d^	DOTA-	2Nal^2^/Arg^5^	149 (132–175)	127	0.02 (±0.01)	2.39 (±0.46)
**8d (N188)**	DOTA-	2Nal^2^/Arg^5^	191 (179–204)	236	0.04 (±0.01)	8.81 (±0.05)
**9**	NODAGA	2NaI^2^	11.5 (11.0–11.9)	4.58	16.9 (±2.24)	76.5 (11.8)
**10**	NODAGA-	Arg^5^	0.61 (0.56–0.66)	2.51	1.11 (±0.03)	2.78 (0.13)

a Amino acid (AA) changes compared to the sequence Cys-Pro^1^-1NaI^2^-d-Asp^3^-Cys-Met^4^-hArg^5^-Asp^6^-Trp^7^-Ser^8^-Thr^9^-Pro^10^-Hyp^11^-Trp^12^-Cys-CONH_2_. Additionally, compound **8d** (**N188**) has a free C-terminus.

b Dissociation constants determined by the FA-based (competition) assay. Data shown are mean values of one (**9**), two or three (**1a**, **1b**, **8b**) separate experiments (each performed in duplicate) with estimated confidence interval (68.3%) in parentheses (IC_50_ values and Hill coefficients in Table S1). For compounds **1c** and **1d**, mean values (±SEM) are shown.

c Binding data (*K*_d_, *k*_on_, and *k*_off_) determined by SPR. Data shown are mean values (±SEM) of at least 3 separate experiments. For compound **1f**, the results of only one experiment are shown (Supporting Information). n.d. denotes not determined.

d SPR sensorgrams for these compounds showed a first rapid dissociation phase followed by a second slower dissociation phase, leading to deviations from ideal 1:1 binding (see Figure S4 for further discussion).

Regarding the binding affinities, the overall trend determined with the FA assay was confirmed by the SPR analyses, although the absolute *K*_d_ values tended to be higher. Compounds **1e**, ^**nat**^ **Ga**-**1e**, **3a**, **4**, and **10** exhibited similar *K*_d_ values in the range of 1.3–5.2 nM and also similar values for the rate constants *k*_on_ (1.1–6.7 × 10^6^ M^–1^ s^–1^) and *k*_off_ (2.6–7.6 × 10^–3^ s^–1^). Although the binding affinities of compounds **6** and **7** were only slightly lower compared to those of the aforementioned five compounds, their rate constants markedly increased, with the *k*_off_ values being 24.7 and 26.8 × 10^–3^ s^–1^. Furthermore, the single substitution of 1NaI by 2NaI (**1e** vs **9**), which lowered the binding affinity but to a lesser extent than observed in the FA assay, led to an 18-fold increase in *k*_off_ (and also *k*_on_). This increase in the rate constants was also conserved for the double substitution (**1e** with 1NaI^2^/hArg^5^ vs **8a** with 2NaI^2^/Arg^5^), albeit the increase in *k*_on_ was less pronounced, which resulted in a worse binding affinity (*K*_d_ = *k*_off_/*k*_on_). It is striking that **8c** and **8d** have the lowest binding affinities of all compounds investigated; however, their *k*_off_ values (2.39 and 8.81 × 10^–3^ s^–1^, respectively) are similar to that of **1e**, meaning that the low binding affinity is a result of a comparably low *k*_on_ value. Furthermore, the detrimental effect of 2NaI in combination with (*R*)-NODAGA on the *k*_off_ value is somehow compensated by substituting (*R*)-NODAGA with DOTA. The implications of the determined binding affinities and, in particular, the rate constants for association and dissociation are further discussed in the context of the PET imaging data.

### Conformational Analyses

To rationalize the observed SARs for the series of nectin-4 targeting peptides herein, a molecular docking approach would be worth performing. In this context, crystal structures of one or two of the three Ig-like extracellular domains of nectin-4 are available.^47,48^ In view of a potential molecular docking approach, we sought to determine the solution structure of selected peptides. To get a first impression of the presence of a defined secondary structure, ECD spectra were recorded for the peptides **1d** and **1e** in acetonitrile/water (1:1, v/v) in the absence and presence of 33% trifluoroethanol (Figure S5). The moderate solubility of both compounds in pure water required the addition of acetonitrile as an organic cosolvent. The spectra of both compounds showed a strong negative maximum at 203 nm, which is slightly shifted to 202 nm in the presence of trifluoroethanol. Additionally, there is a weak shoulder visible in the presence of trifluoroethanol at ≈212 nm for **1d** and 217 nm for **1e**. Accordingly, the shape of the curves resembles the common ECD signature for a 3_10_-Helix.^49^ To support this finding and to obtain more detailed information on the secondary structure, we attempted to determine the solution conformation of **1d** by 1D and 2D ^1^H NMR experiments. Again, due to solubility issues in water, these experiments were performed in DMSO-*d*_6_. On the basis of TOCSY and supported by COSY, HSQC, HMBC, and ROESY experiments, a preliminary assignment of the ^1^H signals to the distinct amino acid residues has been conducted (Table S2). The sequence-specific assignment was complicated as 9 out of the 15 amino acids exhibit the same spin system NH-αH-βH (3 × Cys, 1NaI^2^, d-Asp^3^, Asp^6^, 2 × Trp^7/9^, Ser). Furthermore, no interactions could be deduced between N_α_H signals based on the NOE data, and only a few between N_α_H and C_α_H of different amino acid residues. Consequently, a structure determination based on the NMR data was not possible. In this context, in their first report on displaying bicyclic peptides on phages, Heinis et al.^13^ also described NMR attempts to elucidate the solution structure of the identified plasma kallikrein inhibitor **PK15**, which is a bicyclic peptide in 6 × 6 format. Although a sequence-specific assignment was possible, their NOE data obtained in aqueous media provided no evidence of interactions between the loops, no NOEs across the loops, and no evidence of short segments with regular secondary structures. For compound **1d**, it appears that, for a given residue, distinct sets of signals arise from interaction with DMSO as a hydrogen bond acceptor, as inferred from Thr and Ser OH 1H resonance at δ_H_ > 12 ppm. Especially at low temperature and in residues possessing hydrogen bond donor sites (e.g., indole NH in Trp as well as OH in Ser and Thr), similar but distinguishable spin systems are present, further complicating signal assignment (Figures S6 and S7). However, these observations underscore the peptide’s ability (and potential sites) for specific and meaningful intermolecular hydrogen bonds.

### Radiolabeling and Radiopharmacological Characterization In Vitro

#### ^64^Cu- and ^68^Ga-Labeling of Selected Peptides

Labeling of the peptides with ^64^Cu was performed with in-house produced [^64^Cu]­CuCl_2_ ^50^ in ammonium acetate buffer (pH of 5.6) for 20 min at 60 °C. Labeling of peptides with ^68^Ga was achieved using generator produced [^68^Ga]­GaCl_3_ in a sodium acetate buffer (pH of 4.5) for 10 min at 90 °C. In all cases, the incorporation of [^64^Cu]­Cu^2+^ and [^68^Ga]­Ga^3+^ proceeded with a yield of ≥97% (Supporting Information). However, ^64^Cu-labeling of the methionine-bearing peptides **1e** and **8a** resulted in the partial formation of a radiolabeled side-product of lower retention time (6.6 ± 3.4% for **[** ^**64**^ **Cu]­Cu-1e**, *n* = 12) as observed from radio-HPLC analysis (Figure 4A). We hypothesized that this side-product originates from oxidation of the methionine residue to methionine sulfoxide, which was already observed during the synthesis of **1e**. Previously, such an oxidation has been reported for the human gastrin derivative MG11 upon ^177^Lu-labeling.^51^ To identify the side-product herein, the authentic peptide **2** with a methionine sulfoxide residue was synthesized and labeled with ^64^Cu. Indeed, the chromatographic comparison by means of retention time and coinjection of **[** ^**64**^ **Cu]­Cu-1e** and **[** ^**64**^ **Cu]­Cu-2** confirmed that **[** ^**64**^ **Cu]­Cu-2** is the radiolabeled side-product formed upon ^64^Cu-labeling of **1e** (Figure 4A). In this context, for **2** and **[** ^**64**^ **Cu]­Cu-2**, a rather broad peak was observed in the (radio)-HPLC chromatograms, which we reasoned to originate from the chirality of the sulfoxide group, which, in turn, results in an epimeric mixture. As expected, for the peptides with norleucine (**3a**), methoxinine (**4**, Figure 4B), or *O*-ethylserine (**6**), no radiolabeled side-products were observed. It is worth noting that also for peptide **7** with *S*-ethylcysteine, there was no evidence for partial oxidation during radiolabeling (Figure S8), which indicates that the methylthioether group is particularly amenable to oxidation. For **[** ^**64**^ **Cu]­Cu-5**, which bears selenomethionine, the proportion of the respective radiolabeled side-product was even more pronounced; however, compound **5** already contained a considerable amount of the respective selenoxide as discussed above.

**Figure 4:**
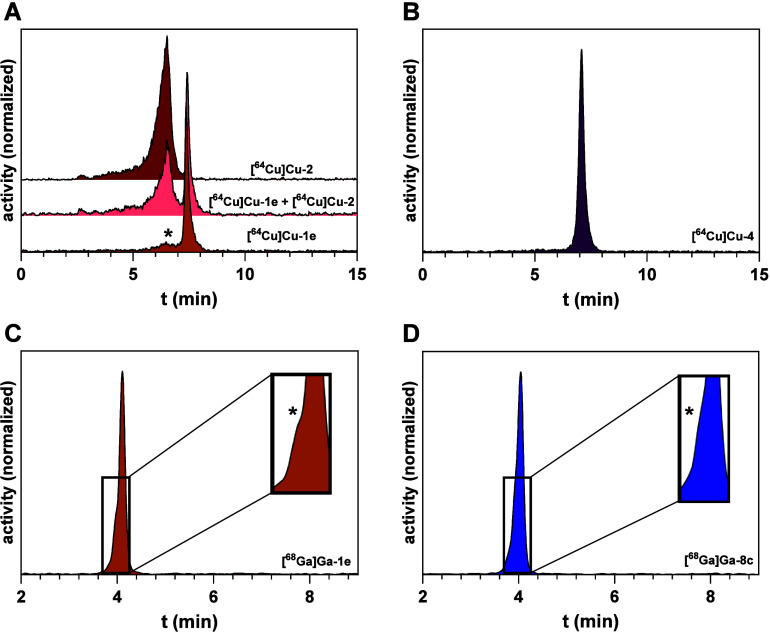
Radio-HPLC analysis after ^64^Cu- and ^68^Ga-labeling of different peptides. (A) Analytical radio-HPLC chromatograms of **[^64^Cu]­Cu-1e**, its methionine sulfoxide analog **[^64^Cu]­Cu-2**, and a mixture of both radiolabeled peptides, demonstrating that the radiolabeled side-product formed during ^64^Cu-labeling of **1e** (marked with an asterisk) corresponds to the respective methionine sulfoxide analog **[^64^Cu]­Cu-2**. (B) Analytical radio-HPLC chromatogram of **[^64^Cu]­Cu-4** with a symmetric peak shape. (C,D) Analytical radio-HPLC chromatograms of **[^68^Ga]­Ga-1e** (C) and **[^68^Ga]­Ga-8c** (D) with the insets showing the asymmetric peak shape for both radiolabeled peptides, which might be indicative of the partial oxidation.

The phenomenon of partial oxidation was also noticed upon ^68^Ga-labeling of **1e**, although the formation of the radiolabeled methionine sulfoxide side-product could only be concluded from an asymmetric peak shape of the actual radiolabeled product (Figure 4C). A similar result was obtained for the radiosynthesis of [^**68**^ **Ga]­Ga-8c** (Figure 4D) and **[** ^**68**^ **Ga]­Ga-8d**. Although all previous radiolabeled bicyclic peptides targeting nectin-4 maintained methionine in position 4, as reported for **BT8009**,^38,39,52–55^ the issue of partial oxidation has not been discussed so far. However, a close inspection of the reported radio-HPLC chromatograms indicates that at least for some ligands, this side-reaction might also have occurred as radiolabeled side-products can be suspected after radiolabeling.^38,52,55^

#### Stability, Plasma Protein Binding, and log*D*_7.4_ and CHI-IAM Values

To further demonstrate the increased stability toward oxidizing conditions upon methionine substitution, we sought to incubate the radiolabeled peptides in H_2_O_2_ solution, followed by radio-HPLC analysis at different time points (Figure S9). To avoid a too rapid degradation of radiolabeled peptides, a low H_2_O_2_ concentration of 0.0012% was chosen. Under these conditions, the degradation of the radiolabeled peptides was clearly not limited to monooxidation, as an additional broad peak pattern was observed in the radio-HPLC chromatograms for all compounds studied (Figure S9). In this context, there are three further thioether functionalities in each peptide due to the cyclization of the Cys residues with TATA. In Figure 5, the curves for the time-dependent degradation of the intact radioligands are depicted, from which the respective half-lives were calculated. Among the characterized compounds, **[** ^**64**^ **Cu]­Cu-3a** with norleucine at position 4 exhibited the best stability with a half-life of 17.4 h. Interestingly, for **[** ^**64**^ **Cu]­Cu-4** and **[** ^**64**^ **Cu]­Cu-7** with methoxinine and *S*-ethylcysteine in position 4, similar degradation half-lives were determined (6.87 and 8.81 h, respectively). For the methionine-containing peptides **[** ^**64**^ **Cu]­Cu-1e**, **[** ^**64**^ **Cu]­Cu-8a** and **[** ^**64**^ **Cu]­Cu-10** half-lives between 3.5 and 4.0 h have been calculated, while for **[** ^**64**^ **Cu]­Cu-5** with selenomethionine in position 4, a half-life of only 23 min (0.38 h) was obtained, which is by a factor of 10 shorter than for its methionine pendant **[** ^**64**^ **Cu]­Cu-1e**. This finding is consistent with previous data comparing the susceptibility of methionine and selenomethionine to oxidative conditions.^45^ Consequently, the applied conditions appeared to mimic the susceptibility of methionine and its bioisosteres to oxidation, and a significantly improved stability was achieved by introducing norleucine, methoxinine, or *S*-ethylcysteine.

**Figure 5:**
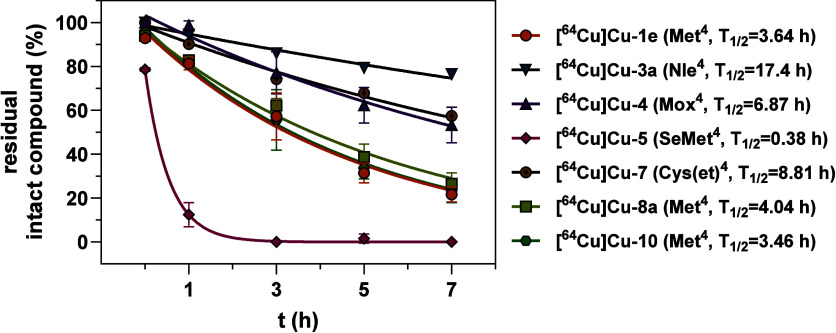
Time-dependent degradation of selected radiolabeled peptides in the presence of H_2_O_2_. Plots of residual intact radiolabeled peptide, which was assessed by analytical radio-HPLC, as a function of time. Radiolabeled peptides were incubated in the presence of 0.0012% H_2_O_2_ at 25 °C. The amino acids in position 4 of the bicyclic scaffold, together, with the half-lives determined by nonlinear regression according to one-phase decay, are given in parentheses after the compound names. Data shown are mean values (±SD) of one (**[^64^Cu]­Cu-3a** and **[^64^Cu]­Cu-7**), two (**[^64^Cu]­Cu-5**, **[^64^Cu]­Cu-8a**, and **[^64^Cu]­Cu-10**) or three (**[^64^Cu]­Cu-1e** and **[^64^Cu]­Cu-4**) separate experiments, each performed in single execution.

Apart from challenging the radiolabeled peptides under artificial conditions (H_2_O_2_), the chemical and proteolytic stability of ^64^Cu-labeled **1e**, **3a**, and **4** was investigated upon incubation in human plasma and PBS (pH 7.4) at 37 °C for up to 24 h. No signs of degradation, including ongoing oxidation, were observed in both media (Figure S10). The assessment of binding to plasma proteins and isolated human serum albumin (HSA) was exemplarily performed for **[** ^**64**^ **Cu]­Cu-1e** and **[** ^**64**^ **Cu]­Cu-4** by ultrafiltration, which revealed a negligible binding capability to HSA or other plasma proteins (Figure S11). Furthermore, in the course of their radiopharmacological characterization, the partition coefficient (log*D*_7.4_) between octanol and PBS (pH 7.4) was determined for selected ^64^Cu- and ^68^Ga-labeled peptides. The obtained values are summarized in Table 3. Irrespective of the complexed radiometal ion, all peptides are hydrophilic molecules with log*D*_7.4_ values below −2.0. However, while the substitution of methionine by methoxinine did not affect the partition coefficient (−2.74 and −2.76 for **[** ^**64**^ **Cu]­Cu-1e** and **[** ^**64**^ **Cu]­Cu-4**, respectively), the substitution by norleucine or methionine sulfoxide led to a slight increase of 0.2 log units (−2.53 and −2.51 for **[** ^**64**^ **Cu]­Cu-3a** and **[** ^**64**^ **Cu]­Cu-2**). An even more pronounced increase in the log*D*_7.4_ value was exerted by selenomethionine (−2.37 for **[** ^**64**^ **Cu]­Cu-5**). The substitution of hArg^5^ by Arg^5^ also seems to increase the log*D*_7.4_ value as seen for **[** ^**64**^ **Cu]­Cu-8a** and **[** ^**64**^ **Cu]­Cu-10**.

**Table 3:** Summary of log*D*_7.4_ Values and Saturation Binding Data for Different ^64^Cu- and ^68^Ga-Labeled Nectin-4 Ligands

	^64^Cu-labeled			^68^Ga-labeled		
compound^a^	log*D*_7.4_ ^b^	*K*_d_ (nM)^c^	*B*_max_ (fmol/mg)^c^	log*D*_7.4_ ^b^	*K*_d_ (nM)^c^	*B*_max_ (fmol/mg)^c^
**1e**	–2.74 (±0.09)	7.15 (±0.71)	780 (±23.9)	–2.96 (±0.01)	8.70 (±1.01)	1345 (±52.5)
**2**	–2.51 (±0.22)	n.d.	n.d.	n.d.	n.d.	n.d.
**3a**	–2.53 (±0.05)	n.d.	n.d.	n.d.	n.d.	n.d.
**4** (**NECT-224**)	–2.76 (±0.07)	6.06 (±0.66)	378 (±12.0)	–2.74 (±0.00)	13.1 (±1.34)	1510 (±58.4)
**5**	–2.37 (±0.07)	n.d.	n.d.	n.d.	n.d.	n.d.
**8a**	–2.48 (±0.01)	51.7 (±8.36)	215 (±16.1)	n.d.	n.d.	n.d.
**8c**	n.d.	n.d.	n.d.	–2.79 (±0.05)	103 (±29.9)	799 (±136)
**8d (N188)**	n.d.	n.d.	n.d.	–3.14 (±0.24)	222 (±86.0)	291 (±77.9)
**10**	–2.30 (±0.10)	n.d.	n.d.	n.d.	n.d.	n.d.

a After ^64^Cu- or ^68^Ga-labeling, the excess of unlabeled ligand was not separated or saturated with ^nat^Cu^2+^ or ^nat^Ga^3+^.

b Data shown are mean values (±SD) of three separate processes of shaking out.

c Data shown are mean values (±SD) of two separate experiments for the ^64^Cu-labeled ligands and one experiment for the ^68^Ga-labeled ligand, with each experiment being performed in quintuplicate using intact HT-1376 cells. n.d. denotes not determined.

To characterize the peptides regarding their potential nonspecific binding to membranes and nontarget proteins, which can be a limiting characteristic for radioligands, we determined the chromatographic hydrophobicity index values at pH 7.4 (CHI IAM_7.4_) for selected nonlabeled peptides (**1e**, ^**nat**^ **Cu-1e**, **3a**, ^**nat**^ **Cu-3a**, **4**, **5**, **8a**, and **10**) by HPLC (Table S3).^56,57^ All compounds exhibited low CHI IAM_7.4_ values with slightly higher values being observed for the ^nat^Cu-complexes (23.4 and 23.8 for ^**nat**^ **Cu-1e** and ^**nat**^ **Cu-3a**, respectively) compared to the metal-free complexes (<20.6). Consequently, the CHI IAM_7.4_ values indicate that unfavorable pharmacokinetic behavior due to nonspecific binding is not to be expected.

#### Nectin-4 in Urothelial Cell Models

For the radiopharmacological characterization of the radiolabeled peptides, suitable cell lines had to be identified. Based on previous data regarding nectin-4 abundance in urothelial cancer cell lines,^36,53^ the urothelial carcinoma cell lines HT-1376 and 5637 were selected. The presence of nectin-4 was verified by Western blot analysis, immunofluorescence staining, and ELISA as complementary methods (Figure 6). While immunoblotting showed that 5637 cells were nectin-4-negative, two protein bands were identified in HT-1376 cell lysates: one between 55 and 70 kDa, and another slightly above 70 kDa (Figure 6A). These results are in line with Western Blot data of nectin-4-positive cells provided by different vendors of nectin-4 antibodies.^54,55^ Protein bands appearing at a molecular mass higher than predicted for nectin-4 (55 kDa, 510 amino acids) might originate from glycosylation.^21^ The subcellular location at the plasma membrane of the HT-1376 cells was confirmed by immunofluorescence staining (Figure 6B). ELISA data corroborated the results from Western blot analysis and showed a nectin-4 content of approximately 70 fmol/mg in the HT-1376 whole-cell lysate (Figure 6D). Nectin-4-specific binding to HT-1376 was further proven for the 6-FAM-labeled fluorescent probe **1b** by flow cytometry (Figure 6C). Overall, HT-1376 cells appear to be suitable to evaluate target-specific binding of nectin-4-directed radiolabeled peptides, and 5637 cells will serve as negative controls.

**Figure 6:**
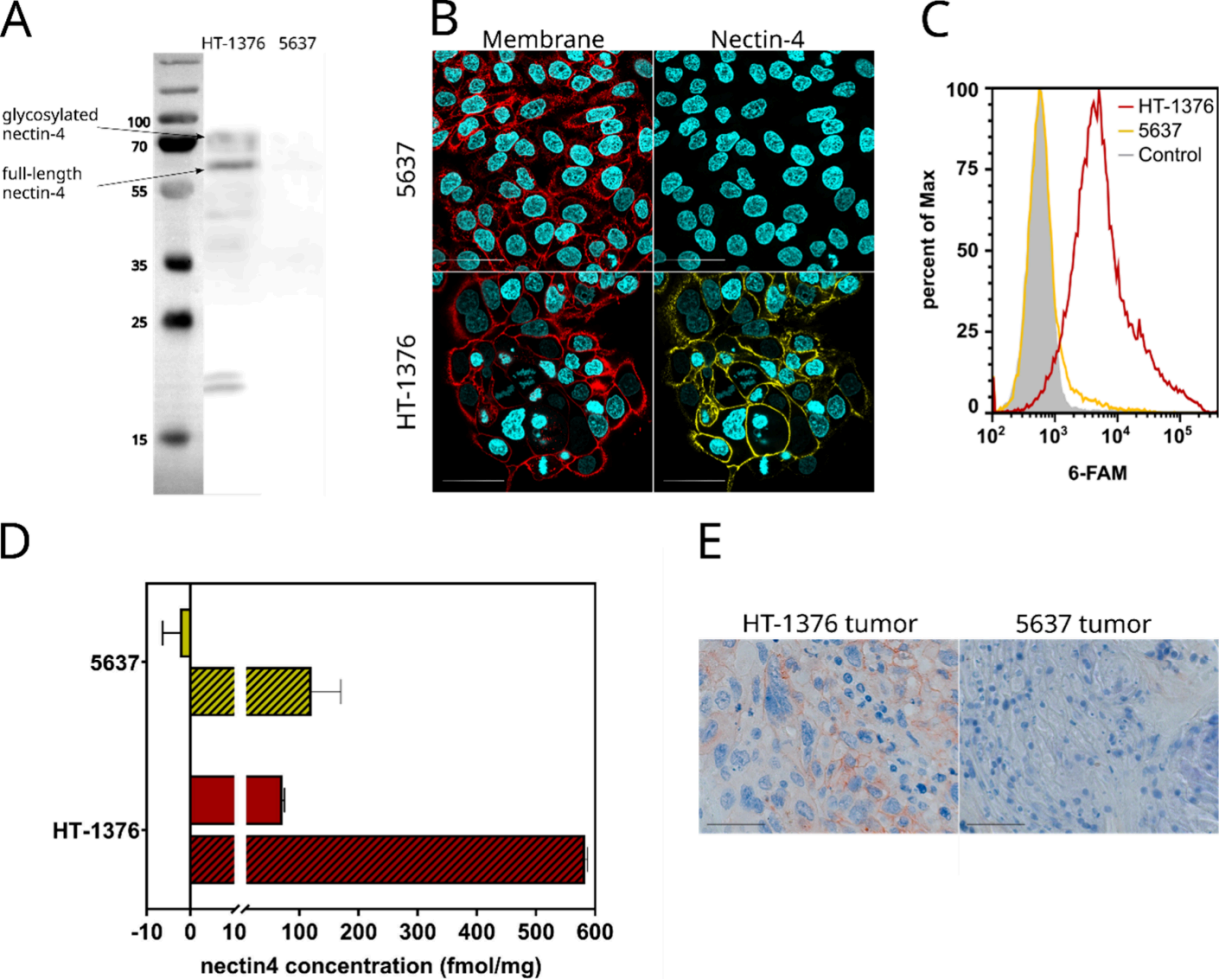
Nectin-4 status of urothelial carcinoma cell lines HT-1376 and 5637. (A) Exemplary immunoblots of HT-1376 and 5637 cell lysates. The Thermo Scientific PageRuler Plus Prestained Protein Ladder was acquired with white light illumination and automatically merged to the chemiluminescent image (imager Celvin S). (B) The presence and distribution of nectin-4 in HT-1376 and 5637 cells were visualized by immunofluorescence staining. Nectin-4 is depicted in yellow, while the cell membrane is shown in red, and the cell nuclei are in cyan. (C) FACS histogram of 5637 (yellow) and HT-1376 cells (red) stained with probe **1b**. As a negative control, unstained HT-1376 cells are shown in gray. (D) ELISA results for the nectin-4 concentrations in HT-1376 and 5637 cell lysates (solid bars) and tumor lysates (hatched bars). (E) Immunohistochemical staining of nectin-4 in HT-1376 and 5637 tumor sections. Hematoxylin staining of cell nuclei in blue and immunohistochemical staining of nectin-4 in red. All scale bars indicate 50 μm. Immunostaining with antibody isotype controls is provided in the Supporting Information (Figure S12).

#### Cell Binding, Internalization, and Cellular Release

For the subsequent radiopharmacological characterization, compounds **1e**, **3a**, **4**, **8a**, **8c**, and **8d** (**N188**) were selected as this panel of nectin-4 ligands enables the comparative investigation of the influence of methionine and two of its bioisosteric replacements (norleucine and methoxinine) on nectin-4 targeting (in vitro and in vivo). In fact, the radiolabeled pairs **[** ^**64**^ **Cu]­Cu-1e**/**[** ^**68**^ **Ga]­Ga-1e** and **[** ^**64**^ **Cu]­Cu-4**/**[** ^**68**^ **Ga]­Ga-4** were characterized, while **3a** was only used as ^64^Cu-labeled analog. Furthermore, compound **[** ^**68**^ **Ga]­Ga-8c**, an analog of **[** ^**68**^ **Ga]­Ga–N188** differing only in the kind of the C-terminus, and **[** ^**68**^ **Ga]­Ga-8d** (**[** ^**68**^ **Ga]­Ga–N188**) were included. As the [^64^Cu]­Cu-DOTA complex does not provide sufficient kinetic inertness in mice (resulting in an increased activity uptake in the liver),^58,59^ compound **8a**, which is based on the same bicyclic scaffold as **8c** but harbors NODAGA instead of DOTA, was used for ^64^Cu-labeling, and the resulting radioligand was characterized herein.

To identify a suitable time point for assessing specific cell binding of the radioligands, the time-dependent binding of **[** ^**64**^ **Cu]­Cu-4** to HT-1376 cells (at 10 nM) was characterized at 37 °C (Figure 7A). Surprisingly, even after 4 h, the binding to HT1376 cells did not reach a plateau. As the binding of the Bicyclic peptides to nectin-4 underlies a fast equilibrium, as deduced from the FA and SPR assay data, we hypothesized that the increase in total bound radioligand over 4 h might indicate a continuous internalization process of the radioligand. To support this, time-dependent binding was also investigated after washing the cells with acidic glycine buffer (pH 2.8), which should allow for discriminating between surface-bound and internalized fractions of total bound radioligand (after PBS wash). Indeed, the fraction of surface-bound radioligand remained largely constant over 4 h at both temperatures. Furthermore, the binding capacity over the entire time range (mean of 73 fmol/mg at 37 °C) corresponds to the nectin-4 level determined with the ELISA (70 fmol/mg), indicating that the applied concentration of free **[** ^**64**^ **Cu]­Cu-4** (10 nM) represents already a saturation binding concentration. Consequently, our hypothesis that the increase in total bound radioligand over time originates from an increase in the fraction of internalized radioligand appears to be valid. It is worth noting that the association data suggest that only the radioligand is internalized, but not the nectin-4-radioligand complex, as otherwise the surface-bound activity should decrease over time and the total bound activity should not exceed the nectin-4 level (in case the radioligand internalization occurs exclusively via the nectin-4-radioligand complex). To shed further light on the putative internalization phenomenon, the time curves for total binding and surface-bound and internalized fraction were also recorded at 4 °C (Figure 7B). Surprisingly, the curves resemble those at 37 °C. Accordingly, the putative internalization process seems not to be affected by the low temperature. Furthermore, we also assessed the time-dependent binding to 5637 cells, but no conclusive data at both 4 °C and 37 °C were obtained (even at a radioligand concentration of 100 nM, Figure S13). The missing cell uptake in nectin-4-negative 5637 cells indicates that the internalization process requires the target protein, although nectin-4 is apparently not translocated from the cell surface upon radioligand binding.

**Figure 7:**
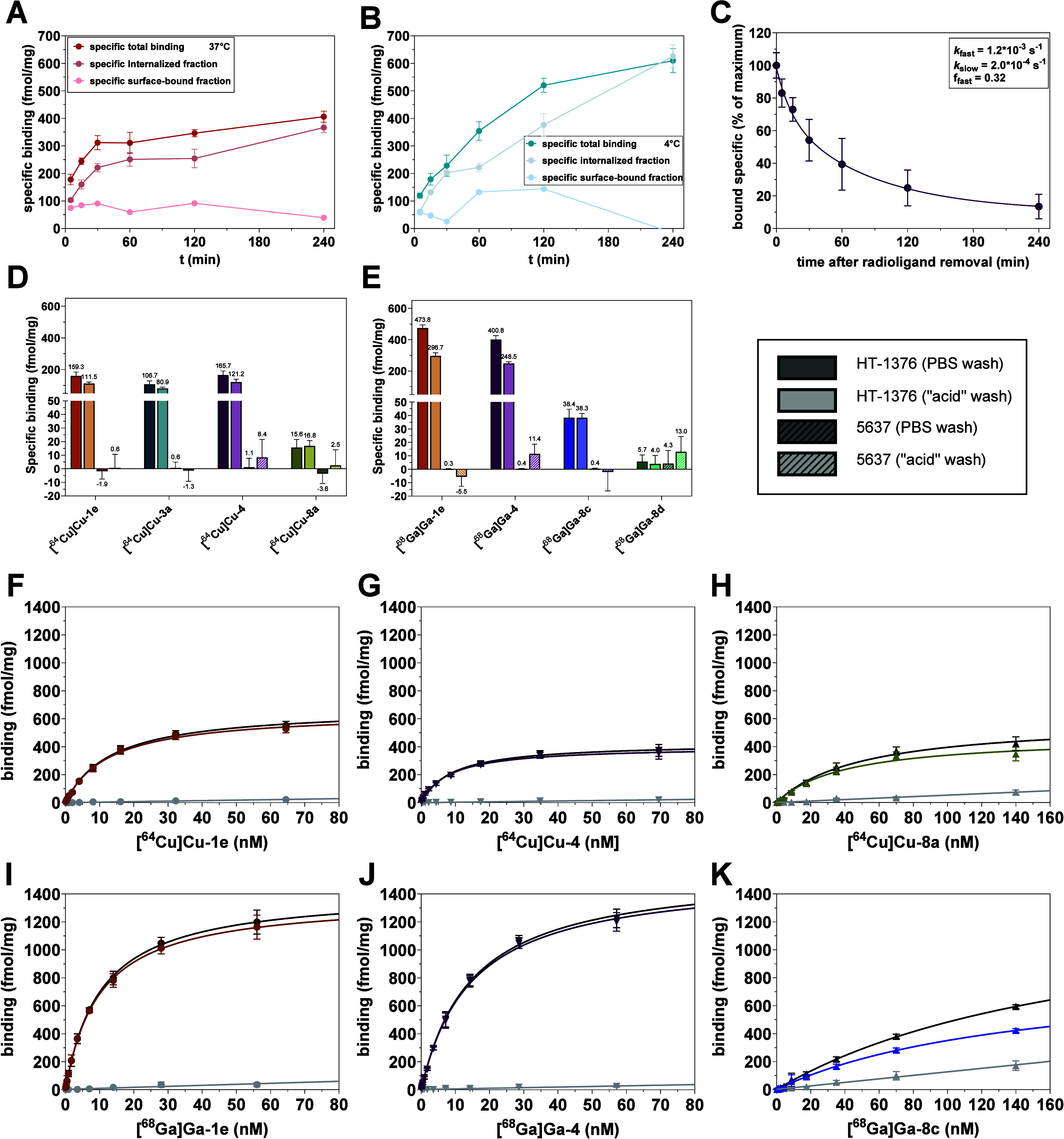
Cell binding and internalization of the nectin-4-directed radioligands. (A,B) Time-dependent binding of **[^64^Cu]­Cu-4** (10 nM) at 37 °C (A) and 4 °C (B) to HT-1376 cells. Specific total binding was obtained after washing with PBS, while the specific internalized fraction was obtained after washing with acidic glycine buffer (pH 2.8). Data shown are mean values (±SD) of one experiment, which was performed in quadruplicate. Data for the specific surface-bound fraction were calculated from specific total binding and specific internalized fractions. Nonspecific binding was assessed in the presence of 1 μM **1d** (or **3a** for **[^68^Ga]­Ga-8d**). (C) Release of **[^64^Cu]­Cu-4** from HT-1376 cells. Data shown are mean values (±SD) of two independent experiments, each performed in sextuplicates. Nonlinear regression was performed according to a two-phase decay. (D,E) Specific cell binding of the ^64^Cu- (D) and ^68^Ga-labeled (E) ligands to intact HT-1376 (muted colors) and 5637 (light colors) cells. Data are shown for specific binding after washing with PBS (solid bars) and after washing with acidic glycine buffer (“acid” wash, hatched bars), with mean values given at the bars. Data shown are mean values (±SD) of two separate experiments, each performed in sextuplicate (^64^Cu-labeled ligands), or of one experiment, which was performed in octuplicate (^68^Ga-labeled ligands). F–K) Saturation binding of **[^64^Cu]­Cu-1e** (F), **[^64^Cu]­Cu-4** (G), **[^64^Cu]­Cu-8a** (H), **[^68^Ga]­Ga-1e** (I), **[^68^Ga]­Ga-4** (J), and **[^68^Ga]­Ga-8c** (K) with data for total, nonspecific (in the presence of 1 μM **1d**) and calculated specific binding shown as black, gray, and colored circles, respectively (for **[^68^Ga]­Ga-8d** (**[^68^Ga]­Ga–N188**) see Figure S14). Regression analysis was performed as described in the experimental section. Data shown are mean values (±SD) of two separate experiments for the ^64^Cu-labeled ligands and one experiment for the ^68^Ga-labeled ligands, with each experiment being performed in quintuplicate by using intact HT-1376 cells.

To further support that the overall cell binding of **[** ^**64**^ **Cu]­Cu-4** is composed of two events, i.e., binding to nectin-4 and putative cell internalization, we characterized the release of **[** ^**64**^ **Cu]­Cu-4** from intact HT-1376 cells over 4 h after an initial incubation period of 1 h (Figure 7c). The cellular release followed a two-phase decay with half-lives of 9.5 and 83 min, respectively. Thus, the rate constant for the initial dissociation phase (*k*_fast_ = 1.2 × 10^–3^ s^–1^) is in good accordance with the determined *k*_off_ value for **4** from recombinant nectin-4 by SPR (*k*_off_ = 7.24 × 10^–3^ s^–1^). Furthermore, 68% of initially bound **[** ^**64**^ **Cu]­Cu-4** were released during the slow dissociation phase, which is also in accord with the remaining percentage of **[** ^**64**^ **Cu]­Cu-4** after treatment of intact cells with acidic glycine buffer (73%).

Overall, cell binding of **[** ^**64**^ **Cu]­Cu-4**, which we assume to be representative of the radiolabeled bicyclic peptides herein, requires the presence of nectin-4, but the extent of total binding significantly exceeds the extent expected by the amount of target protein. Our data point toward a nectin-4-dependent internalization process of the radioligand itself, which is not affected by a low temperature. In this context, the possibility to pass the cell membrane even at low temperatures is known for various linear and cyclic cell-penetrating peptides (CPPs), with mechanisms such as passive diffusion and direct translocation being discussed.^60,61^ Recently, for bicyclic peptides cross-linked with Bi^3+^ via three cysteine residues (called peptide-bismuth bicycles), efficient cell penetration even at low concentrations has been demonstrated.^62^ Beyond those peptide-bismuth bicycles, analogs with TMBM as an organic cross-linking agent also exhibited a much higher internalization compared to their linear counterparts. However, it should be noted that the bicyclic scaffolds contained at least three positive charges (overall charge of **[** ^**64**^ **Cu]­Cu-4** is −2), cell entry was significantly reduced at 4 °C, and the peptides were not designed to address any specific target protein on the cells. Further studies are needed to elucidate the cell-binding phenomenon of the nectin-4-directed bicyclic peptides herein.

For initially characterizing the nectin-4-specific cell binding of the different radioligands, we decided to use a radioligand concentration of 10 nM and an incubation period of 60 min (Figure 7D,E). This was reasoned as even at shorter incubation periods, a significant amount of radioligand is most likely internalized, and an incubation period of 60 min offers more flexibility for handling of several radioligands at once. All radioligands, with the exception of **[** ^**68**^ **Ga]­Ga-8d ([** ^**68**^ **Ga]­Ga–N188**) showed a pronounced binding to the nectin-4-positive HT-1376 cells, while no binding to the nectin-4-negative 5637 cells was discerned, confirming their nectin-4 specificity. This is consistent with the results from the binding of probe **1b** to these cells (Figure 6C). Interestingly, the ^68^Ga-labeled ligands exhibited an approximately 3-fold higher binding capacity at the chosen concentration of 10 nM compared to their ^64^Cu-labeled analogs (e.g., 159 and 474 fmol/mg for **[** ^**64**^ **Cu]­Cu-1e** and **[** ^**68**^ **Ga]­Ga-1e**, respectively). While the binding capacities of **[** ^**64**^ **Cu]­Cu-8a** and **[** ^**68**^ **Ga]­Ga-8c** (15.6 and 38.4 fmol/mg) were almost 10-fold lower compared to the ^64^Cu- or ^68^Ga-labeled analogs **1e**, **3a**, and **4**, no substantial binding of **[** ^**68**^ **Ga]­Ga-8d** (**[** ^**68**^ **Ga]­Ga–N188**) could be detected. For the radioligands **[** ^**64**^ **Cu]­Cu-1e**/**[** ^**68**^ **Ga]­Ga-1e**, **[** ^**64**^ **Cu]­Cu-4**/**[** ^**68**^ **Ga]­Ga-4**, and **[** ^**64**^ **Cu]­Cu-3a**, a fraction of ≈70% was still retained after washing with acidic glycine buffer, while for **[** ^**64**^ **Cu]­Cu-8a** and **[** ^**68**^ **Ga]­Ga-8c**, all of the total bound radioligand was acid-resistant (i.e., internalized).

To characterize the binding of the radioligands to cellular nectin-4 in more detail, saturation binding analyses using intact HT-1376 cells (after PBS wash) were performed (Figure 7F–K, and *K*_d_ and *B*_max_ are summarized in Table 3). The trend in the *K*_d_ values for the radioligands using intact cells is consistent with the trend observed for the nonlabeled peptides and recombinant human nectin-4. However, it should be emphasized that the determined *K*_d_ values represent apparent binding affinities, as the cell binding over 60 min covers both binding to nectin-4 and putative internalization. Thus, the *B*_max_ values measured with intact cells do not represent the amount of cellular nectin-4. For assessing the binding affinity exclusively to cellular nectin-4, saturation binding curves after washing the cells with acidic glycine buffer would be more suitable to record surface-bound radioligand, which, however, was not performed herein. Overall, the different binding affinities of the radioligands obtained by saturation binding analysis rationalize the large differences in specific cell binding observed at 10 nM. In particular, the high *K*_d_ value of 222 nM obtained for **[** ^**68**^ **Ga]­Ga-8d** (**[** ^**68**^ **Ga]­Ga–N188**) might explain why we were not able to detect nectin-4-specific binding at a radioligand concentration of 10 nM.

### Radiopharmacological Characterization In Vivo and Ex Vivo

The biodistribution of the ^64^Cu- and ^68^Ga-labeled nectin-4-ligands was assessed in a subcutaneous HT-1376 tumor xenograft model via small-animal PET imaging (Figure 8A for ^64^Cu-labeled ligands and Figure 9A for ^68^Ga-labeled ligands). The time-activity curves (TACs) for tumor and heart are shown in Figures 8B,C and 9B,C (TACs for muscle, kidney, liver, and urinary bladder are provided in the Supporting Information, Figure S15).

**Figure 8:**
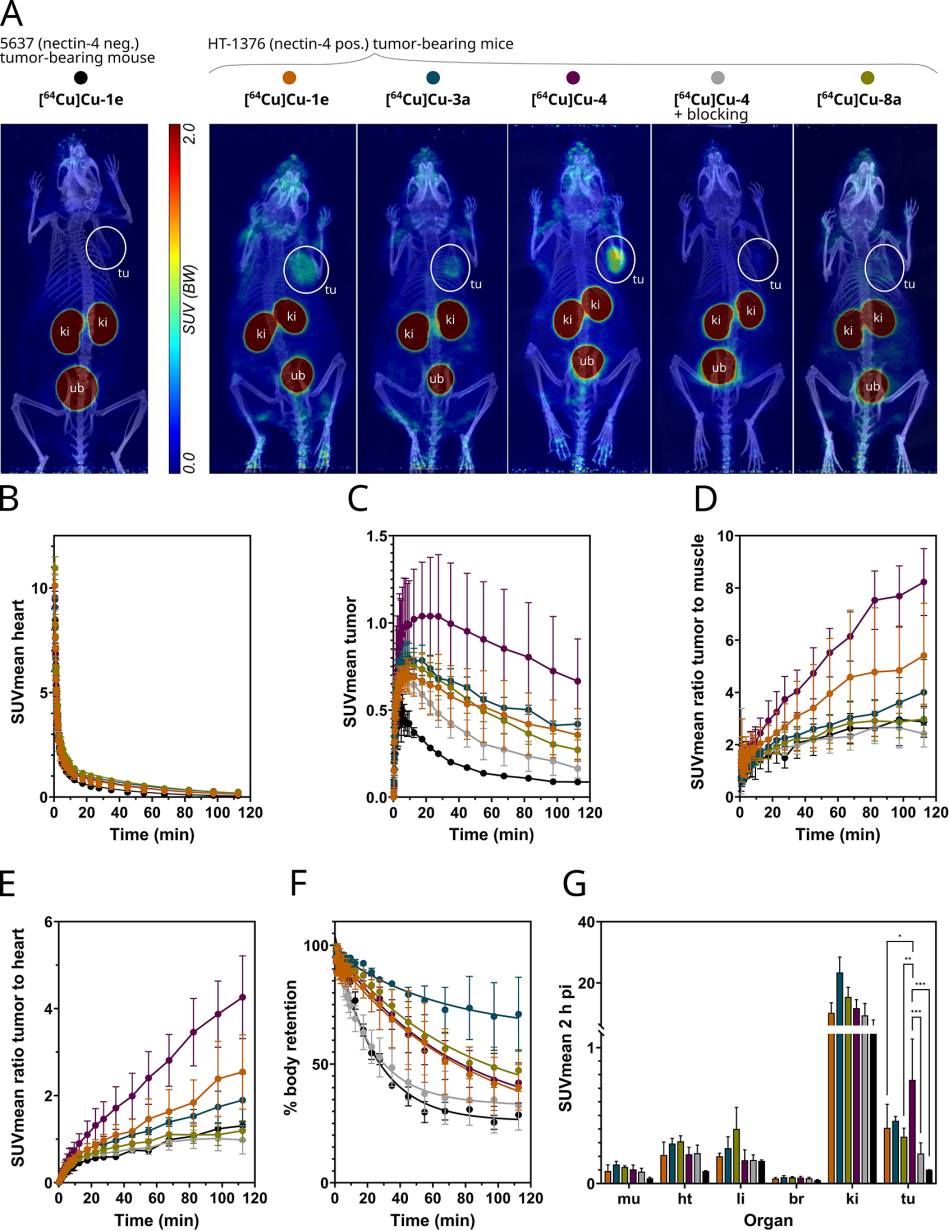
PET images and image-derived uptake values of the ^64^Cu-labeled nectin-4 ligands. (A) PET/CT images at 1–2 h after intravenous injection of **[^64^Cu]­Cu-1e**, **[^64^Cu]­Cu-3a**, **[^64^Cu]­Cu-4**, and **[^64^Cu]­Cu-8a**, and of **[^64^Cu]­Cu-4** coinjected with 75 nmol of 1d (blocking) in HT-1376 tumor-bearing mice and of **[^64^Cu]­Cu-1e** in 5637 tumor-bearing mice. 7–11 MBq/animal (0.3–0.5 nmol/animal) were injected. Images are presented as maximum intensity projections with a common scale. Anatomical positions of the tumor (tu), kidney (ki), and urinary bladder (ub) are shown. (B,C) Time-activity curves (SUVmean, decay-corrected, as a function of time up to 2 h) for the blood content of the heart (B) and tumor (C) obtained from quantitative analysis of PET images are depicted. (D,E) Time-resolved SUVmean ratios up to 2 h p.i., including tumor-to-muscle (D) and tumor-to-heart (E). (F) Time-resolved body retention in % of the initial dose up to 2 h p.i. G) Biodistribution at 1–2 h p.i., obtained from quantitative analysis of PET images (br,brain; ht, heart; it, intestine; li, liver; mu, muscle; tu, tumor; ub, urinarybladder; ki, kidney). Data points in (B–G) are mean values (±SD) measured in groups of HT-1376 tumor-bearing mice (*n* = 4 or *n* = 6 for **[^64^Cu]­Cu-4**). For (F), an ordinary one-way ANOVA was conducted to statistically compare the mean values of **[^64^Cu]­Cu-4** with the means of all other compounds using the idák correction model. *p ≤ 0.05, **p ≤ 0.01, ***p ≤ 0.001.

**Figure 9:**
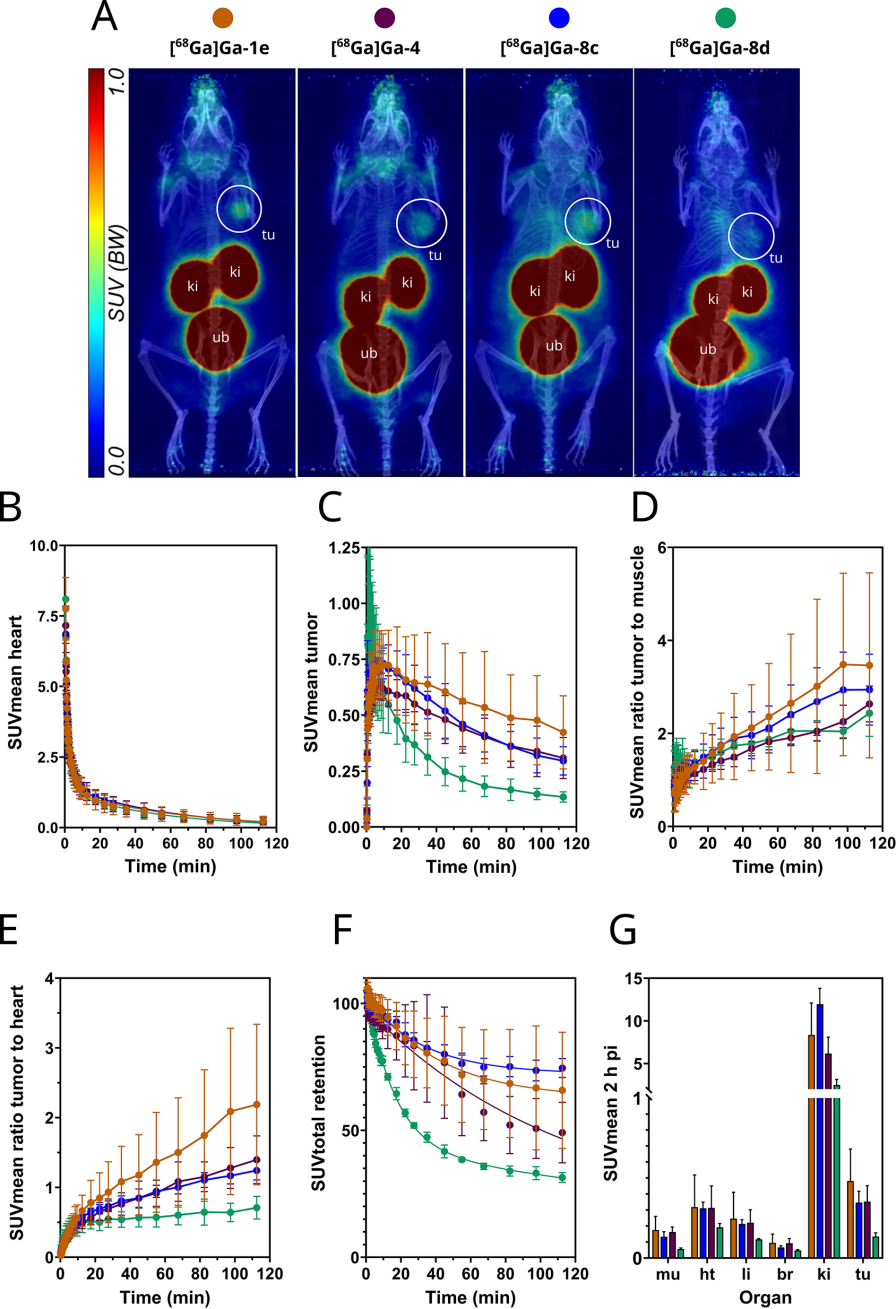
PET images and derived data for the ^68^Ga-labeled peptides. A) PET/CT images at 1–2 h after intravenous injection of **[^68^Ga]­Ga-1e**, **[^68^Ga]­Ga-4**, **[^68^Ga]­Ga-8c**, and **[^68^Ga]­Ga-8d** (**[^68^Ga]­Ga-N188**, 7–9 MBq/animal; 0.5–1 nmol/animal) in HT-1376 tumor-bearing mice. Images are presented as maximum intensity projections with a common scale. Anatomical positions of tumor (tu), kidney (ki), and urinary bladder (ub) are shown. (B,C) Time-activity curves (SUVmean, decay-corrected, as a function of time up to 2 h) for blood content of the heart (B) and tumor (C) obtained from quantitative analysis of PET images are depicted. (D,E) Time-resolved SUVmean ratios up to 2 h p.i. including tumor-to-muscle (D) and tumor-to-heart (E). (F) Time-resolved body retention in % of the initial dose up to 2 h p.i. (G) Biodistribution at 1–2 h p.i. obtained from quantitative analysis of PET images (br,brain; ht, heart; it, intestine; li, liver; mu, muscle; tu, tumor; ub, urinarybladder; ki, kidney). Data points in B-G are mean values (±SD) measured in groups of HT-1376 tumor-bearing mice (*n* = 3).

#### PET Imaging of ^64^Cu-Labeled Ligands

All ^64^Cu-labeled peptides enabled visualization of the nectin-4-positive HT-1376 tumor. In this context, the presence of nectin-4 in HT-1376 and 5637 tumors was checked by immunohistochemical (IHC) staining of tumor sections (Figure 6E) and by quantifying nectin-4 in tumor samples using ELISA (Figure 6D). It is worth noting that the nectin-4 amount per milligram of protein is significantly increased in tumor samples compared to cells cultured as monolayers for both HT-1376 and 5637. In particular, while 5637 cells can be considered nectin-4-negative, there is obviously a considerable nectin-4 level in the tumor xenograft model derived thereof. Therefore, 5637 tumors should be classified as tumors with a low nectin-4 level based on the comparison to HT-1376 tumors. In line with the lower nectin-4 level, radioligand uptake in 5637 tumors was significantly lower compared to HT-1376 tumors, as exemplarily investigated for **[** ^**64**^ **Cu]­Cu-1e** (Figure 8). The nectin-4-specificity of the radioligands was furthermore supported by blocking studies with excess **1d** (75 nmol, 150-fold molar excess, coinjected with the radioligand, for **[** ^**64**^ **Cu]­Cu-4** see Figure 8, for **[** ^**64**^ **Cu]­Cu-1e** and **[** ^**64**^ **Cu]­Cu-3a** see Figure S16).

The radioligands **[** ^**64**^ **Cu]­Cu-1e**, **[** ^**64**^ **Cu]­Cu-3a**, and **[** ^**64**^ **Cu]­Cu-8a** showed the highest tumor uptake at ≈6 min *p.i.,* reaching SUVmean values between 0.7 and 0.8, while **[** ^**64**^ **Cu]­Cu-4** showed a higher tumor uptake, reaching the highest SUVmean value of 1.0 after 12.5 min (Figure 8B). All ^64^Cu-labeled ligands underlay a rapid washout from the tumors, with SUVmean values decreasing to 0.2–0.4 for **[** ^**64**^ **Cu]­Cu-1e**, **[** ^**64**^ **Cu]­Cu-3a**, and **[** ^**64**^ **Cu]­Cu-8a** and 0.7 for **[** ^**64**^ **Cu]­Cu-4** within 2 h *p.i*. All ^64^Cu-labeled ligands exhibited similar off-target uptake in normal organs and were predominantly excreted via the renal pathway. The TACs for the heart (blood content only) followed biphasic blood kinetics with a radioligand fraction of ≈85% being removed during the first (distribution) phase with half-lives of 1.2–1.3 min, followed by a second (elimination) phase proceeding more slowly with half-lives of 32–38 min (Figure 8B and Table S4). At 2 h *p.i.*, 40–42% of the initial **[** ^**64**^ **Cu]­Cu-1e**, **[** ^**64**^ **Cu]­Cu-4**, and **[** ^**64**^ **Cu]­Cu-8a** activity doses were still retained in the body (Figure 8F), while for **[** ^**64**^ **Cu]­Cu-3a,** a significantly higher proportion of 70% was retained. In line with this, the kidney uptake of **[** ^**64**^ **Cu]­Cu-3a** at 2 h *p.i.* was higher compared to the other radioligands (SUVmean values of 25 vs 10–13, respectively, Figure 8G).

Owing to its higher tumor uptake, the tumor-to-muscle, tumor-to-heart, tumor-to-kidney, and tumor-to-liver ratios were most favorable for **[** ^**64**^ **Cu]­Cu-4**, followed by **[** ^**64**^ **Cu]­Cu-1e** and **[** ^**64**^ **Cu]­Cu-3a** (Figure 8D,E, Table 4). **[** ^**64**^ **Cu]­Cu-8a** showed the lowest tumor-to-tissue ratios, which were in a similar range as those occurring from nonspecific uptake of **[** ^**64**^ **Cu]­Cu-1e** in the presence of coinjected **1d** (Table 4). Consequently, methoxinine in position 4 of the bicyclic scaffold of **[** ^**64**^ **Cu]­Cu-4** clearly outperforms methionine (**[** ^**64**^ **Cu]­Cu-1e**) and norleucine (**[** ^**64**^ **Cu]­Cu-3a**) regarding the in vivo performance, albeit no differences were initially observed in vitro for binding to recombinant human nectin-4 (including *k*_on_ and *k*_off_ values) as well as for binding to HT-1376 cells. Thus, the reasons for the better in vivo performance of **[** ^**64**^ **Cu]­Cu-4** might be related to unknown factors that affect the pharmacokinetic properties rather than the actual binding properties to nectin-4. This illustrates that not only subtle structural changes substantially affect the target binding properties but also the pharmacokinetic properties.^63^

**Table 4:** SUVmean and SUVmean Ratios at 1–2 h *p.i.* for the ^64^Cu-Labeled Peptides in a HT-1376-derived Tumor Xenograft Model^a^

	[^64^Cu]Cu-1e	[^64^Cu]Cu-3a	[^64^Cu]Cu-4	[^64^Cu]Cu-8a	[^64^Cu]Cu-4 + blocking*	[^64^Cu]Cu-1e 5637 tumor
tumor SUVmean_1–2h p.i._	0.41 ± 0.15	0.46 ± 0.03	0.76 ± 0.27	0.34 ± 0.05	0.22 ± 0.07	0.10 ± 0.00
SUVmean ratios (1–2 h p.i.)						
tumor to muscle	4.90 ± 1.86	3.46 ± 0.69	7.40 ± 0.94	2.89 ± 0.56	2.51 ± 0.51	2.77 ± 0.44
tumor to heart	2.10 ± 0.59	1.64 ± 0.25	3.60 ± 0.69	1.13 ± 0.19	0.98 ± 0.21	1.15 ± 0.02
tumor to liver	2.07 ± 0.71	1.97 ± 0.67	4.71 ± 1.04	0.92 ± 0.22	1.25 ± 0.27	0.61 ± 0.02
tumor to kidney	0.04 ± 0.01	0.02 ± 0.01	0.07 ± 0.03	0.02 ± 0.01	0.03 ± 0.01	0.02 ± 0.00

a Data shown are mean values (±SD). * Co-injection of 75 nmol of **1d**.

PET imaging at 24 h *p.i.* was exemplarily performed for **[** ^**64**^ **Cu]­Cu-1e** with and without coinjection of **1d** (Figure S17). The SUVmean values of tumors were similarly low (<0.1) under both conditions, indicating that detection of tumors with **[** ^**64**^ **Cu]­Cu-1e** at such late time points is not feasible in HT-1376 tumor-bearing mice. Furthermore, in view of a potential application of the radioligands in humans, we exemplarily studied the biodistribution of **[** ^**64**^ **Cu]­Cu-4** and **[** ^**64**^ **Cu]­Cu-8a** in HT-1376 bearing mice after intravenous injection of a total radioligand amount of only 0.01 nmol (Figure S18), which is by a factor of 40 lower than the amounts usually applied for preclinical imaging studies. For this purpose, ^64^Cu-labeling of **4** and **8a** was performed at an apparent molar activity of 200 MBq/nmol (common molar activities were ≈25 MBq/nmol), which also resulted in radiochemical yields >98% owing to the high (radio)­chemical purity of our in-house produced [^64^Cu]­CuCl_2_.^50,64^ It is worth noting that the shape and height of the tumor TAC were comparable between the two radioligand doses. Accordingly, the more favorable in vivo performance of **[** ^**64**^ **Cu]­Cu-4** compared to **[** ^**64**^ **Cu]­Cu-8a** was conserved over a broad range of radioligand amount. To shed light on the potential implications of the partial oxidation of **[** ^**64**^ **Cu]­Cu-1e** during ^64^Cu-labeling for PET imaging, the biodistribution of the authentic oxidation product, **[** ^**64**^ **Cu]­Cu-2**, which is still a potent nectin-4 ligand (Table 2), was assessed by PET imaging (Figure S19). While **[** ^**64**^ **Cu]­Cu-2** reached higher SUVmean values in tumors, its washout was faster compared to that of **[** ^**64**^ **Cu]­Cu-1e**. Furthermore, **[** ^**64**^ **Cu]­Cu-1e** showed higher tumor-to-organ ratios and thus an overall slightly better in vivo performance compared to its methionine sulfoxide analog **[** ^**64**^ **Cu]­Cu-2**.

#### PET Imaging of ^68^Ga-Labeled Ligands

PET imaging of the ^68^Ga-labeled nectin-4 ligand versions (**[** ^**68**^ **Ga]­Ga-1e**, **[** ^**68**^ **Ga]­Ga-4**, and **[** ^**68**^ **Ga]­Ga-8c**) showed that their overall biodistribution resembles that of the corresponding ^64^Cu-labeled ligands (Figure 9). The TAC for the blood content of the heart is also biphasic with half-lives of 1.4–1.5 and 37–42 min for the fast (distribution) and slow (elimination) phases, respectively (Figure 9B and Table S5). The ^68^Ga-labeled ligands were also predominantly excreted via the renal pathway. However, the extent of body retention was higher for the ^68^Ga-labeled ligands compared to their ^64^Cu-labeled counterparts (e.g., initial doses of 65% for **[** ^**68**^ **Ga]­Ga-1e** and 40% for **[** ^**64**^ **Cu]­Cu-1e** at 2 h *p.i.*). **[** ^**68**^ **Ga]­Ga-8c** showed the highest retained fraction with 75% of the initial dose still present at 2 h *p.i.* (Figure 9F), which is in line with the result that this radioligand exhibited the highest uptake in the kidneys at this time point (Figure 9G). The three ^68^Ga-labeled ligands showed a nectin-4-specific uptake in HT-1376 tumors (Figure 9A,C) with similar TAC profiles as obtained for their ^64^Cu-labeled counterparts. The highest tumor uptake was reached after 8.5 min *p.i.,* and SUVmean at this time point was similar for **[** ^**68**^ **Ga]­Ga-1e**, **[** ^**68**^ **Ga]­Ga-8c**, and **[** ^**68**^ **Ga]­Ga-4** (0.64–0.72). It is worth noting that although **[** ^**68**^ **Ga]­Ga-8c** reached an initially higher SUVmean compared to **[** ^**68**^ **Ga]­Ga-4**, its washout from the tumors tended to be faster, finally approaching a similar SUVmean after 1–2 h p.i. (both 0.35). This is consistent with the trend observed for the ^64^Cu-labeled ligands, where [^**64**^ **Cu]­Cu-8a** also showed the fastest washout from the tumors. In this context, compounds **8a** and **8c** share the same bicyclic scaffold (2NaI^2^/Met^4^/Arg^5^). Regarding the tumor-to-organ ratios, **[** ^**68**^ **Ga]­Ga-1e** showed the most favorable properties (Figure 9D,E and Table 5), mainly due to its longer tumor retention compared to the other two radioligands.

**Table 5:** SUVmean and SUVmean Ratios at 1–2 h p.i. for the ^68^Ga-Labeled Peptides in a HT-1376-derived Tumor Xenograft Model^a^

	[^68^Ga]Ga-1e	[^68^Ga]Ga-4	[^68^Ga]Ga-8c	[^68^Ga]Ga-8d
tumor SUVmean _1–2h_	0.48 ± 0.16	0.35 ± 0.06	0.35 ± 0.08	0.16 ± 0.03
SUVmean ratios (1–2 h p.i.)				
tumor-to-muscle	3.15 ± 1.49	2.74 ± 0.56	2.21 ± 0.21	2.15 ± 0.20
tumor-to-heart	1.88 ± 0.83	1.13 ± 0.09	1.23 ± 0.23	0.65 ± 0.13
tumor-to-liver	2.46 ± 1.13	1.64 ± 0.15	1.67 ± 0.19	1.04 ± 0.21
tumor-to-kidney	0.07 ± 0.03	0.03 ± 0.01	0.06 ± 0.01	0.06 ± 0.02

a Data shown are mean values (±SD).

In contrast to the aforementioned ^68^Ga-labeled ligands, **[** ^**68**^ **Ga]­Ga-8d** (**[** ^**68**^ **Ga]­Ga–N188**) showed a distinctly different biodistribution (Figure 9). The body retention (35% at 2 h *p.i.*) was significantly lower compared to the other ^68^Ga-labeled ligands. Furthermore, the highest tumor uptake (SUVmean of ≈1.0) was reached already at 0.6 min *p.i.*, which was largely maintained up to 3–4 min, followed by a rapid washout from the tumor tissue with a SUVmean of 0.16 at 2 h *p.i.* It is worth mentioning that although the tumor uptake is low at 2 h *p.i.*, the tumor-to-muscle ratio is comparable to those of **[** ^**68**^ **Ga]­Ga-4** and **[** ^**68**^ **Ga]­Ga-8a**. Comparing the results for **[** ^**68**^ **Ga]­Ga-8c** and **[** ^**68**^ **Ga]­Ga-8d**, it is striking that a minimal structural modification (−CONH_2_ versus COOH) in relation to the total size of the molecule can exert profound differences in target binding and overall biodistribution.

#### Ex Vivo Metabolite Analysis for [^64^Cu]­Cu-4

In addition to the biodistribution, we were interested in studying the metabolic fate of the radiolabeled bicyclic peptides in vivo. An ex vivo metabolite analysis (blood, liver, kidney, and urine) was performed for **[** ^**64**^ **Cu]­Cu-4** at different time points (10, 30, and 90 min) after its intravenous injection in healthy mice (Figure 10). Accordingly, the blood analysis revealed that at 10 min *p.i.* 70% of **[** ^**64**^ **Cu]­Cu-4** remained intact, while at 90 min *p.i.*, only 30% of the original radioligand was still detectable. These results are in contrast to the stability of **[** ^**64**^ **Cu]­Cu-4** in human plasma in vitro, where basically no sign of any metabolic transformation has been observed over 24 h (Figure S10). Although species differences should be considered, it appears more likely that the in vitro incubations in blood plasma are of limited significance for the potential in vivo stability in the blood circulation. In vitro incubations in blood plasma can capture only the activity of soluble proteases but not the activity of endothelium-derived proteases. Similar discrepancies between in vitro and in vivo half-lives of radiolabeled peptides were previously reported.^65,66^ While **[** ^**64**^ **Cu]­Cu-4** was still the main radiolabeled species at 10 min *p.i.* in the blood circulation, only around 24% were present in the liver, and only a negligible residual fraction of intact radioligand was detectable in the kidneys and urine. Consequently, the data suggest that **[** ^**64**^ **Cu]­Cu-4** is rapidly metabolized during passaging through the kidneys, even more rapidly than in blood circulation. Metabolism in the liver might also occur; however, considering the overall low liver uptake, this might be of lower importance. It is worth noting that at 90 min *p.i.,* only one radiolabeled metabolite was detectable in the kidneys. Based on the ex vivo metabolite analysis, we conclude that metabolization in the blood circulation might not significantly affect the tumor targeting capability, in particular as the bicyclic peptides exhibit anyway a fast blood clearance.

**Figure 10:**
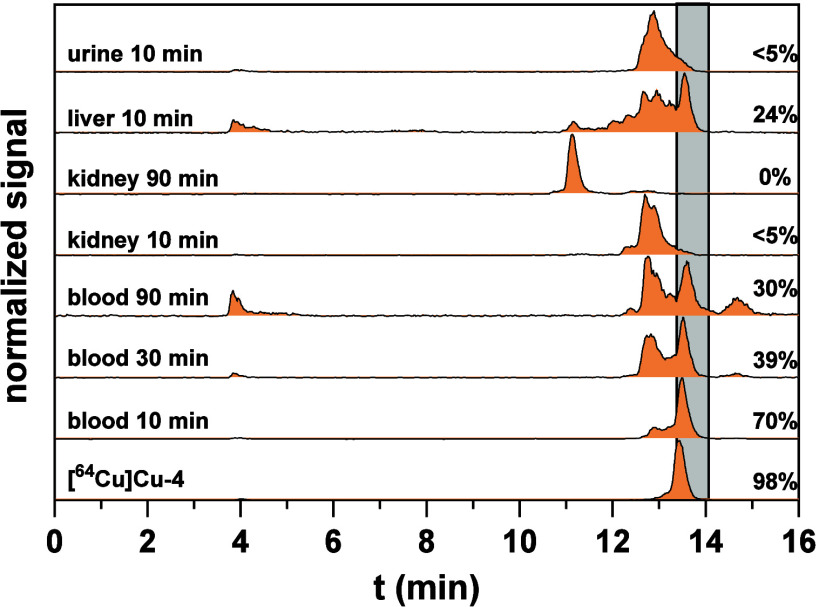
Ex vivo metabolite analysis for **[^64^Cu]­Cu-4**. Radio-HPLC chromatograms of **[^64^Cu]­Cu-4** (*t*_R_ = 13.4 min) and for samples of blood, kidney, liver, and urine were taken at different time points after i.v. injection of **[^64^Cu]­Cu-4** in healthy NMRI-nu/nu mice. Residual intact radioligand based on integration is given for each HPLC trace.

#### Considerations Regarding the Observed Tumor Uptake of the Radiolabeled Bicyclic Peptides Targeting Nectin-4

Reviewing the PET imaging data for all radioligands, it becomes clear that despite a high cell binding to HT-1376 cells in vitro, which is comparable to SST_2_ or PSMA binding to cancer cells used for preclinical studies (based on *B*_max_ values),^67,68^ the integral tumor uptake and in particular the tumor residence time is comparatively low. Considering the observed pharmacokinetics, the comparatively low tumor uptake of the nectin-4 targeting bicyclic peptides could result from their rapid blood clearance. In this context, comparable or even faster blood clearance (distribution and elimination half-lives) were reported for other radiolabeled bicyclic peptides targeting nectin-4,^38,52^ but also for bicyclic peptides targeting uPA,^69^ EphA2,^20^ and MT1-MMP.^16^ However, a fast blood clearance is a common feature of peptides and also of radiolabeled peptides that show, however, a high and long-lasting tumor uptake (e.g., carbonic anhydrase IX ligand DPI-4452 ^70^).

From the perspective of the nectin-4–radioligand interaction, the ligands characterized for their in vivo performance upon radiolabeling exhibit widely differing equilibrium dissociation constants (e.g., 127 nM for **8c** and 3.2 nM for **4** as determined by SPR). However, the macroscopic rate constants *k*_on_ and *k*_off_ do not follow the trend for the *K*_d_ values (e.g., *k*_off_ values of 2.39 × 10^–3^ s^–1^ and 7.59 × 10^–3^ s^–1^ for **8c** and **4**, respectively). In contrast to the static conditions, including an invariant radioligand concentration that are present during in vitro experiments, such as cell binding herein (closed system), the conditions are highly dynamic in vivo, with the radioligand concentration fluctuating (open system). Accordingly, Robert A. Copeland and colleagues proposed that for classic drug–target interactions, the drug-target residence (1/*k*_off_) is better suited for characterizing the duration of efficacy of a drug in vivo,^71^ which might be translated to the height and duration of tumor uptake for radioligands. This model has gained broad acceptance for drug optimization campaigns, as supported by several studies showing that indeed the in vivo efficacy often correlates with the drug-target residence time.^72^ Consequently, we hypothesize that the comparable *k*_off_ values of **1e**, **4**, and **8c** account for the comparable in vivo performance by means of tumor uptake of their ^68^Ga-labeled analogs. Moreover, the rapid tumor washout observed for all radioligands could also be rationalized on the basis of the *k*_off_ values as radioligands with a long-lasting tumor uptake exhibit *k*_off_ values that are at least 1 order of magnitude lower than for the ligands herein (*k*_off_ values <0.3 × 10^–3^ s^–1^ were reported for radioligands targeting the somatostatin receptor subtype 2, carbonic anhydrase IX or glypican-3).^70,73–75^ The fast dissociation rate might also limit the extent of potential radioligand internalization in vivo. In this context, the development of nectin-4-targeted miniproteins by Aktis Oncology already aimed at lowering *k*_off_ with the lead compound **AKY-1189** exhibiting a *k*_off_ of 1 × 10^–3^ s^–1^.^76^ Consequently, prospective structural optimizations of the bicyclic peptides based on **BT8009** should include the determination of *k*_on_ and *k*_off_ values to potentially enable a higher tumor uptake and slower washout, which would also open up opportunities for targeted endoradionuclide therapy as recently initiated for the miniprotein **[** ^**225**^ **Ac]­Ac-AKY-1189** within the scope of a clinical trial (NCT07020117). In this context, a previous report on the structure-guided optimization of bicyclic ACE2 inhibitors demonstrated that also *k*_off_ values <10^–3^ s^–3^ can be achieved for this class of molecules.^21^

### First-in-Human Application of **[^68^Ga]­Ga-4** (**[^68^Ga]­Ga-NECT-224**)

According to our preclinical studies, compound **[** ^**64**^ **Cu]­Cu-4** (**[** ^**64**^ **Cu]­Cu-NECT-224**), showed the most favorable properties among all ^64^Cu- and ^68^Ga-labeled ligands in terms of radiochemical purity, binding affinity to nectin-4, as well as tumor uptake, and tumor-to-tissue ratios. Encouraged by these results, a first-in-human application of its ^68^Ga-labeled analog, **[** ^**68**^ **Ga]­Ga-NECT-224**, was initiated, as the radionuclide ^64^Cu is not commonly applied for PET imaging in humans.

A 59-year-old woman underwent after informed consent and as part of an individual diagnostic concept, which was recommended by the interdisciplinary tumor conference of the Comprehensive Cancer Center, **[** ^**68**^ **Ga]­Ga-NECT-224** PET/CT for restaging of metastatic urothelial carcinoma originating from the left kidney, diagnosed 10 months earlier. Before imaging, the patient had received four cycles of immunochemotherapy (cisplatin, gemcitabine, nivolumab) and subsequently progressed during maintenance therapy with nivolumab alone. The PET scan, as shown in the Maximum Intensity Projection (MIP, Figure 11A), demonstrates multiple nectin-4–expressing tumor lesions in the brain, renal pelvis, lymph nodes, and bones alongside the physiological tracer distribution. The most prominent uptake of **[** ^**68**^ **Ga]­Ga-NECT-224** is seen in three brain metastases with moderate SUVmax values ranging from 7.1 to 8.3, detailed in the axial fused PET/CT (Figure 11B) and confirmed by consecutively performed cerebral MRI (contrast-enhanced T2, Figure 11C). The primary tumor in the left renal pelvis exhibits high uptake of **[** ^**68**^ **Ga]­Ga-NECT-224** (SUVmax: 23.8) and is clearly distinguishable from the surrounding renal parenchyma (Figure 11D). Stereotactic radiotherapy for the brain metastases was initiated, and systemic therapy with **enfortumab vedotin** is currently under consideration for this patient.

**Figure 11:**
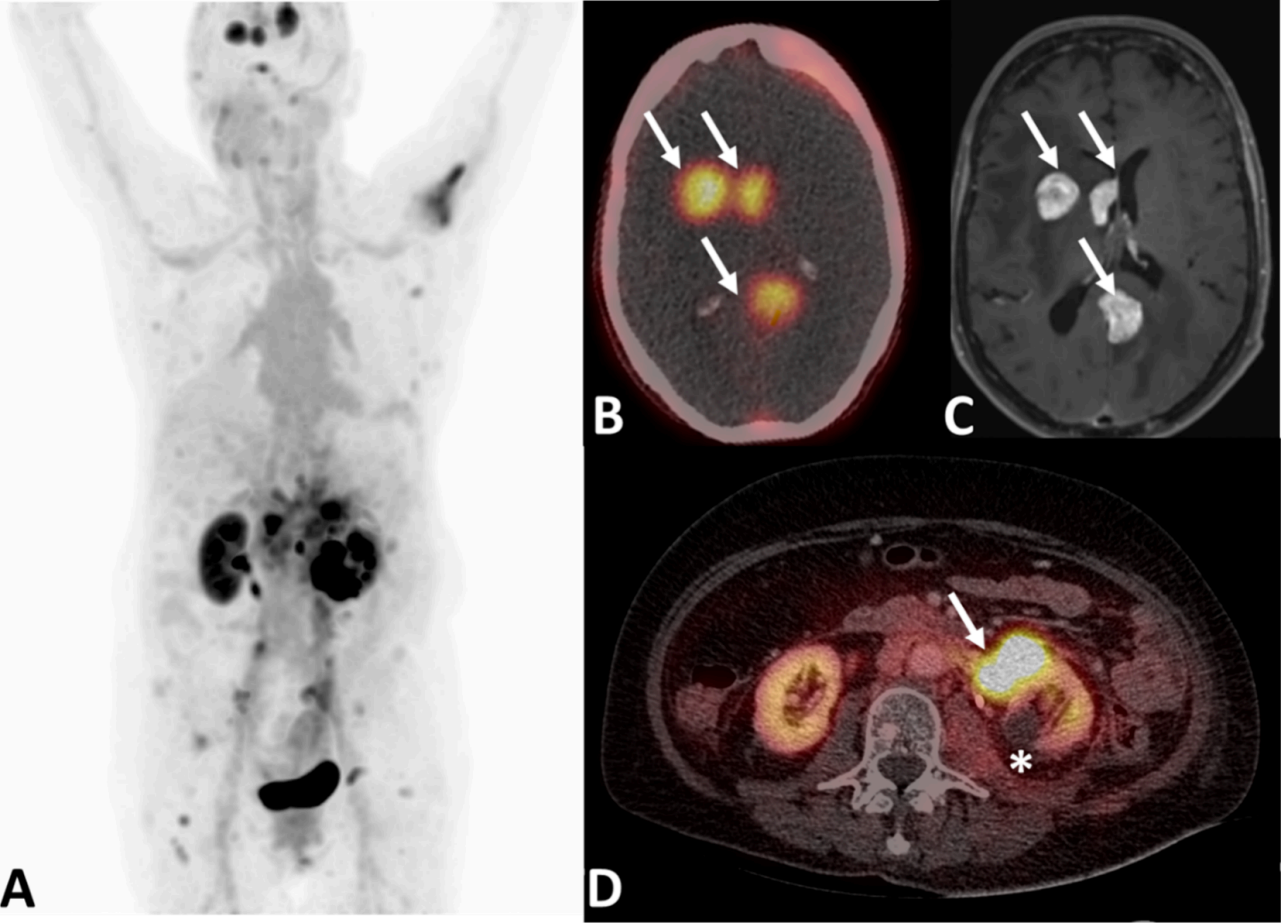
First-in-human application of **[^68^Ga]­Ga-4** (**[^68^Ga]­Ga-NECT-224**). (A) Maximum intensity projection (MIP) scaled to a maximum standardized uptake value (SUVmax) of 8, demonstrating physiological distribution of **[^68^Ga]­Ga-NECT-224** alongside multiple tumor manifestations (imaging 41 min p.i., 149 MBq were injected). Physiological accumulation of **[^68^Ga]­Ga-NECT-224** is particularly notable in the pituitary gland, renal pelvis, and urinary bladder. Modest vascular tracer retention and only faint uptake are observed in the liver and salivary glands. The most prominent metastases, together with the primary tumor located in the pelvis of the left kidney, are seen in the brain (three lesions), abdominal lymph nodes, and the left humerus. (B) Axial fused PET/CT showing clearly visible uptake of **[^68^Ga]­Ga-NECT-224** in three cerebral metastases, with near-photopenic background activity. (C) Consecutive MRI (T1 SPACE axial sequence) confirming two right-sided lenticulostriatal and one left precuneal cerebral metastasis. (D) Axial fused PET/CT image scaled to an SUVmax of 15. The primary tumor in the pelvis of the left kidney (arrow), along with a locoregional lymph node metastasis, is clearly distinguishable from the adjacent renal parenchyma. A small cyst in the posterior aspect of the left kidney (asterisk) shows no radiotracer uptake.

This first-in-human application of **[** ^**68**^ **Ga]­Ga-NECT-224** supports its further clinical evaluation. Labeling with radionuclides with longer half-lives, such as ^64^Cu, may allow imaging at later time points with potentially improved target-to-background ratios. In this context, the preclinically observed in vivo performance, with even superior tumor uptake and tumor-to-tissue ratios for **[** ^**64**^ **Cu]­Cu-NECT-224** compared to those for **[** ^**68**^ **Ga]­Ga-NECT-224**, is encouraging.

## Conclusion

The present study aimed at translating the nectin-4-directed bicyclic toxin conjugate **BT8009** into radiolabeled bicyclic peptides for noninvasive tumor imaging with positron emission tomography. In the course of refining the parent bicyclic scaffold, a major focus lay on the bioisosteric replacement of the original methionine residue in position 4 due to its susceptibility to oxidation during synthesis and radiolabeling experiments. The small library of novel peptides was characterized regarding not only their equilibrium dissociation constants but also the macroscopic rate constants for association and dissociation, which revealed interesting SARs. In fact, we discovered discrepancies between the trends in *K*_d_ and *k*_off_, with a large impact being exerted by the amino acid in position 2 in combination with the identity of the N-terminal chelator. Selected peptides were radiopharmacologically characterized in vitro and in vivo upon ^64^Cu- or ^68^Ga-labeling. Radiolabeling of the peptides bearing methoxinine, norleucine, *O*-ethylserine, or *S*-ethylcysteine in position 4 provided a higher radiochemical purity of the respective radioligands compared with the radioligands bearing methionine. In the course of the cell binding studies, we discovered the phenomenon of a pronounced internalization of the radioligands into urothelial carcinoma cells (HT-1376). Though this internalization depends on nectin-4, the protein itself remains at the cell surface. Further studies are needed to shed more light on this aspect. All studied radioligands enabled a nectin-4 specific tumor uptake, with the best performance in terms of tumor uptake and tumor-to-tissue ratios being obtained for **[** ^**64**^ **Cu]­Cu-4**, also named **[** ^**64**^ **Cu]­Cu-NECT-224**. Accordingly, methoxinine turned out to be the methionine bioisostere with the most favorable properties for in vivo application. The suitability of **NECT-224** for imaging purposes was also demonstrated in a first-in-human application of **[** ^**68**^ **Ga]­Ga-NECT-224**. The results suggest its further clinical development, which will form the basis for the clinical evaluation of **[** ^**64**^ **Cu]­Cu-NECT-224** in the next step, enabling image acquisition at later time points for potentially improved tumor-to-background ratios. Ongoing preclinical studies are focused on structural modifications that lower the dissociation rate constant of the bicyclic peptides, which should improve the tumor uptake and thus enable a prolonged tumor retention to expand the theranostic opportunities for the bicyclic nectin-4 ligands.

## Experimental Section

### General

All commercial reagents and solvents were used without further purification, unless otherwise specified. The purity of the bicyclic peptides **1–4** and **6–10** proved to be ≥95% as analyzed by analytical RP-HPLC. The purity of compound **5**, which bears selenomethionine, was lower due to the lability to oxidation (as discussed above). The HPLC-based determination of chromatographic hydrophobicity indices at an immobilized artificial membrane was performed as previously described^77^ according to the method developed by Valko et al.^78^

### Chromatography

The HPLC system used was a LC-20A Prominence HPLC by Shimadzu, consisting of a degasser unit DGU-20A5R, two separate pumping units LC-A20R, a sample manager SIC-20ACHT, column oven CTO-20AC, PDA-detector SPD-M20A, communication-bus module CBM-20A, and fraction collector FRC-10A. Two Aeris Peptide 5 μm XB-C18 columns (100 Å, 250 × 4.6 mm and 250 × 21.2 mm) were used as the stationary phases for analytical and preparative RP-HPLC, respectively. A binary gradient system of 0.1% CF_3_COOH/water (solvent A) and 0.1% CF_3_COOH/CH_3_CN (solvent B) at a flow rate of 1 mL/min (analytical) or 10 mL/min (preparative) served as the eluent. For determining the purity of the bicyclic peptides **1**–**10** with analytical RP-HPLC, the following gradient was applied: 25% eluent B for 5 min, 25–75% eluent B in 25 min, 75–95% eluent B in 1 min, 95% eluent B for 5 min, 95–25% eluent B in 1 min, and 25% eluent B for 5 min. High-resolution mass spectra (HRMS) of compounds **1**-**9** were obtained on an Orbitrap mass spectrometer using electrospray ionization: a syringe pump coupled to a Thermo Scientific Orbitrap Exploris 120. The measurements were performed in direct injection mode using an eluent consisting of: 0.1% formic acid in MeOH/Water (50:50 v/v); flow rate 3 μL/min. High resolution mass spectrum of compound **10** was obtained on a Q-TOF MS using electrospray ionization: Agilent 1260 Infinity II HPLC (Santa Clara, California, USA; pump G7104C, autosampler G7129C, column oven G7116A, DAD detector G7117C) coupled to a γ detector Gabi Star (Raytest Isotopenmeßgeräte GmbH, Straubenhardt, Germany) followed by accurate mass Revident Q-TOF LC/Q-TOF G6575A. The measurements were performed in bypass mode using an eluent consisting of (A): CH_3_CN and (B): 0.1% formic acid in H_2_O; flow rate 0.2 mL/min. A reference mass solution containing hexakis­(1H,1H,3H-tetrafluoropropoxy)­phosphazene and purine was continuously coinjected via dual AJS ESI source. The system was operated using Agilent Masshunter Workstation 3.6 – LC/MS data acquisition software (Version 12.0), and data evaluation was performed using Agilent Masshunter Workstation 3.6 Qualitative Analysis software (Version 12.0 Update 1).

For UPLC-DAD-MS (reaction monitoring), a system from Waters (ACQUITY UPLC I class system, including an ACQUITY UPLC PDA e λ detector coupled to a Xevo TQ-S mass spectrometer) was used. An ACQUITY UPLC BEH C18 column (1.7 μm, 130 Å, 100 × 2.1 mm, equipped with an ACQUITY UPLC BEH C18 VanGuard precolumn, 1.7 μm, 130 Å, 5 × 2.1 mm) was used as the stationary phase. A binary gradient system of 0.1% CH_3_COOH/water (solvent A) and 0.1% CH_3_COOH in CH_3_CN/CH_3_OH (1:1, v/v, solvent B) at a flow rate of 0.4 mL/min served as the eluent.

Analytical radio-HPLC after ^64^Cu-labeling was performed on a Series 1200 device (Agilent Technologies, Santa Clara, CA, USA) equipped with a GABI ß/γ-ray detector (Raytest, Straubenhardt, Germany). Eluent A: 0.1% (v/v) trifluoroacetic acid in H_2_O; eluent B: 0.1% (v/v) trifluoroacetic acid in acetonitrile; HPLC system: Aeris Peptide XB-C18, 100 Å, 5 μm, 250 × 4.6 mm (Phenomenex); gradient elution using 70% eluent A for 2 min, 70% eluent A to 90% eluent B in 12 min, 95% eluent B for 2 min, and 95% eluent B to 70% eluent A in 1 min, 1 mL/min, 50 °C, recovery of activity (decay-corrected) was >95%.

Analytical radio-HPLC after ^68^Ga-labeling was performed on a PerkinElmer Flexar UHPLC system equipped with a Ramona ß/γ-ray detector (Raytest, Straubenhardt, Germany). Eluent A: 0.1% (v/v) trifluoroacetic acid in H_2_O; eluent B: 0.1% (v/v) trifluoroacetic acid in acetonitrile; HPLC system: Kinetex XB-C18, 100 Å, 2.6 μm, 100 × 2.1 mm (Phenomenex); gradient elution using 95% eluent A for 1 min, 95% eluent A to 95% eluent B in 5 min, 95% eluent B for 2 min and 95% eluent B to 95% eluent A in 1 min and 5% eluent B for 3 min, 0.5 mL/min, recovery of activity (decay-corrected) was >95%.

### General Solid-Phase Syntheses of Peptides

All peptides were synthesized by automated microwave-assisted solid-phase peptide synthesis (SPPS) using a Biotage Initiator+ Alstra with standard protocols for resin loading, amino acid coupling, and Fmoc removal, which were previously described in detail.^59^ In brief, Fmoc-Rink Amide resin was used as polymeric support, and peptide assembly was performed by repetitive cycles of Fmoc removal (20% piperidine in DMF) and coupling (4 eq. amino acid, 4 eq. HATU, 8 eq. DIPEA, in DMF). N-terminal acylation with *R*-NODAGA-(tBu)_3_ (2 equiv), DOTA-(tBu)_3_ (2 equiv), 6-TAMRA (2 equiv), or 6-FAM (10 equiv) was manually performed using HATU (2 or 10 equiv) and DIPEA (4 or 20 equiv) in DMF. After coupling of 6-FAM, treatment with 20% piperidine in DMF was followed. N-terminal acetylation was performed with acetic anhydride (10 equiv) and DIPEA (10 equiv) in DMF. Cleavage from the resin and concomitant removal of all protecting groups was realized by treatment with TFA/H_2_O/TIPS (95:2.5:2.5, v/v/v, 4 h at 40 °C), and after removal of TFA (with N_2_ flow), the peptides were precipitated with ice-cold diethyl ether. The linear peptides were dissolved in a CH_3_CN/H_2_O mixture (1:1, v/v) and diluted with NH_4_HCO_3_ (100 mM) to a final concentration of ≈1 mM. TCEP (1 equiv) was added to this solution. The cyclization was started by the addition of TATA (1.3 equiv). After completion of the reaction (≈1 h), the pH value was adjusted to 2.0 with TFA/water (1:9, v/v). The solution was then lyophilized, and the crude products were purified by RP-HPLC.

For the synthesis of compound **8d**, loading of Fmoc-Cys­(Trt)–OH onto the 2-ClTrtCl resin was performed as previously described.^79^ The synthesis of compound **2** was performed by dissolving **1e** in an aqueous solution of H_2_O_2_ (100 mM, 0.34%). The reaction was monitored by LC-MS analysis, and the mixture was lyophilized after 5 h of reaction time. The crude product was purified by RP-HPLC.

The nonradioactive metal complexes **[** ^**nat**^ **Cu]­Cu-1e**, **[** ^**nat**^ **Cu]­Cu-3a**, and **[** ^**nat**^ **Ga]­Ga-1e** were prepared under the same conditions as applied for radiolabeling (see below) using CuSO_4_ (1.2 equiv) and GaCl_3_ (1.2 equiv). The reaction mixtures were then lyophilized, and the crude products were purified by RP-HPLC.

### NMR Spectroscopy

Exemplary NMR spectra with a focus on secondary structure features were acquired for **1d**. Therefore, 3.5 mg of **1d** were dissolved in 600 μL DMSO-*d*_6_ (99.96% D) and then transferred into a 5 mm quartz NMR tube. NMR spectra were obtained at various temperatures (25–70 °C) on an Agilent DD2–600 system, operating at 14.1 T, with corresponding ^1^H, ^13^C, and ^15^N resonance frequencies of 599.8, 150.8, and 60.8 MHz, respectively, using a 5 mm oneNMR probe. Chemical shifts are reported in parts per million relative to the residual solvent signal (DMSO-*d*_5_) and liquid ammonia for ^1^H/^13^C and ^15^N, respectively. ^1^H NMR spectra were measured by accumulating up to 256 scans, upon excitation by a π/6 (2.63 μs) pulse, followed by 2 s each of acquisition time and relaxation delay. For suppressing the water and/or the residual solvent signals, the presaturation sequence applied a 2 s (^1^H) or 1 s (2D experiments) selective pulse on the respective resonances. 2D correlation NMR techniques were performed using pulse sequences taking advantage of gradient-selection. In addition, heteronuclear single-quantum coherence (HSQC) and heteronuclear multiple-bond correlation (HMBC), as well as the rotating-frame nuclear Overhauser effect spectroscopy (ROESY) were accomplished using adiabatic pulses. Total correlation spectra (TOCSY) were obtained using a zero-quantum filter and 80 ms mixing time, while the ROESY was acquired using 100 ms of spinlock mixing time. ^1^H,^13^C-HSQC and ^1^H,^13^C-HMBC spectra were acquired with 2048 × 1024 complex points in F_2_ and F_1_, 64 and 88 transitions per F_1_ increment, and a relaxation delay of 1 s, respectively. For polarization transfer, (2 × J)^−1^ delays of 3.42 and 62.5 ms were opted, corresponding to 146 Hz ^1^J­(H,C) in HSQC and 8 Hz ^n^J­(H,C) in HMBC, respectively. Homonuclear correlation spectra were measured using 2048 × 512 complex points in F_2_ and F_1_, 64 (COSY) and 88 (TOCSY and ROESY) transitions per F_1_ increment, and a relaxation delay of 1 s, respectively. The ^1^H,^15^N-HSQC was acquired with 2048 × 512 complex points in F_2_ and F_1_, 232 transitions per F_1_ increment, a (2 × J)^−1^ delays of 5.26 ms (95 Hz^1^J­(H,N)), and a relaxation delay of 1 s.

### Fluorescence Anisotropy-Based Binding Assays

All measurements were conducted at 37 °C over 1200 s (interval of 37 s) using a Cytation 5 multimode microplate reader (BioTek Instruments, Software Gen 5) and black 96-well microplates (BRANDplates with F-bottom wells). Experiments were conducted at an excitation wavelength of 540 nm and an emission wavelength of 620 nm. The FA (*r*) was calculated by Gen 5 software from the measured parallel and perpendicular fluorescence intensities (*I*_∥_ and *I*_⊥_, respectively) according to eq 1 using a *G* factor of 0.87 (preset value).

$$r=\frac{I_{∥}-G\times I_{⊥}}{I_{∥}+2G\times I_{⊥}}$$ 1

All further data analyses were conducted with GraphPad Prism (version 10.4.1, GraphPad Software, San Diego, CA, USA). The assay mixture (100 μL) contained an aqueous solution (99 μL) and DMSO (1%, v/v, 1 μL).

For direct binding of the probes **1a**, **1b**, and **8b**, fixed concentrations of these probes (1 nM) and 11 to 13 concentrations of nectin-4 (R&D Systems, Catalog number 2659-N4, e.g., 0.06–250 nM in case of **1a** prepared as a serial 1:1 dilution) were used (three separate experiments, each performed in duplicate). The corresponding stock solutions of the fluorescent probes (2.5 nM) were prepared in 2.5% DMSO/HEPES buffer (20 mM, 50 mM NaCl, and 0.01% Tween20, pH 7.4), while the nectin-4 stock solutions were prepared in HEPES buffer. HEPES buffer (20 μL) and nectin-4 (40 μL) were added to the wells, and the measurement was started by the addition of the probes (40 μL). The FA values were averaged over the time period of 1200 s, and plots of FA = f­([nectin-4]) were analyzed by nonlinear regression to obtain *K*_d_ values of the probes according to the Morrison equation.^80–82^

$$\mathrm{FA}={\mathrm{FA}}_{[\mathrm{nectin}4]=0}+\left\{({\mathrm{FA}}_{[\mathrm{nectin}4]\to \infty }-{\mathrm{FA}}_{[\mathrm{nectin}4]=0})\{([\mathrm{nectin}4]+[R]+K_{d})-\sqrt{\left([\mathrm{nectin}4]+[R]+K_{d}{)}^{2}-4[\mathrm{nectin}4][R\right]}\}\right\}/\left\{2[R]\right\}$$ 2

where FA is the measured FA value, FA_[nectin4]=0_ is the FA value in the absence of nectin-4 (FA value of unbound probe), FA_[nectin4]→∞_ is the FA value at infinite concentrations of nectin-4 (FA value of probe completely bound to nectin-4), and [*R*] is the concentration of the probe.

For direct binding of probe **1a** to the N-terminal Ig-like V domain of nectin-4 (Acro Biosystems, NE4-H82Ea), the same procedure as described previously was applied.

For the competitive binding assay, fixed concentrations of probe **1a** (1 nM) and recombinant human nectin-4 (20 nM) and 10 concentrations of nonlabeled peptide (e.g., 0.24–500 nM prepared as serial 1:1 dilution) were used (two separate experiments, each performed in duplicate). The corresponding stock solutions of **1a** (2.5 nM) and nectin-4 (50 nM) were prepared in HEPES buffer, while the stock solutions of the nonlabeled peptides were prepared in 5% DMSO/HEPES buffer. nectin-4 (40 μL) and **1a** (40 μL) were added to the wells, and the competition was started by the addition of the nonlabeled peptides (20 μL). The FA values were averaged over the time period of 1200 s, and plots of FA = f­([peptide]) were analyzed by nonlinear regression according to the model “[inhibitor] vs. response – Variable slope (four parameters)” as implemented in GraphPad Prism. The obtained IC_50_ values were then transformed into *K*_d_ values according to the mathematical equation derived by Nikolovska-Coleska et al.^41^ (a *K*_d_ of 0.94 nM for probe **1a** was used, Table 2). For compounds **6** and **9**, the competitive binding curves did not reach the lower FA plateau (i.e., complete displacement of probe **1a** from nectin-4), and the respective FA values were not correctly determined by nonlinear regression. Therefore, the lower FA plateau was constrained to the FA value of probe **1a** in the absence of nectin-4 and the competitor, which was recorded in each experiment. For compounds **1f**, **7**, **8a**, **8c**, and **8d**, the competitive binding curves also did not reach the lower FA plateau, but these FA values were correctly determined by nonlinear regression.

### SPR Analysis of Binding Kinetics

The SPR analyses were carried out on a Biacore T200 (GE Healthcare, Chicago, IL, USA) at 25 °C using CM5 sensor chips (Cytiva) and HBS-P+ as running buffer (Cytiva) at a flow of 30 μL/min unless otherwise specified and a data collection rate of 10 Hz. All flow cells (FC) were normalized, and functionalization was performed using an amine coupling Kit (Cytiva). The amine coupling comprises the surface activation by EDC/NHS, the coupling procedure on the active flow cell (FCactive) using a solution of human nectin-4 (R&D Systems, Catalog number 2659-N4) in 10 mM acetate buffer (pH 4.5) for 700 s at a flow of 5 μL/min), which was not performed for the reference flow cell (FCreference), and the blocking of the surface using 1 M ethanolamine (pH 8.5). By that, 183 RU (FC­(reference)) and 2372 RU (FC­(active)) were immobilized on the sensor surface. Binding analyses were performed by a single-cycle kinetic on both FC. Two start-up cycles were performed at the beginning of each experimental set, comprising the injection of bicyclic peptide (50 nM) and analysis on both FC, followed by regeneration. Binding analysis for the bicyclic peptides was performed by consecutive injection of five increasing concentrations (120 s contact time for each concentration), followed by 600 s dissociation time and analysis on both FC. The regeneration was performed by sequential injection of 10 mM NaOAc (pH 5.5) for 60 s, washing the needle using HBS-P+, injection of 10 mM NaOAc (pH 5.5) for 60 s, 600 s waiting, four injections of HBS-P+ (60 s), and a final 600 s waiting for stabilization. Blank runs using only buffer (HBS-P+) instead of the sample were carried out to obtain double-referenced chromatograms. Binding analyses were performed unless otherwise stated in a concentration range between 0.16–100 nM and 1.6–1000 nM (5-fold dilution series) and analyzed in triplicate. The data were analyzed using the Biacore Evaluation software 3.2.1. Each sensorgram was reference subtracted (FC­(active)-FC­(reference)) and blank corrected. Data were fitted to a 1:1 binding model.

### Radiolabeling and Radiopharmacological In Vitro Characterization of DOTA/NODAGA-Bearing Peptides

#### Radiolabeling

[^64^Cu]­CuCl_2_ was produced at the Helmholtz-Zentrum Dresden-Rossendorf on the 30 MeV TR-Flex-cyclotron (Advanced Cyclotron Systems Inc., ACSI, Canada) by ^64^Ni­(p,n)^64^Cu nuclear reaction as reported previously.^50,64^ Peptides (1 μL of 2 mM DMSO stock, 2 nmol) were labeled with [^64^Cu]­CuCl_2_ (50 MBq, 49 μL) in ammonium acetate buffer (pH 5.6, 25 min, 60 °C). Accordingly, the apparent molar activity (molar activities calculated based on the applied peptide amount, no separation of nonlabeled peptide was conducted after radiolabeling) was 25 GBq/μmol. The radioligand stock solutions (0.2 M NH_4_OAc, 1 MBq/μL, 40 μM) were diluted with PBS (10 mM, pH 7.4) or 0.154 M NaCl for further experiments. For ^64^Cu-labeling of **4** at a high apparent molar activity of 200 MBq/nmol, peptide **4** (0.5 μL of 2 mM DMSO stock, 1 nmol) was labeled with [^64^Cu]­CuCl_2_ (200 MBq, 199.5 μL) in ammonium acetate buffer (pH 5.6, 25 min, 60 °C).

[^68^Ga]­GaCl_3_ was eluted with 0.1 M HCl from a ^68^Ge/^68^Ga generator (Eckert & Ziegler). Peptides (2 μL of 2 mM DMSO stock, 4 nmol) were labeled with [^68^Ga]­GaCl_3_ (75 MBq, 250 μL) in sodium acetate buffer (pH 4.5, 10 min, 90 °C). Apparent molar activities of between 10 and 22 GBq/μmol were achieved. Preparation of radioligand stock solutions was performed as described above.

#### *n*-Octanol/PBS Distribution Coefficient (log*D*_7.4_ Value)

The determination of log*D*_7.4_ was performed in triplicate. A sample of the ^64^Cu/^68^Ga-labeled compound containing ≈1 MBq in a volume of 1 μL was added to a 1.5 mL Eppendorf tube containing 400 μL of PBS (pH 7.4) and 400 μL of *n*-Octanol (the phases were presaturated with each other). The tube was vortexed vigorously for 1 min and then centrifuged at 16,100*g* for 4 min to separate the phases. For sampling from the aqueous phase, the pipette tip was discharged while passing through the octanol layer. Then, the sample was taken within the aqueous phase. Any residual liquid was carefully removed by moving the tip along the inner wall of an Eppendorf tube. The radioactivity in a defined volume of each layer was measured (ISOMED 2100). The distribution coefficient was expressed as the logarithm of the ratio of counts per minute (cpm) measured in the n-Octanol phase to the cpm measured in the PBS phase.

#### Oxidation Stability

The stability toward oxidizing conditions was assessed with H_2_O_2_. For this purpose, the radioligands (2 nmol, 48 μL of labeling mixture) were treated with H_2_O_2_ (2 μL of 0.03%, final concentration of 0.0012%) at ambient temperature. At distinct time points (3, 5, and 7 h), an aliquot of 2 μL was withdrawn and diluted with 48 μL of a mixture named “Supersol,” which consists of 20% ethanol, 0.5% Triton X-100, 5 mM EDTA, 0.5 mM *o*-phenanthroline, and 0.1% saponin. “Supersol” was used for dilution due to the better solubilization of hydrophilic compounds as compared to CH_3_CN/water mixtures or CH_3_CN alone. The resulting solutions were analyzed by radio-HPLC (same system as that used for analyzing the ^64^Cu-labelings).

#### Plasma Stability

Human plasma was collected as previously described.^57^ For assessing the stability in human plasma, 10 μL (10 MBq) of the labeling solution of **[** ^**64**^ **Cu]­Cu-1e**, **[** ^**64**^ **Cu]­Cu-3a**, or **[** ^**64**^ **Cu]­Cu-4** were added to 90 μL of human plasma, and the mixture was incubated at 37 °C for up to 24 h. At distinct time points (1, 2, 4, and 24 h), an aliquot of 20 μL was withdrawn and diluted with 60 μL of a mixture named “Supersol” (composition as described above). This was followed by centrifugation at 16,100*g* for 2 min. The supernatant was analyzed by radio-HPLC (the same system as used for analyzing the ^64^Cu-labelings).

#### Analysis of Urothelial Carcinoma Cell Models Toward the Presence of Nectin-4

Two urothelial carcinoma (UC) cell lines, HT-1376 and 5637, were analyzed with regard to their nectin-4 content for direct quantification and IHC staining with Western Blot, immunofluorescent staining, flow cytometry, and ELISA. For all methods, cell lysates were prepared from both cell lines. To this end, a confluent cell layer of the respective cells was washed three times with cold PBS+, followed by the addition of RIPA lysis buffer and incubation for 10 min on ice. The flask was then scraped with a cell scraper, and the lysate was transferred to a tube, followed by centrifugation at 14,000×*g* for 15 min at 4 °C before the supernatant was transferred to a fresh tube, and the protein concentration of the lysate was determined by a Detergent Compatible (DC) Protein Assay (Bio-Rad # 5000112).

For Western Blot, an SDS-PAGE was prepared from the 5637 and HT-1376 cell lysates (25 μg per lane), which was subsequently blotted onto a membrane (Cytiva # RPN3032D). The membrane was then probed with an antinectin-4 antibody (ThermoFisher #PA5-47365), which was diluted 1:200 in TBS-T + 3% BSA and incubated overnight at 4 °C. After three washes with TBS-T, the membrane was finally incubated with an antigoat IgG HRP conjugate (Sigma #A5420) diluted 1:20,000 in TBS-T + 3% BSA for 2 h at ambient temperature. After three washing steps with TBS-T, the bands were visualized using the SuperSignal West Femto Maximum Sensitivity Substrate (ThermoFisher no. 34095) and the CelvinS Chemiluminescence Imager (Biostep).

For immunofluorescent staining, the cells were seeded in a chamber slide and cultured for 3 days before staining. The cells were quickly washed with media and then incubated with 30 nM Enfortumab (MCE #HY-P99016) in fresh media at 4 °C on ice for 1.5 h. The cells were subsequently washed three times with PBS and fixed in 4% PFA + 2.5% sucrose for 20 min, followed by staining with anti-human IgG AlexaFluor-488 antibody (ThermoFisher #A11013) according to manufacturer instructions. The cell membrane was stained with a WGA-CF633 conjugate (Biotinum #29024_1), which was diluted 1:200 in PBS and incubated on the cells for 8 min. Finally, the cell nucleus was stained with Hoechst33258 (Sigma #B1155) diluted 1:20 in PBS. After 15 min of incubation at ambient temperature, the staining solution was discarded, and the cells were washed three times with PBS. For fluorescent staining with probe **1a**, cells were seeded in a chamber slide and cultured for 3 days. Subsequently, the media was discarded, cells were rinsed with fresh media, and **1a** was diluted in media was added to the cells (final concentration of 1 μM). After 1.5 h of incubation on ice, the cells were washed three times with cold PBS+ and fixed in 4% PFA + 2.5% sucrose for 20 min. WGA-CF633 and Hoechst33342 staining were performed as described above. Fluorescent staining was visualized with the Evident Olympus Fluoview FV 4000.

For flow cytometry analysis, HT-1376 and 5637 cells were rinsed with PBS three times and dissociated from a flask by using 50 mM EDTA and a cell scraper. Aliquots of the cell suspension containing 1 × 10^6^ cells were pelleted at 300×*g* for 7 min, and one pellet of each cell line was dissolved in fresh PBS + 3% BSA containing 1 μM of probe **1b**. As a negative control, one HT-1376 pellet was dissolved only in PBS + 3% BSA. The cells were incubated on ice for 1 h before they were pelleted again. The staining solution was discarded, and the pellet was washed in 1 mL of PBS + 0.5% BSA + 2 mM EDTA (FACS wash buffer) and centrifuged again. The washing step was repeated a total of three times. After the last wash, the pellets were dissolved in 500 μL of FACS wash buffer and analyzed with an Attune NxT Flow Cytometer (Invitrogen). For the detection, the BL1 laser with an excitation of 488 nm and an emission filter of 530/30 nm set to a Voltage of 270 was used. The forward scatter was set to a voltage of 80 or 120, and the sideward scatter was set to 320 or 370 for HT-1376 or 5637 cells, respectively. A sample of 50 μL was analyzed at a speed of 100 μL/min, leading to a total of 35410 and 34992 analyzed events (singlets) for HT-1376 and 5637 cells, respectively.

To quantify the amount of nectin-4 in cell lysates as well as tumor lysates, the human nectin-4 ELISA Kit – Quantikine (#DNEC40) from R&D Systems was used according to manufacturer instructions. Cell lysates were prepared as described for Western Blot analysis above using the kit-compatible lysis buffer 2 (R&D # 895347). Tumor lysates were prepared in the same buffer using the gentleMACS dissociator (Miltenyi Biotec).

IHC was performed for tumor sections of 5637- and HT-1376 tumors. In preparation for the IHC, tumors were excised from the animals and fixed in 4% PFA + 2.5% sucrose over a period of 2 days. Subsequently, the samples were embedded in paraffin and finally sectioned with a Mikrotom HM 340E (Thermo Scientific) to produce 5 μm sections on glass slides. These sections were prepared for IHC as follows: First, they were stripped of the paraffin by two incubations in fresh Roti-Clear for 15 min, followed by 5 min incubation in 100% ethanol, 96% ethanol, 85% ethanol, 70% ethanol, 50% ethanol, and distilled water. The slides were then transferred into a 0.5 mM TRIS/1 mM EDTA buffer (pH 9) and steamed at 95 °C for 20 min. After cooling, they were washed with TBS. Afterward, the sections were blocked by incubation with 3% H_2_O_2_ in TBS-T, followed by treatment with Avidin-Solution (Vector Laboratories #SP-2001) and treatment with Biotin Solution (Vector Laboratories #SP-2001) for 10 min each. After each blocking step, the slides were washed with TBS and finally incubated in 10% FCS in TBS-T for 1.5 h. The primary antinectin-4 antibody (ThermoFisher #PA5-4765) was applied at a dilution of 1:20 in 10% FCS in TBS-T overnight. Normal IgG goat (Santa Cruz #sc-2028) was used as an antibody isotype control in the same concentration. The next day, the slides were washed twice with TBS-T and once with TBS, and the secondary antibody, biotinylated antigoat IgG (Dianova #705-065-003), was applied at a dilution of 1:200 in 10% FCS in TBS-T. After 1 h of incubation, the washing steps were repeated. The staining of the sections was achieved by 30 min of incubation with ExtrAvidin-Peroxidase (Sigma #E2886) 1:50 in TBS-T, followed again by washing twice with TBS-T and once with TBT and detection with the AEC Substrate (BD-Pharmingen #551015) for approximately 5 min. For counterstaining, hematoxylin staining of the cell nuclei was performed. The slides were mounted, and images were taken with the AxioVison Zeiss.

#### Cell Binding and Internalization at 10 nM of Radioligand

HT-1376 cells were cultured in DMEM and 5637 cells in RPMI media supplemented with 4.5 g/L d-glucose (GlutaMAX), 10% FBS, and 1% Penicillin/Streptomycin at 5% CO_2_ at 37 °C. Cells were seeded in 48-well plates 4 days prior to the experiment (HT-1376:50,000 cells/well, 5637:30,000 cells/well). For the binding assay, DMEM media was used for both cell types. Prior to the experiment, all cells were washed once with fresh media. For determining the total binding, the radioligand was diluted to 10 nM in DMEM media and added to the cells. Nonspecific binding of the radioligand was determined in the presence of compound **1d** (1 μM in well) or **3a** (for **[** ^**68**^ **Ga]­Ga-8d**, 1 μM in well). The plates were incubated at 37 °C for 1 h (shaken at 300 rpm). After incubation, cells were washed twice with cold PBS+ for approximately 5 min. For determining internalization, one wash step with cold PBS+ was replaced by treatment with cold glycine buffer (50 mM, pH 2.8) for 5 min. Subsequently, the cells were lysed with 0.1% SDS in 0.1 M NaOH. The lysates were measured in a γ-counter (PerkinElmer Wizard 3”). The results were normalized to the protein content (measured at *A*_280nm_ with setting 1 Abs = 1 mg/mL) of the lysates determined with a NanoDrop spectrophotometer (Thermo-Fisher Scientific). Specific binding was calculated by subtraction of total and nonspecific binding data.

To measure the time-dependent binding of **[** ^**64**^ **Cu]­Cu-4** to HT-1376 cells, the cells were prepared as described above. The incubations with 10 nM radioligand in DMEM (total binding) or 10 nM radioligand mixed with 1 μM of compound **1d** (nonspecific binding) were started at the indicated temperature. The incubations were stopped at the time points of interest (5, 15, 30, 1, 2, and 4 h) by washing with cold PBS+ twice (specific total binding) or washing once with cold PBS+ followed by washing with cold glycine buffer (50 mM, pH 2.8) for 5 min (specific internalized fraction of radioligand). The cells were lysed, and the lysate was measured as described above. The difference between the corresponding values for specific total binding and the specific internalized fraction was calculated and equals the surface-bound fraction of the radioligand.

#### Saturation Binding Assay

For the saturation binding assay, total and nonspecific binding were determined as described above using 10 different radioligand concentrations (0.156–80 or 0.321–160 nM) prepared by serial 1:1 dilution. Plots of “total binding” = f­(radioligand) were analyzed by nonlinear regressions using the model of “one site-total, accounting for ligand depletion” as implemented in GraphPad Prism, and Plots of “nonspecific binding” = f­(radioligand) were analyzed by linear regressions.^68^

#### Cellular Efflux Assay

Initially, cell binding at 10 nM radioligand was determined in the presence and absence of 1 μM **1d** as described above. After the 1 h incubation period, the radioligand was discarded, and the cells were very briefly washed with PBS+. Quickly afterward, cells in wells that served as *t*_0_ control (*t* = 0 min) were lysed. New media was added to all other wells, and incubation at 37 °C and 300 rpm was continued. At the chosen time points (5, 15, 30 min, 1, 2, and 4 h), the media from all wells were transferred to separate tubes, and the cells in the wells of the respective time point were lysed. All other wells were refilled with new media and further incubated. The media samples as well as the cell lysates were measured in the γ-counter (PerkinElmer Wizard 3”) afterward. The counts of the respective wells of nonspecific binding were subtracted from the counts measured from total binding to determine the portion of specific counts. Specific counts of the media samples from the same wells were added up to account for the total dissociated ligand. The specific counts measured in the cell lysate represent the residual bound ligand.

### Experimental Animals

All animal experiments were performed following the protocols evaluated and approved by the Landesdirektion Sachsen, Referat 25 – Veterinärwesen, Lebensmittelüberwachung and Pharmazie (09105 Chemnitz, Germany, ethics approval number: 25-5131/562/52). HT-1376- and 5637-derived tumor xenograft models were established in NMRI nude mice (Rj: NMRI-Foxn1nu/nu, Charles River Laboratories, Sulzfeld, Germany) by subcutaneous injection of 5 × 10^6^ cells in 100 μL of PBS containing 50% (v/v) Matrigel (Corning Life Sciences, Amsterdam, The Netherlands).

### Small Animal PET/CT Imaging and Reconstruction

When the tumor volume reached 200 mm^3^, PET experiments with radiotracers were performed. Radiolabeling of the ligands was performed as described previously with an apparent molar activity of 25 MBq/nmol. The radioligand stock solution (pH 6.5 – 7.5) was diluted in 0.9% NaCl, and 200 μL containing ∼9 MBq (≈ 0.46 nmol) was injected per mouse intravenously into a lateral tail vein. For blocking of target-specific radioligand binding, 75 nmol of blocking substance (**1d**) was administered simultaneously with the radioligand. Coincidences were recorded continuously in a 2 h PET-scan starting with the injection. Additionally, a CT scan was performed for anatomical referencing and attenuation correction. PET/CT imaging, reconstruction, and tissue delineation were carried out as described previously.^59^

### First-in-Human Application

First-in-human application and data analysis was performed after informed consent as part of an individual diagnostic concept, which was recommended by the interdisciplinary tumor conference of the Comprehensive Cancer Center.

**[** ^**68**^ **Ga]­Ga-NECT-224** was synthesized in an automated ML EAZY synthesis module employing the C0-GA-PEP cassettes with Sep-Pack Light Accell Plus CM cartridges for postpurification.^83^ The molar activity was determined to be 32 MBq/nmol at a radiochemical yield of 87.4% and radiochemical purity of greater than 98%. All quality control tests were performed as a prerequisite for human use, including radiochemical purity, chemical purity, endotoxin, and sterility testing.

The PET/CT Scan was performed 41 minutes after the injection of 149 MBq of **[** ^**68**^ **Ga]­Ga-NECT-224**, according to previous experiences with **[^68^Ga]­Ga-N188**,^84^ on a Siemens Biograph Vision 600 instrument (Siemens Healthineers, Knoxville, TN, USA). The emission PET scan was obtained using continuous bed motion with a speed of 1.4 mm/s from just above the skull base to mid-thigh in caudo-cranial direction. Images were reconstructed using the TrueX algorithm with 4 iterations, 5 subsets, a time-of-flight (TOF) application, and a 5 mm Gaussian filter. A low-dose CT was acquired (X-ray tube current of 10 mAs, tube voltage of 120 kV, spiral pitch factor 3.0 mm slice thickness), which was used for scatter correction of the subsequent PET scan. This was supplemented with a diagnostic CT after the PET scan, with a 70 s delay after injection of 60 mL Accupaque 350. The MR examination was performed consecutively on a 3T Magnetom Lumina scanner (Siemens Healthineers, Erlangen, Germany) using a dedicated head and neck coil. After administration of 12 mL Gadovist (gadobutrol), the following sequences were acquired: T1-weighted SPACE with fat suppression in transverse (T1 SPACE FS), shown in Figure 1, and coronal planes; T2-weighted turbo spin echo (T2 TSE) in the transverse plane; diffusion-weighted imaging (DWI); and apparent diffusion coefficient (ADC) maps.

## Supplementary Material





## Notes

Time-dependent FA values for binding of probes **1a** and **1b** to nectin-4; Competitive binding curves of nonfluorescent peptides and enfortumab using probe **1a**; Exemplary SPR sensorgrams; Test for validity of a two-state reaction for **8c** by SPR; ECD and UV spectra of **1d** and **1e**; Temperature-dependent TOCSY correlation signals reveal distinct hydrogen bond environments; Observed ^1^H,^15^N-HSQC correlations at 25 °C; Side-by-side comparison of analytical radio-HPLC chromatograms after radiosynthesis of **[** ^**64**^ **Cu]­Cu-1e** (bearing Met^4^) and **[** ^**64**^ **Cu]­Cu-7** (bearing Cys­(et)^4^); Exemplary analytical radio-HPLC chromatograms of the radiolabeled peptides upon incubation in diluted H_2_O_2_ solution; Exemplary radio-HPLC chromatograms of **[** ^**64**^ **Cu]­Cu-1e**, **[** ^**64**^ **Cu]­Cu-3a**, and **[** ^**64**^ **Cu]­Cu-4** upon incubation in vitro in PBS and human plasma; Assessment of plasma protein and HSA binding for **[** ^**64**^ **Cu]­Cu-1e** and **[** ^**64**^ **Cu]­Cu-4** by ultrafiltration; Isotype control for immunohistochemical staining of HT-1376 tumor slices; Time-dependent binding of **[** ^**64**^ **Cu]­Cu-4** (100 nM) at 37 and 4 °C to 5637 cells; Saturation binding of **[** ^**68**^ **Ga]­Ga-8d** (**[** ^**68**^ **Ga]­Ga–N188**) with data for total, nonspecific and calculated specific binding; Time-activity curves of the ^64^Cu- and ^68^Ga-labeled ligands for muscle, kidney, liver, and urinary bladder uptake; Tumor uptake of **[** ^**64**^ **Cu]­Cu-1e** and **[** ^**64**^ **Cu]­Cu-3a** under blocking conditions; PET images and derived data for **[** ^**64**^ **Cu]­Cu-1e** at 24 h *p.i.;* PET images and derived data for **[** ^**64**^ **Cu]­Cu-4** and **[** ^**64**^ **Cu]­Cu-8b** at low molar amounts of total radioligand; Side-by-side comparison of time-activity curves of **[** ^**64**^ **Cu]­Cu-1e** (bearing Met^4^) and **[** ^**64**^ **Cu]­Cu-2** (bearing Met­(O)^4^); General synthesis of the bicyclic peptides; IC_50_ values and Hill coefficients (n) determined with the FA based competitive binding assay; Preliminary assignments of ^1^H NMR chemical shifts; NMR spectra of **1d**, CHI IAM_7.4_ values for selected peptides; Blood kinetic parameters for the ^64^Cu-labeled ligands; Blood kinetic parameters for the ^68^Ga-labeled ligands; Compound characterization data (PDF)
Molecular formular strings (CSV)

## Abbreviations

FA: fluorescence anisotropy
Ig: immunoglobulin
MMAE: monomethyl auristatin E
PET: positron emission tomography
RIPA: radioimmunoprecipitation assay buffer
SUV: standardized uptake value
TAC: time-activity curve
TATA: 1,3,5-triacryloyl-1,3,5-triazinane
TCEP: tris­(2-carboxyethyl)­phosphine
TMBM: 1,3,5-tris­(bromomethyl)­benzene
