# Supplementary material for: “Precision on Two Wheels”—Structural Refinement of 64Cu- and 68Ga-Labeled Bicyclic Peptides Targeting Nectin‑4 for Improved Tumor Imaging: From Preclinical Development to First-in-Human Application
Source: J Med Chem. 2025 Oct 13;68(20):21962–87. doi: 10.1021/acs.jmedchem.5c02371 (PMC12557367; doi:10.1021/acs.jmedchem.5c02371)
Supplement: Supplementary file 1 [file jm5c02371_si_001.pdf]

Supporting Information

"Precision on two wheels" – Structural refinement of ^{64}Cu- and ^{68}Ga-labeled bicyclic peptides targeting nectin-4 for improved tumor imaging: from preclinical development to first-in-human application

Tobias Krönke,[a,#] Johanna Trommer,[a,#] Martin Ullrich,[a] Markus Laube,[a] Reik Löser,[a,f], Jérôme Kretzschmar,[b] Marie Urbanova,[c] Sven Stadlbauer,[a,f] Florian Brandt,[d] Ivan Platzek[d] Sebastian Hoberück,[d] Jörg Kotzerke,[d] Christian Thomas,[e] Matthias Miederer,[a,g,i] Ralph A. Bundschuh,[a,d,h] Klaus Kopka,[a,g,h] Jens Pietzsch,[a,f] Robert Wodtke[a,*]

[a] Helmholtz-Zentrum Dresden-Rossendorf, Institute of Radiopharmaceutical Cancer Research, Bautzner Landstraße 400, 01328 Dresden, Germany

[b] Helmholtz-Zentrum Dresden-Rossendorf, Institute of Resource Ecology, Bautzner Landstraße 400, 01328 Dresden, Germany

[c] University of Chemistry and Technology, Department of Physics and Measurements, Technická 5, Prague 16628, Czech Republic

[d] University Hospital Carl Gustav Carus at the Technische Universität Dresden, Klinik und Poliklinik für Nuklearmedizin, Fetscherstraße 74, 01307 Dresden, Germany

[e] University Hospital Carl Gustav Carus at the Technische Universität Dresden, Klinik und Poliklinik für Urologie, Fetscherstraße 74, 01307 Dresden, Germany

[f] Technische Universität Dresden, School of Science, Faculty of Chemistry and Food Chemistry, Mommsenstraße 4, 01069 Dresden, Germany

[g] National Center for Tumor Diseases (NCT), NCT/UCC Dresden, a partnership between DKFZ, Faculty of Medicine and University Hospital Carl Gustav Carus, TUD Dresden University of Technology & Helmholtz-Zentrum Dresden-Rossendorf (HZDR), 01307 Dresden, Germany.

[h] German Cancer Consortium (DKTK), Partner Site Dresden, 01307 Dresden, Germany

[i] German Cancer Research Center (DKFZ), 69120 Heidelberg, Germany

# These authors contributed equally to this work.

*E-mail: r.wodtke@hzdr.de

Table of Contents

Figure S1: **Time-dependent FA values for binding of probes 1a and 1b to nectin-4 .... 4**

Figure S2: **Competitive binding curves of non-fluorescent peptides and enfortumab using probe 1a 5**

Figure S3: **Exemplary SPR sensorgrams 8**

Figure S4: **Test for validity of a two-state reaction for 8c by SPR 12**

Figure S5: **ECD (A) and UV (B) spectra of 1d and 1e 14**

Figure S6: **Temperature-dependent TOCSY correlation signals reveal distinct hydrogen bond environments 15**

Figure S7: **Observed ^{1}H,^{15}N-HSQC correlations at 25 °C 16**

Figure S8: **Side-by-side comparison of analytical radio-HPLC chromatograms after radiosynthesis of [^{64}Cu]Cu-1e (bearing Met^{4}) and [^{64}Cu]Cu-7 (bearing $Cys(et)^{4}$) 17**

Figure S9: **Exemplary analytical radio-HPLC chromatograms of the radiolabeled peptides upon incubation in diluted H_2O_2 solution 18**

Figure S10: **Exemplary radio-HPLC chromatograms of [^{64}Cu]Cu-1e, [^{64}Cu]Cu-3a, and [^{64}Cu]Cu-4 upon incubation *in vitro* in PBS and human plasma 19**

Figure S11: **Assessment of plasma protein and HSA binding for [^{64}Cu]Cu-1e and [^{64}Cu]Cu-4 by ultrafiltration 20**

Figure S12: **Isotype control for immunohistochemical staining of HT-1376 tumor slices 21**

Figure S13: **Time-dependent binding of [^{64}Cu]Cu-4 (100 nM) at 37°C (A) and 4°C (B) to 5637 cells 22**

Figure S14: **Saturation binding of [^{68}Ga]Ga-8d ([^{68}Ga]Ga-N188) 23**

Figure S15: **Time-activity curves of the ^{64}Cu- (top) and ^{68}Ga-labeled ligands (bottom) for muscle, kidney, liver, and urinary bladder uptake 24**

Figure S16: **Tumor uptake of [^{64}Cu]Cu-1e and [^{64}Cu]Cu-3a under blocking conditions ... 25**

Figure S17: **PET images and derived data for [^{64}Cu]Cu-1e at 24 h *p.i.* 27**

Figure S18: **PET images and derived data for [^{64}Cu]Cu-4 and [^{64}Cu]Cu-8b at low molar amounts of total radioligand 28**

Figure S19: **Side-by-side comparison of time-activity curves of [^{64}Cu]Cu-1e (bearing Met^{4}) and [^{64}Cu]Cu-2 (bearing $Met(O)^{4}$) 29**

Scheme S1: **General synthesis of the bicyclic peptides 30**

Table S1: **IC_{50} values and Hill coefficients (n) determined with the FA based competitive binding assay 31**

Table S2: **Preliminary assignments of ^{1}H NMR chemical shifts for 1d at 70°C 32**

NMR spectra of 1d 33

- ^{1}H-NMR spectra of 1d at various temperatures in DMSO-d_6 33
- COSY spectrum of 1d at 25 °C in DMSO-d_6 34
- TOCSY spectrum of 1d at 25 °C in DMSO-d_6 35
- ROESY spectrum of 1d at 25 °C in DMSO-d_6 36
- HSQC spectrum of 1d at 25 °C in DMSO-d_6 37
- HMBC spectrum of 1d at 25 °C in DMSO-d_6 38
- ^{1}H-NMR spectrum at 70°C in DMSO-d_6 39
- COSY spectrum of 1d at 70°C in DMSO-d_6 40
- TOCSY spectrum of 1d at 70°C in DMSO-d_6 41
- ROESY spectrum of 1d at 70°C in DMSO-d_6 42

HSQC spectrum of 1d at 70 °C in DMSO-d_6 43
HMBC spectrum of 1d at 70 °C in DMSO-d_6 44
Table S3: **CHI $IAM_{7.4}$ values for selected peptides 45**
Table S4: **Blood kinetic parameters for the ^{64}Cu-labeled ligands 46**
Table S5: **Blood kinetic parameters for the ^{68}Ga-labeled ligands 47**
Compound characterization data 48
Bicyclic peptides 48
Radiolabeled bicyclic peptides 70
References 77

Figure S1: Time-dependent FA values for binding of probes 1a and 1b to nectin-4

Plots of FA as a function of time after addition probe **1a** (left) or **1b** (right) to varying concentrations of nectin-4.

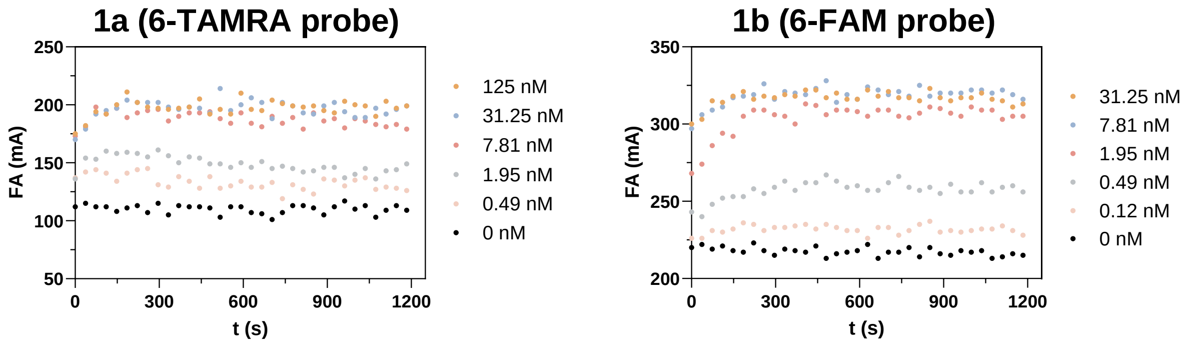

Figure S2: Competitive binding curves of non-fluorescent peptides and enfortumab using probe 1a

Data shown are mean values (±SD) of two experiments, each performed in duplicate. Conditions: 1 nM **1a**, 20 nM nectin-4, concentration range for non-fluorescent peptides covered at least three orders of magnitude, HEPES buffer (20 mM, pH, 7.4, 0.01% Tween20, 150 mM NaCl, 1% DMSO).

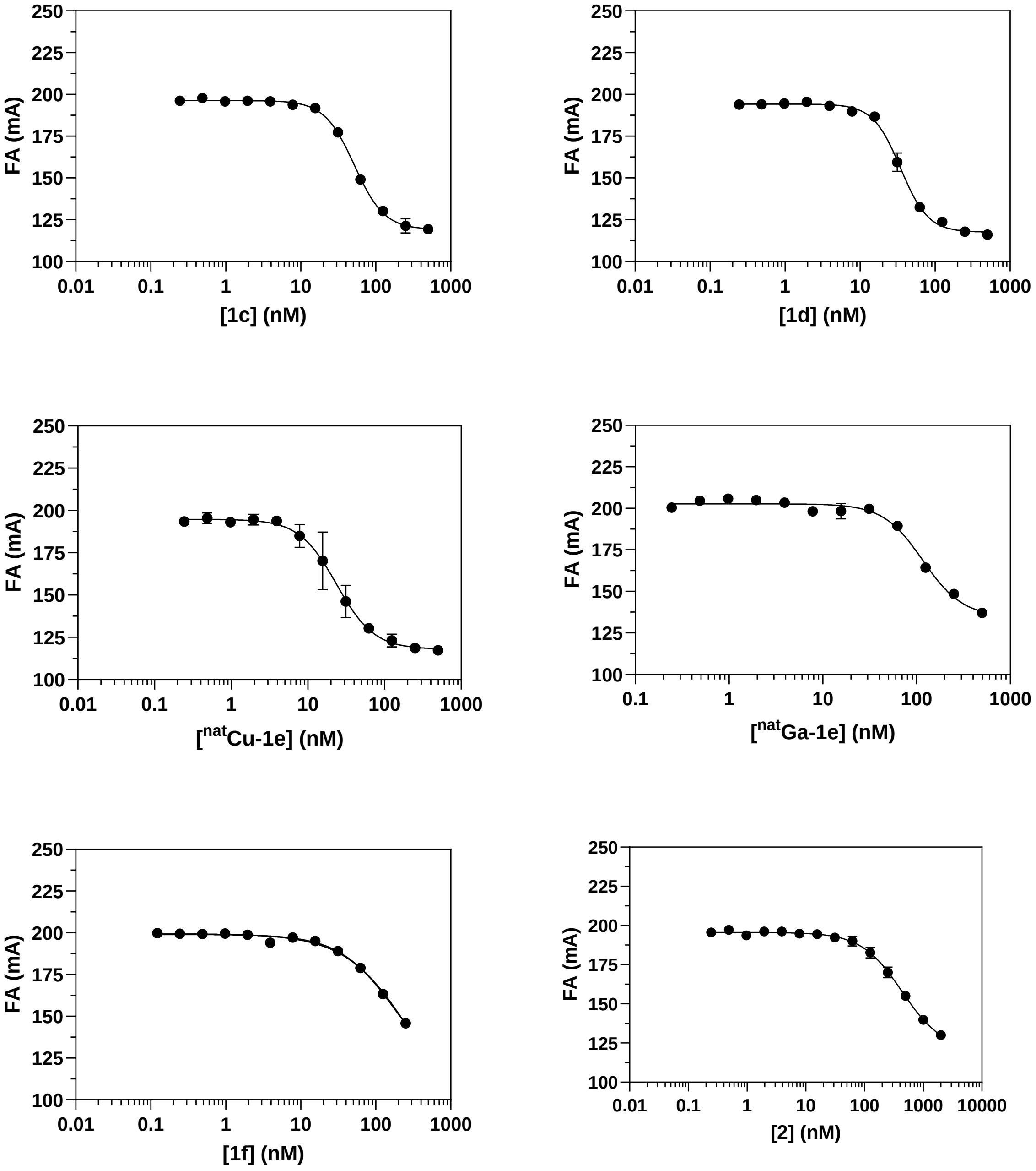

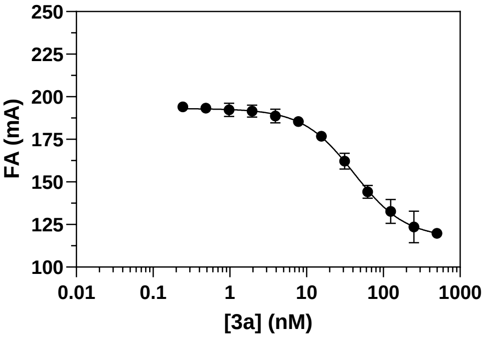
FA (mA)
250
225
200
175
150
125
100
0.01
0.1
1
10
100
1000
[3a] (nM)

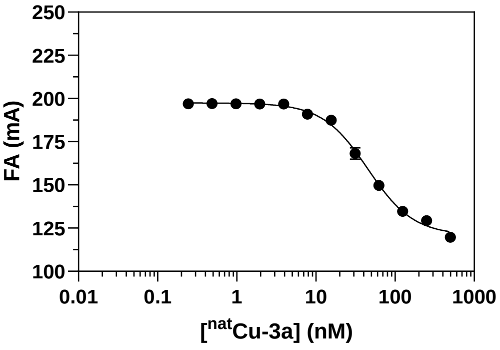
FA (mA)
250
225
200
175
150
125
100
0.01
0.1
1
10
100
1000
[natCu-3a] (nM)

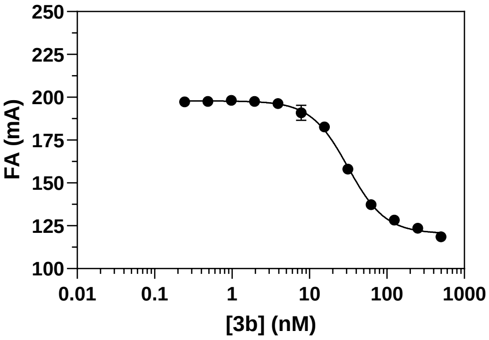
FA (mA)
250
225
200
175
150
125
100
0.01
0.1
1
10
100
1000
[3b] (nM)

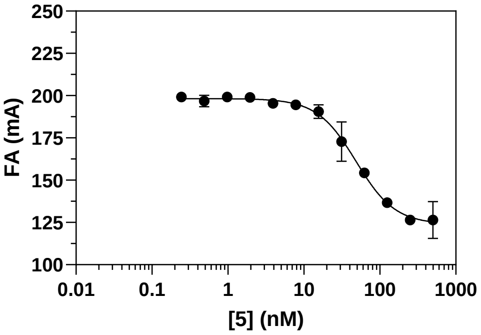
FA (mA)
250
225
200
175
150
125
100
0.01
0.1
1
10
100
1000
[5] (nM)

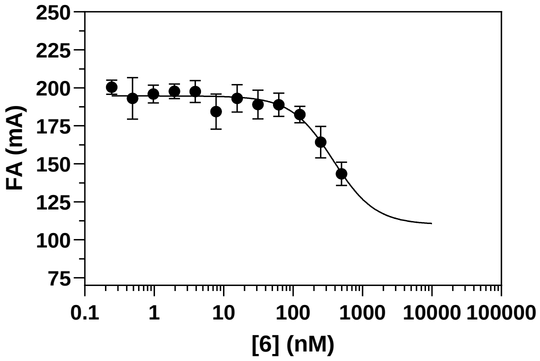
FA (mA)
250
225
200
175
150
125
100
75
0.1
1
10
100
1000
10000
100000
[6] (nM)

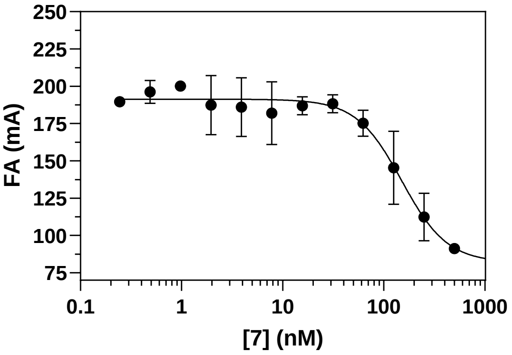
FA (mA)
250
225
200
175
150
125
100
75
0.1
1
10
100
1000
[7] (nM)

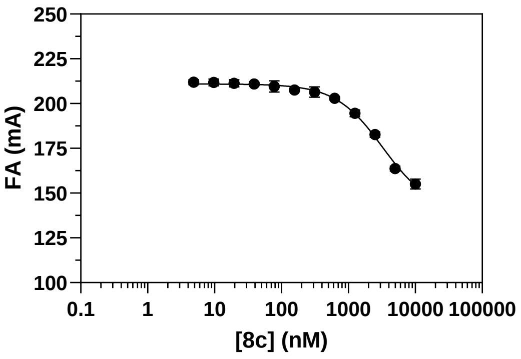
FA (mA)
250
225
200
175
150
125
100
0.1
1
10
100
1000
10000
100000
[8c] (nM)

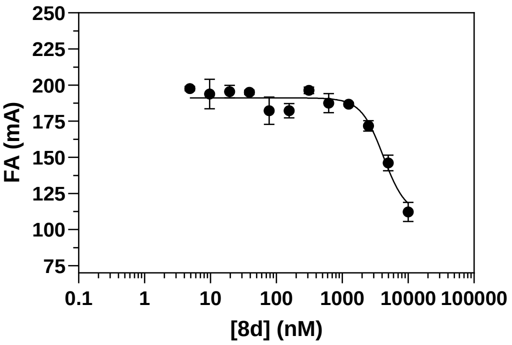
FA (mA)
250
225
200
175
150
125
100
75
0.1
1
10
100
1000
10000
100000
[8d] (nM)

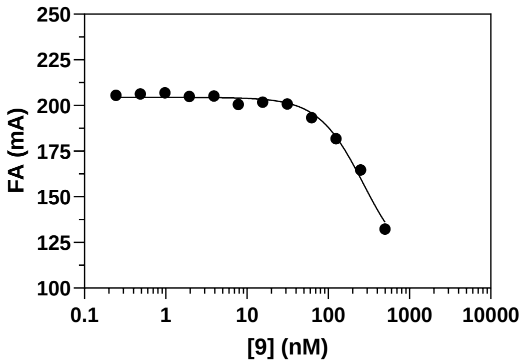
FA (mA)
[9] (nM)

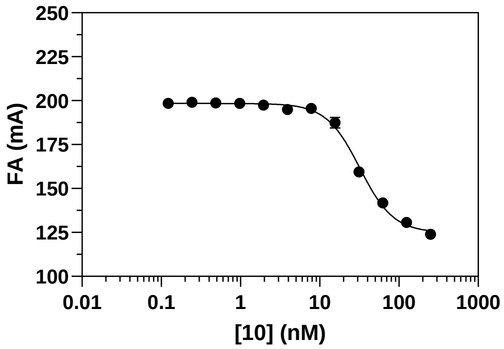
FA (mA)
[10] (nM)

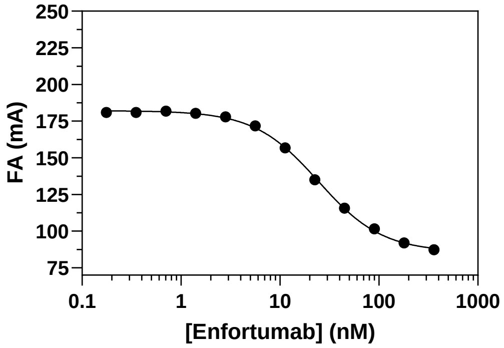
FA (mA)
[Enfortumab] (nM)

Figure S3: Exemplary SPR sensorgrams

Exemplary sensorgram for single-cycle-kinetic analysis of the bicyclic peptides (red) as analyte to immobilized human nectin-4 as well as the fit (black) as obtained according to a 1:1 binding model. Conditions: HBS-P+ buffer (pH 7.4).

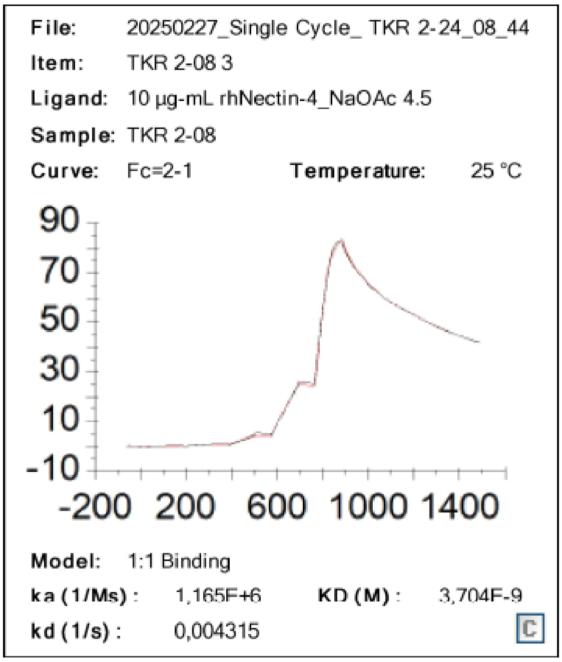

1e

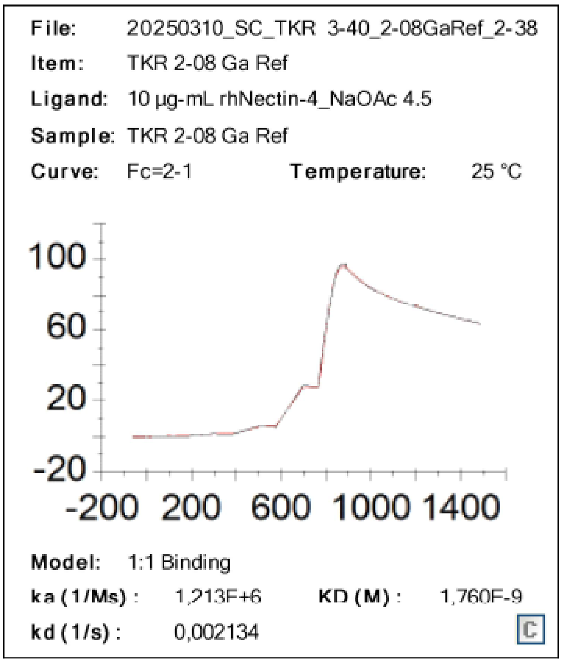

^{nat}Ga-1e

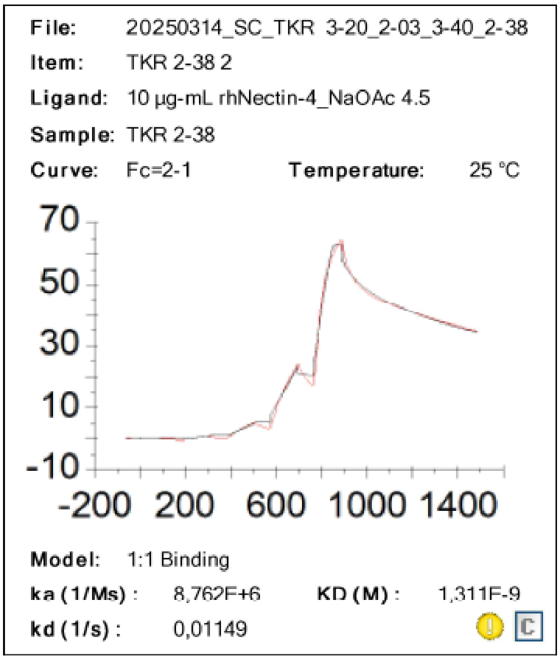
File: 20250314_SC_TKR 3-20_2-03_3-40_2-38
Item: TKR 2-38 2
Ligand: 10 µg-mL rhNectin-4_NaOAc 4.5
Sample: TKR 2-38
Curve: Fc=2-1
Temperature: 25 °C
70
50
30
10
-10
-200
200
600
1000
1400
Model: 1:1 Binding
ka (1/Ms) : 8,762E+6
KD (M) : 1,311E-9
kd (1/s) : 0,01149

1f

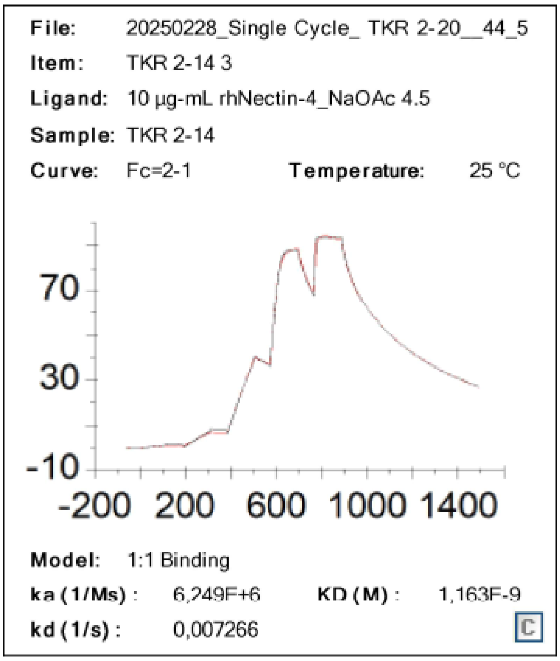
File: 20250228_Single Cycle_ TKR 2-20__44_5
Item: TKR 2-14 3
Ligand: 10 µg-mL rhNectin-4_NaOAc 4.5
Sample: TKR 2-14
Curve: Fc=2-1
Temperature: 25 °C
70
30
-10
-200
200
600
1000
1400
Model: 1:1 Binding
ka (1/Ms) : 6,249E+6
KD (M) : 1,163E-9
kd (1/s) : 0,007266

3a

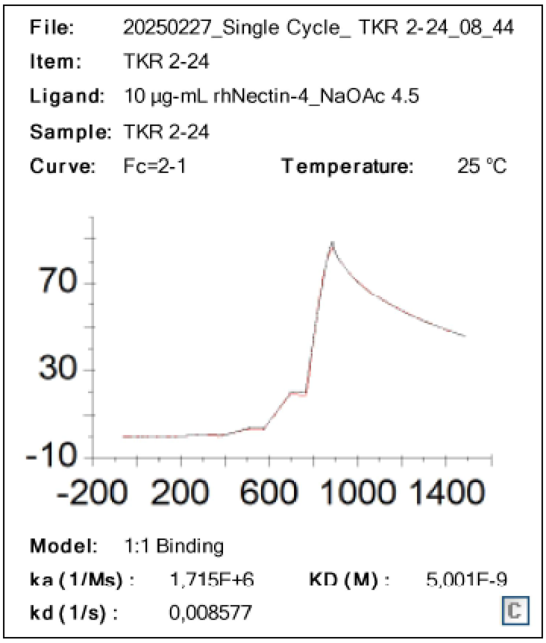
File: 20250227_Single Cycle_ TKR 2-24_08_44
Item: TKR 2-24
Ligand: 10 µg-mL rhNectin-4_NaOAc 4.5
Sample: TKR 2-24
Curve: Fc=2-1
Temperature: 25 °C
70
30
-10
-200
200
600
1000
1400
Model: 1:1 Binding
ka (1/Ms) : 1,715E+6
KD (M) : 5,001E-9
kd (1/s) : 0,008577

4

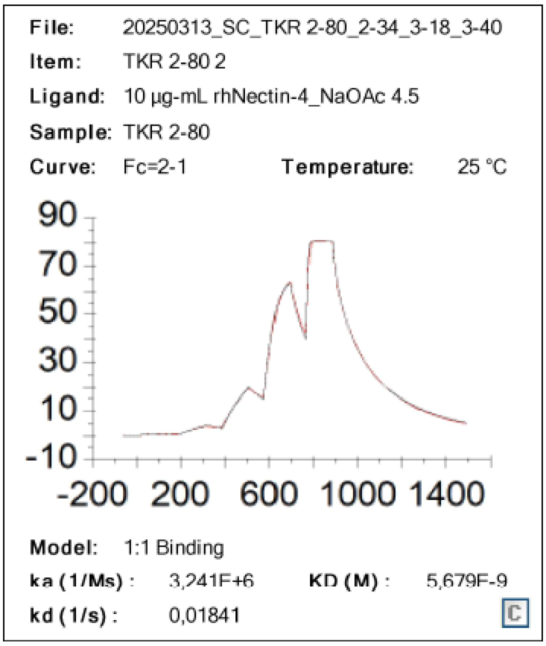
File: 20250313_SC_TKR 2-80_2-34_3-18_3-40
Item: TKR 2-80 2
Ligand: 10 µg-mL rhNectin-4_NaOAc 4.5
Sample: TKR 2-80
Curve: Fc=2-1
Temperature: 25 °C
90
70
50
30
10
-10
-200
200
600
1000
1400
Model: 1:1 Binding
ka (1/Ms) : 3,241E+6
KD (M) : 5,679E-9
kd (1/s) : 0,01841

6

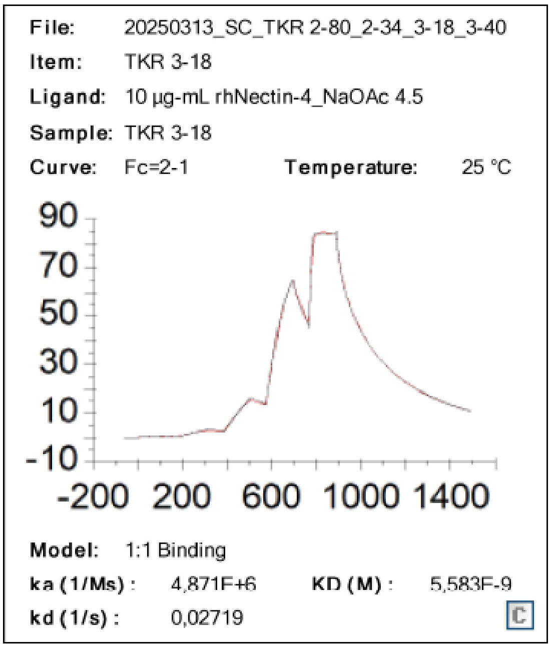

7

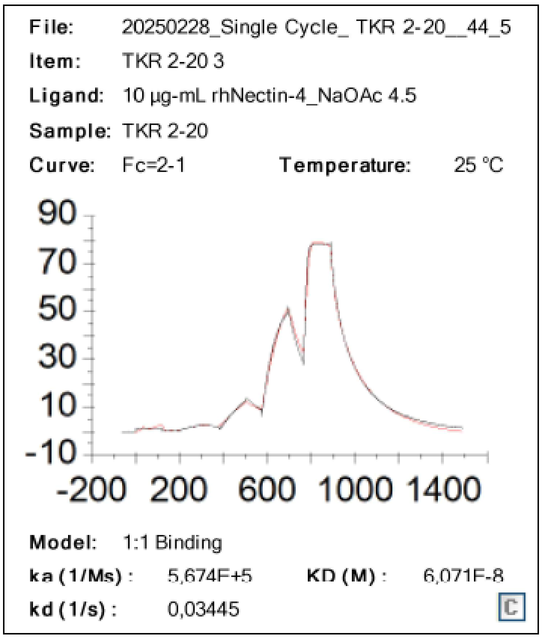

8a

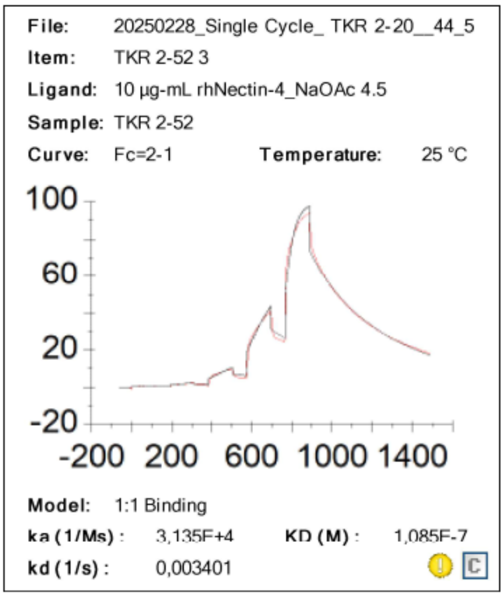

8c

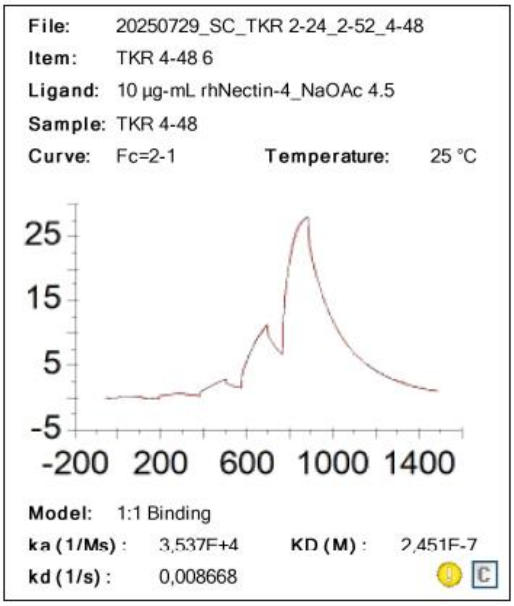

8d (N188)

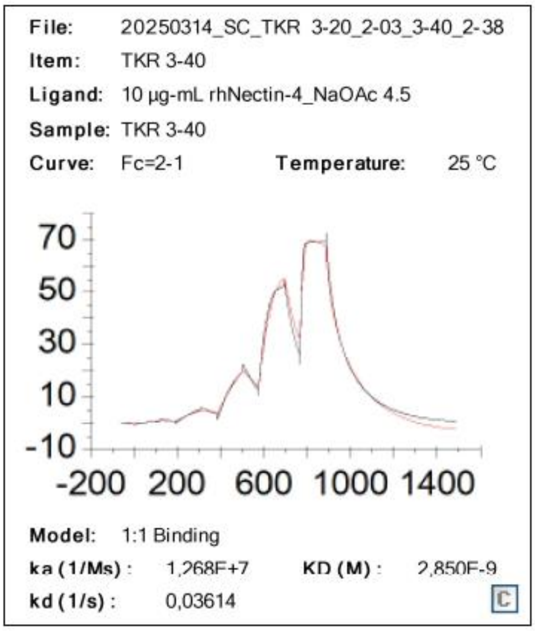
File: 20250314_SC_TKR 3-20_2-03_3-40_2-38
Item: TKR 3-40
Ligand: 10 µg-mL rhNectin-4_NaOAc 4.5
Sample: TKR 3-40
Curve: Fc=2-1
Temperature: 25 °C
70
50
30
10
-10
-200
200
600
1000
1400
Model: 1:1 Binding
ka (1/Ms) : 1,268E+7
KD (M) : 2,850E-9
kd (1/s) : 0,03614

9

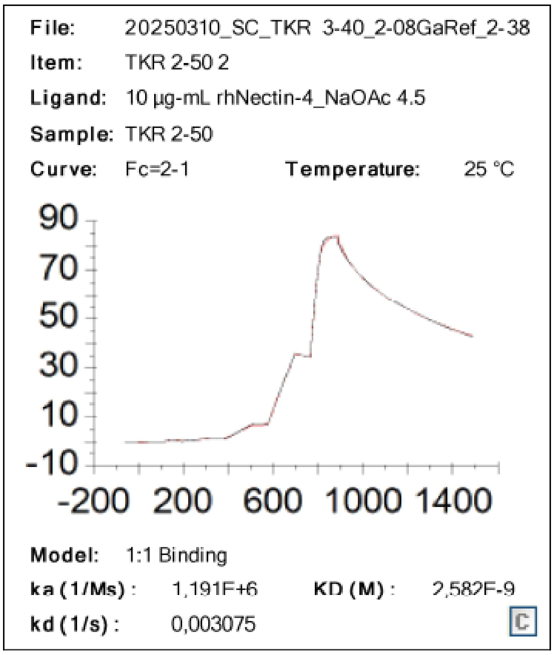
File: 20250310_SC_TKR 3-40_2-08GaRef_2-38
Item: TKR 2-50 2
Ligand: 10 µg-mL rhNectin-4_NaOAc 4.5
Sample: TKR 2-50
Curve: Fc=2-1
Temperature: 25 °C
90
70
50
30
10
-10
-200
200
600
1000
1400
Model: 1:1 Binding
ka (1/Ms) : 1,191E+6
KD (M) : 2,582E-9
kd (1/s) : 0,003075

10

Figure S4: Test for validity of a two-state reaction for 8c by SPR

It is worth noting that the SPR sensorgrams for compounds **1f** and **8c** (both bearing DOTA) showed a first rapid dissociation phase followed by second slower dissociation phase leading to deviations from ideal 1:1 binding. We initially interpreted this as a two-state reaction, which is characterized by 1:1 binding of analyte to the immobilized ligand followed by a conformational change that stabilizes the complex. To test for validity of a two-state reaction in this case, we analyzed **8c** as ligand of interest and **4** as reference ligand with normal 1:1 binding profile and injected the ligands at a concentration leading to immediate saturation (100 nM of **4** and 1000 nM of **8c**) using different association times (250 s, 500 s, 900 s) followed by dissociation for 600 s (Control Experiment – Linked Reaction). Regeneration was performed as described in the experimental section. In case of a two-state reaction, a slower dissociation is expected with prolonged association time. However, the results did not support a two-phase dissociation process, because dissociation was independent of association time (**A**, sensorgrams obtained after injection of **4** (red) and **8c** (green), adjusted for X=0 at binding and Y=0 at baseline (before injection start)). To more intuitively visualize this aspect, sensorgrams were adjusted to the binding response in **B** (sensorgrams obtained after injection of **8c**, adjusted for X=0 at binding, Y=0 at baseline (before injection start), Y=100 at binding (end of injection)) and **C** (sensorgrams obtained after injection of **4**, adjusted for X=0 at binding, Y=0 at baseline (before injection start), Y=100 at binding (end of injection)), showing that the dissociation rate was not changed with increased association time. We hence assume instead that both compounds **1f** and **8c** exhibit a weak nonspecific binding to the protein surface and/or sensor surface and analyzed the curves according to a 1:1 binding model as implemented in the Biacore Evaluation software 3.2.1.

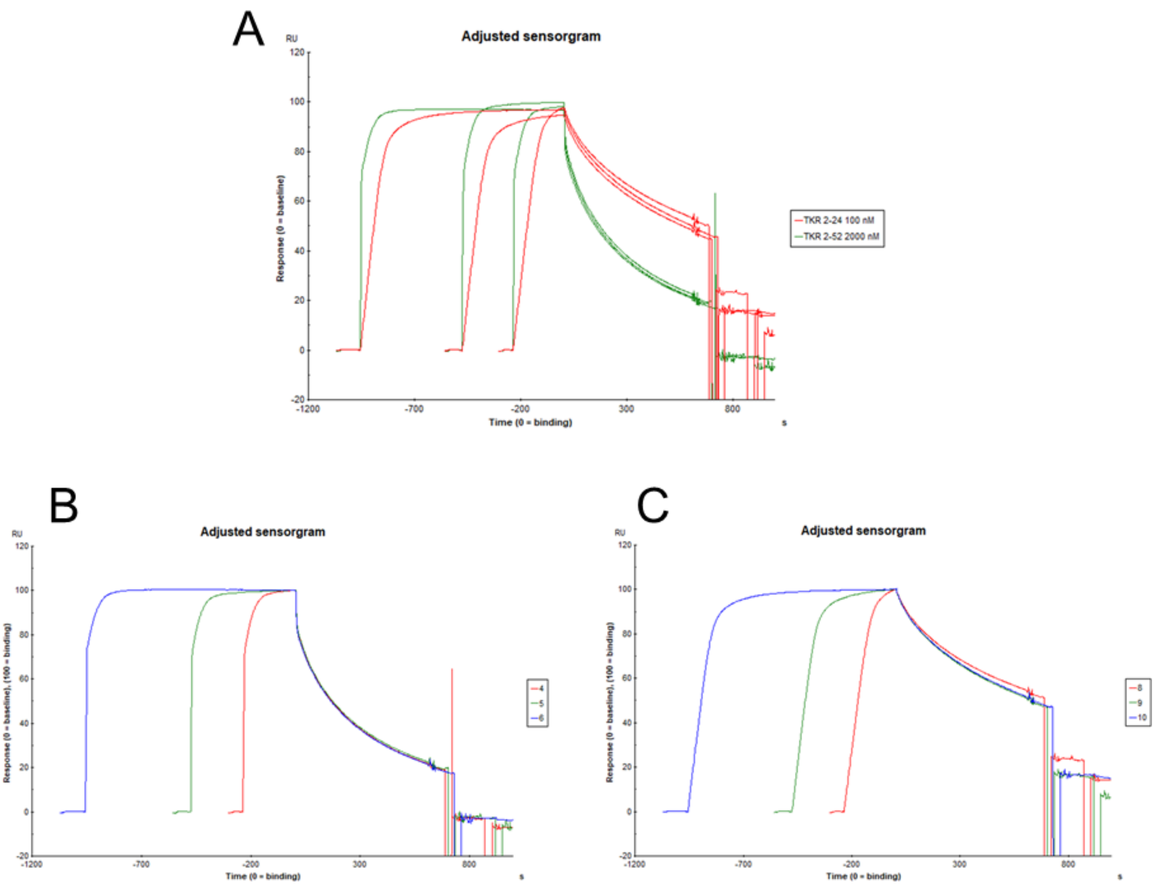

A
Adjusted sensorgram
RU
Response (0 = baseline)
Time (0 = binding)
s
TKR 2-24 100 nM
TKR 2-52 2000 nM
B
Adjusted sensorgram
RU
Response (0 = baseline), (100 = binding)
Time (0 = binding)
s
4
5
6
C
Adjusted sensorgram
RU
Response (0 = baseline), (100 = binding)
Time (0 = binding)
s
8
9
10

Figure S5: ECD (A) and UV (B) spectra of 1d and 1e

Stock solutions of bicyclic peptides **1d** and **1e** were prepared in a concentration of 0.125 mg/mL in 1:1 (v/v) mixture of 10 mM sodium phosphate pH 7.4 and CH_3CN or in a concentration of 0.083 mg/mL in a 1:1:1 (v/v/v) mixture of 10 mM sodium phosphate pH 7.4, CH_3CN and trifluoroethanol (TFE). The ECD spectra were measured in a quartz cuvette with an optical path length of 1 mm (Starna, USA) using a J-810 spectropolarimeter (Jasco, Japan). The conditions of the measurements were as follows: a spectral region of 200 (180)– 300 nm, a scanning speed of 20 nm min^{-1}, a response time of 8 s, a resolution of 1 nm, a bandwidth of 1 nm and a sensitivity of 100 mdeg. The final spectrum was obtained as an average of 4 accumulations. The spectra were corrected for a baseline by subtracting the spectra of the corresponding polypeptide-free solution. The ECD measurements were conducted at ambient temperature.

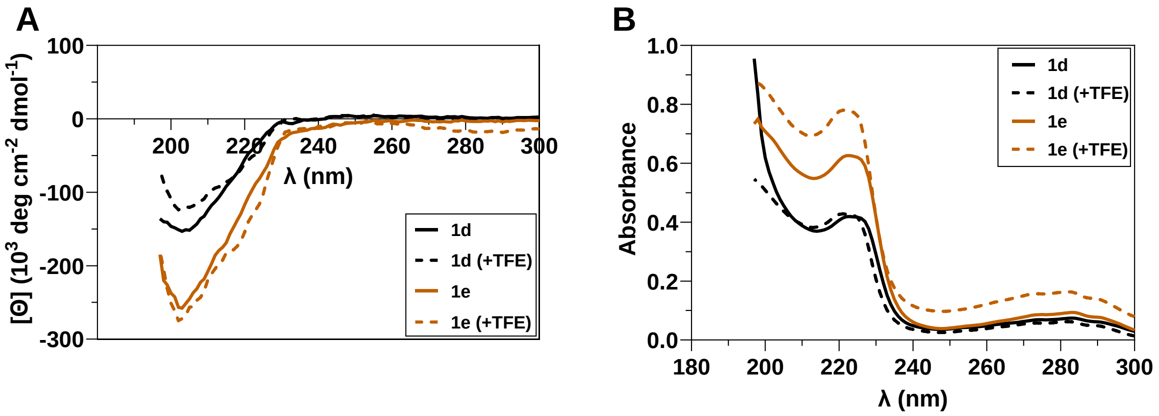

Figure S6: Temperature-dependent TOCSY correlation signals reveal distinct hydrogen bond environments

Selected regions depict correlations of A) Trp indole (N)H-1 and H-2, B) Ac-Cys $N_\alpha H$ and $C_\alpha H$, C) Ser $C_\beta H$ and $N_\alpha H$, and D) Thr $C_\gamma H$ and $N_\alpha H$. Especially at 25 and 40 °C, the signals due to NH reveal two distinct resonances, while at elevated temperatures only one signal remains. In each case, we ascribe the more downfield signals to NH···O=S(CH_3)$_2$ hydrogen bond associates, that dissociate upon heating.

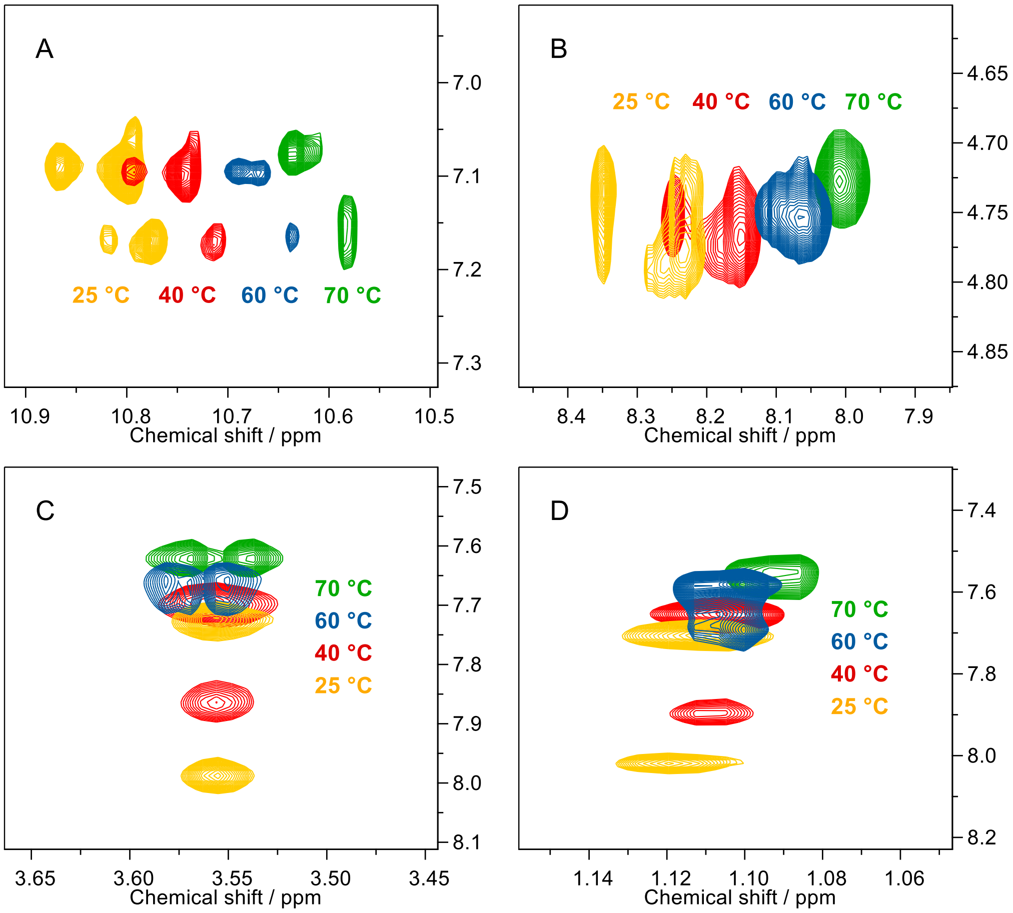

Figure S7: Observed ^{1}H,^{15}N-HSQC correlations at 25 °C

Although NH correlations associated with the peptide bonds could not be detected, the few observable correlations are yet interesting. Again, the (NH) hydrogen bond donor sites are discernable, especially for the indole NH, also mirroring the slightly distinct chemical environments from the ^{15}N perspective.

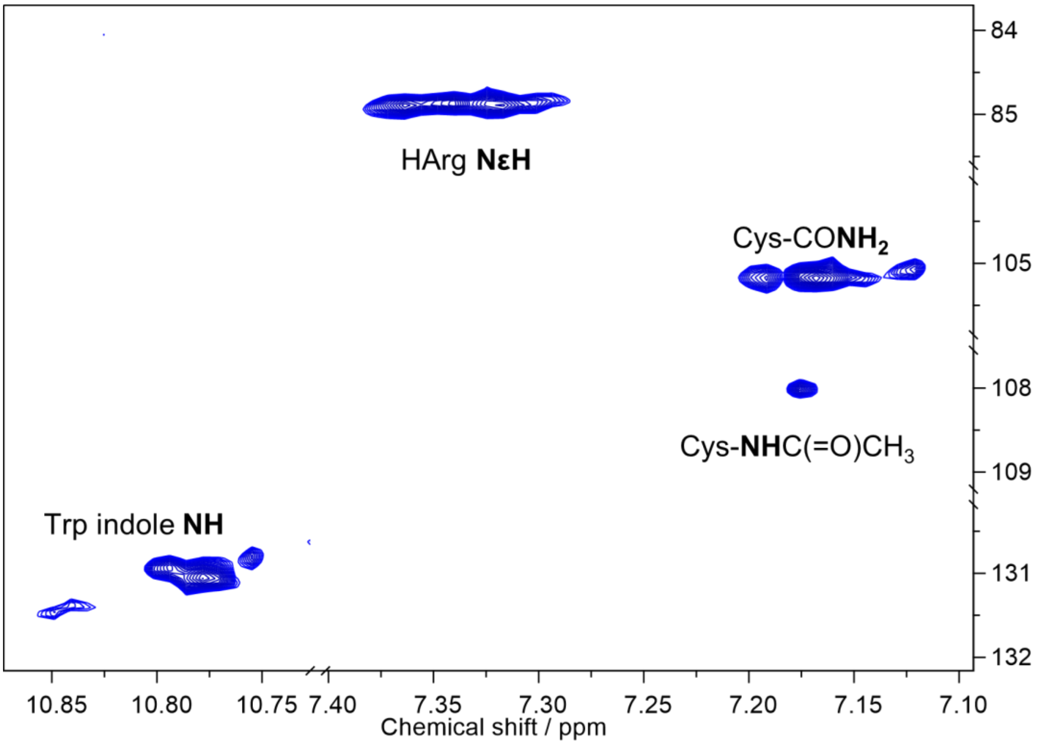

Figure S8: Side-by-side comparison of analytical radio-HPLC chromatograms after radiosynthesis of [^{64}Cu]Cu-1e (bearing Met^{4}) and [^{64}Cu]Cu-7 (bearing Cys(et)^{4})

The side-product formed upon ^{64}Cu-labeling of **1e**, i.e. **[^{64}Cu[Cu-2** (see main article for further explanations), is marked by an asterisk. No side-product was observed upon ^{64}Cu-labeling of **7**, which indicates a higher stability of the ethyl sulfhydryl group compared to the methyl sulfhydryl group.

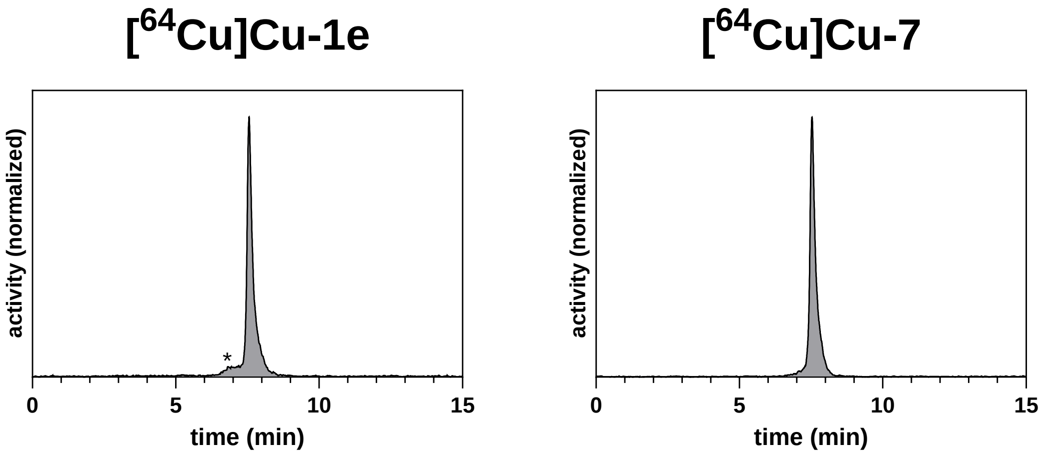

Figure S9: Exemplary analytical radio-HPLC chromatograms of the radiolabeled peptides upon incubation in diluted H_2O_2 solution

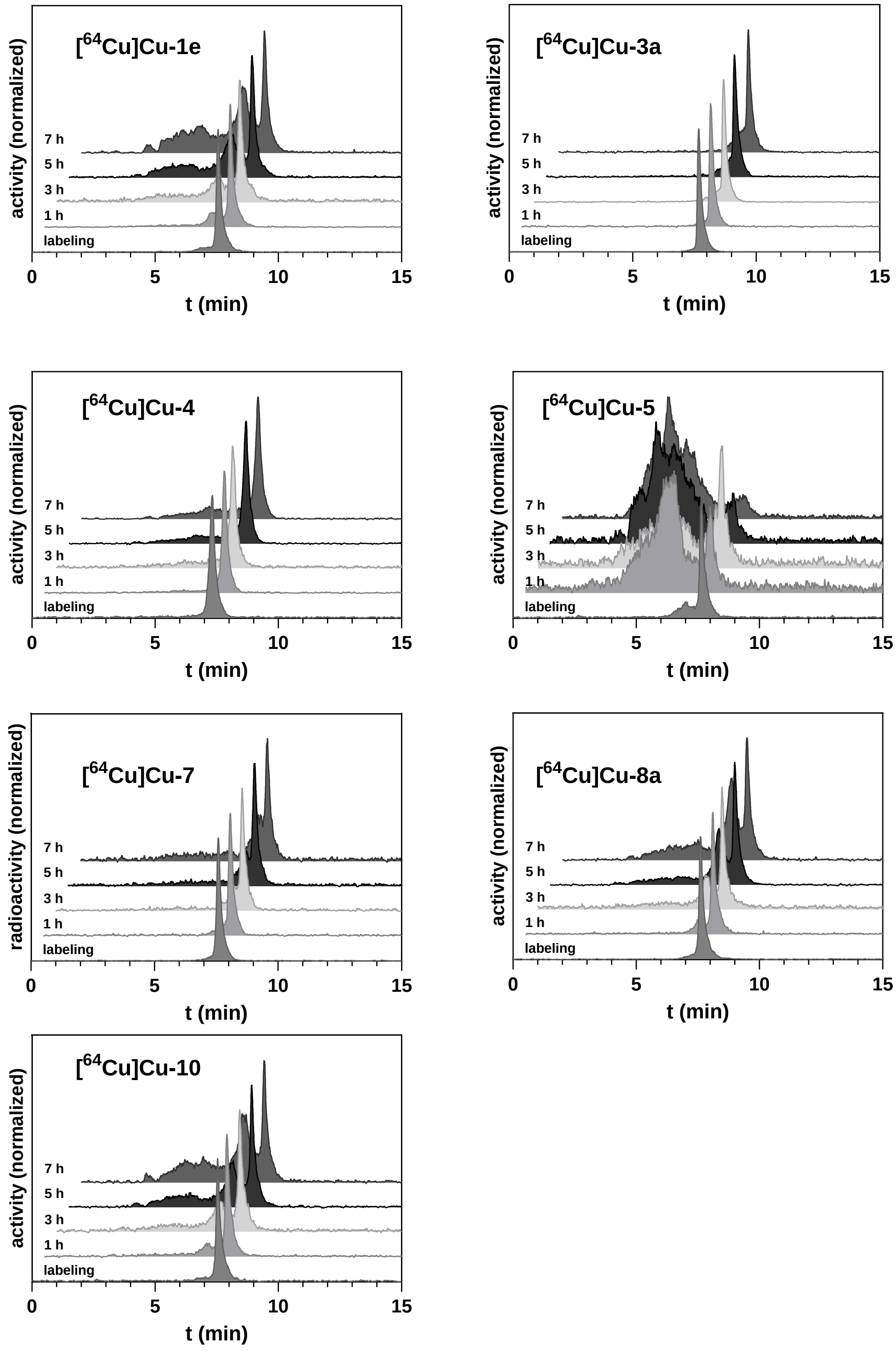

Figure S10: Exemplary radio-HPLC chromatograms of [^{64}Cu]Cu-1e, [^{64}Cu]Cu-3a, and [^{64}Cu]Cu-4 upon incubation *in vitro* in PBS and human plasma

Stability of **[^{64}Cu]Cu-1e** (**A**), **[^{64}Cu]Cu-3a** (**B**), and **[^{64}Cu]Cu-4** (**C**) in human plasma and stability of **[^{64}Cu]Cu-4** in PBS (**D**) after incubation at 37°C. Samples were taken after 1, 2, 4, and 24 h and were analyzed by radio-HPLC.

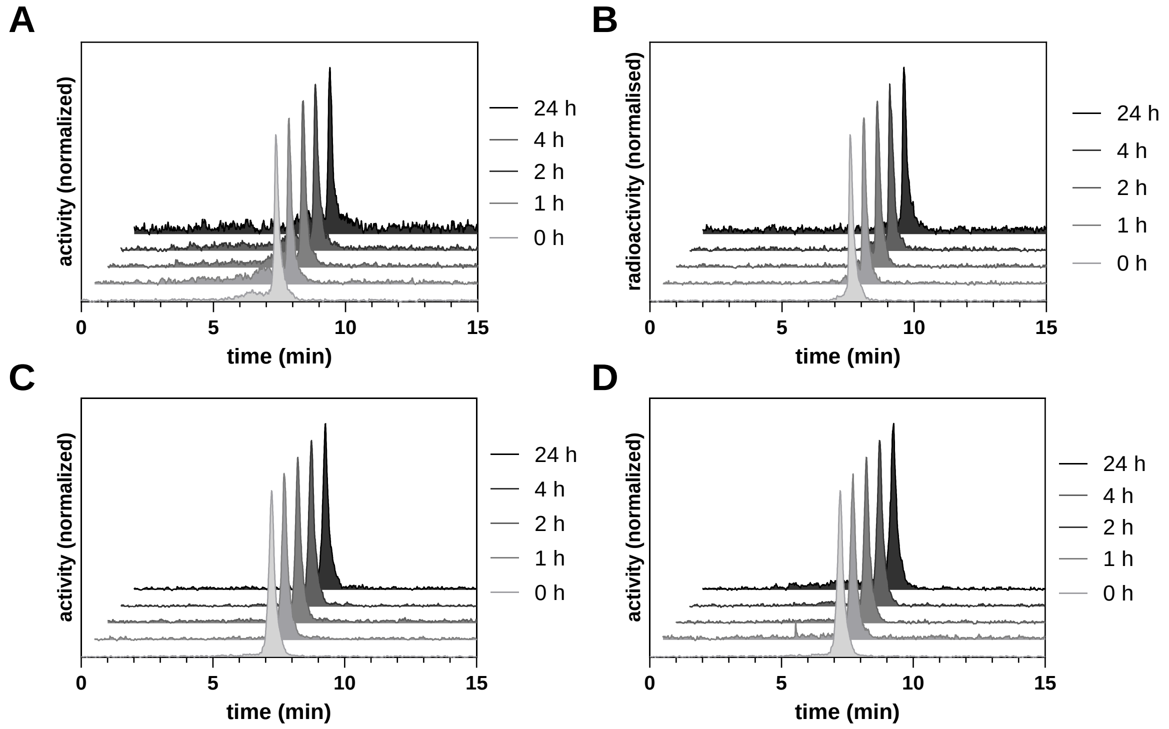

Figure S11: Assessment of plasma protein and HSA binding for [^{64}Cu]Cu-1e and [^{64}Cu]Cu-4 by ultrafiltration

A) Data of retained activity for **[^{64}Cu]Cu-1e** (1.3 µM) in PBS (10 mM, pH 7.4), HSA (33 g/L in PBS), and undiluted human plasma. **B**) Plot of "percentage of retained activity in filter" = f([HSA]) for **[^{64}Cu]Cu-4** with nonlinear regression according to the Morrison equation (total ligand concentration of 20 µM, achieved by addition of non-radioactive compound **4** after ^{64}Cu-labeling). For details on the experimental method and regression analysis see ref. [1].

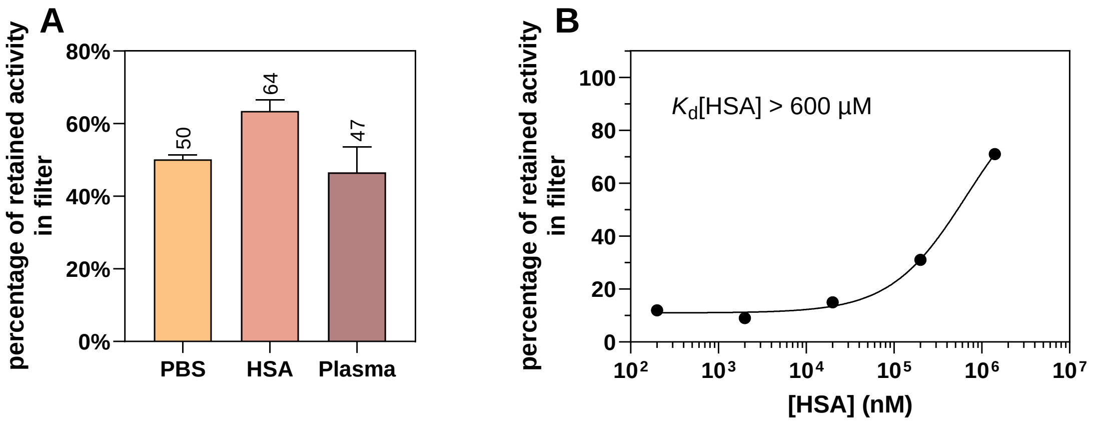

Figure S12: Isotype control for immunohistochemical staining of HT-1376 tumor slices

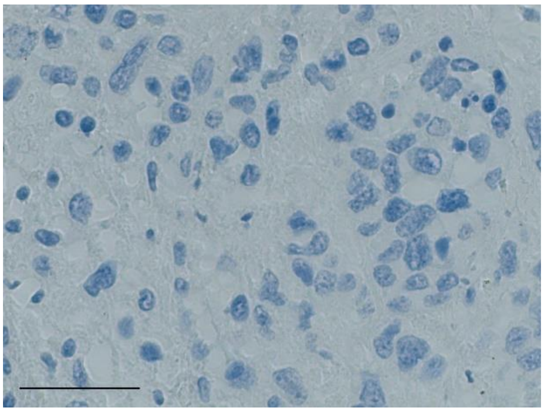

Figure S13: Time-dependent binding of [^{64}Cu]Cu-4 (100 nM) at 37°C (A) and 4°C (B) to 5637 cells

Total binding was obtained after PBS wash, while internalized fraction was obtained after washing with glycine buffer (pH 2.8). Data shown are mean values (±SD) of one experiment, which was performed in quadruplicate. Data for the surface-bound fraction were calculated from total and internalized fractions. Non-specific binding was assessed in the presence of 1 µM **1e**.

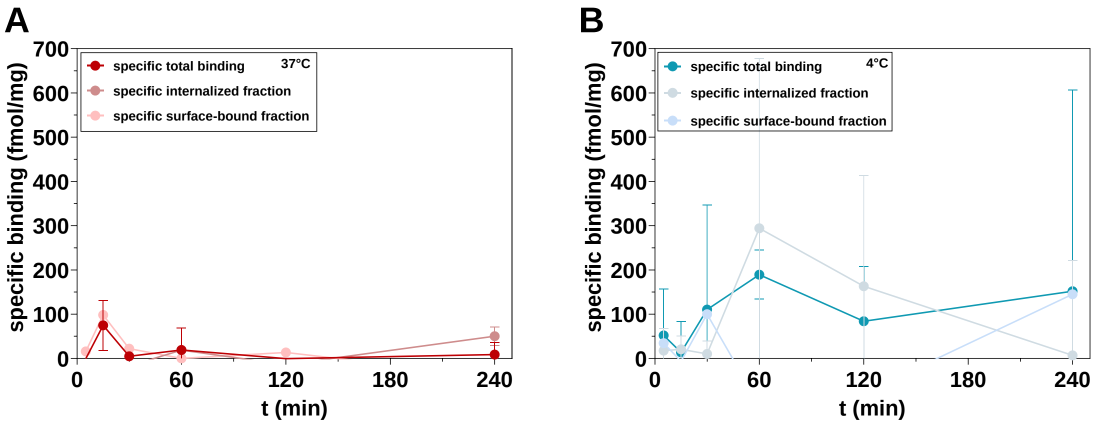

Figure S14: Saturation binding of [^{68}Ga]Ga-8d ([^{68}Ga]Ga-N188)

Data for total, nonspecific (in the presence of 1 µM **1e**) and calculated specific binding shown as black, grey, and blue circles, respectively. Regression analysis was performed as described in the experimental section. Data shown are mean values (±SD) of one experiment, which was performed in quintuplicate using intact HT-1376 cells.

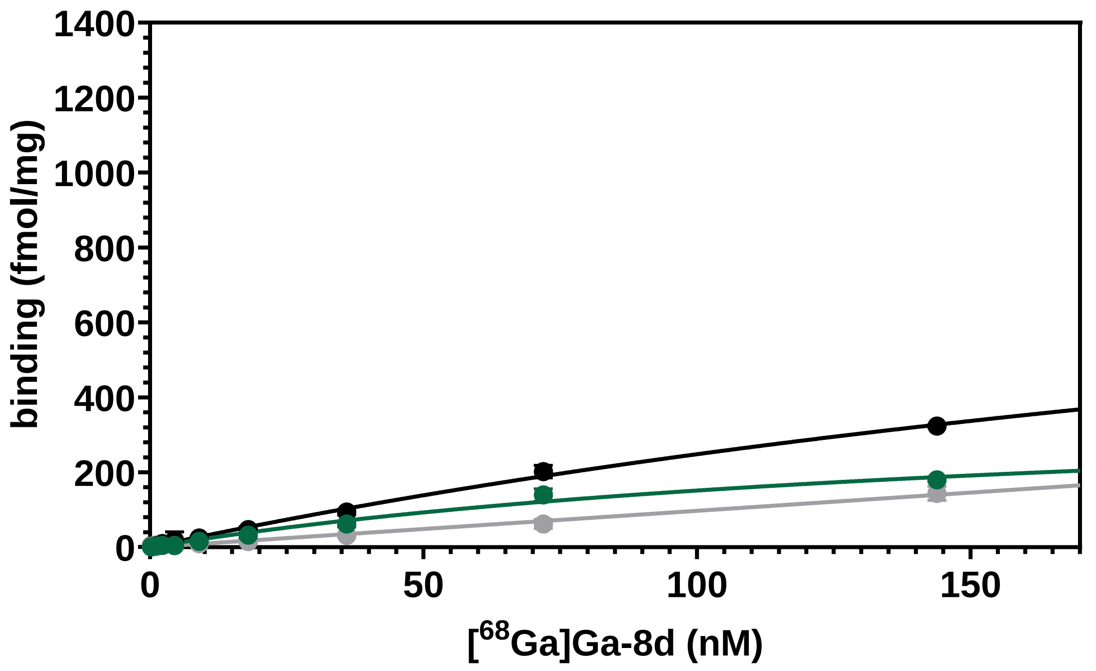

Figure S15: Time-activity curves of the ^{64}Cu- (top) and ^{68}Ga-labeled ligands (bottom) for muscle, kidney, liver, and urinary bladder uptake

Time-activity curves (SUVmean, decay-corrected, as a function of time up to 2 h) obtained by PET acquisition are depicted (0-2 h were dynamically recorded). From left to right: muscle, kidneys, liver, and urinary bladder. Color coding according to Figure 7 and Figure 8 in the main article.

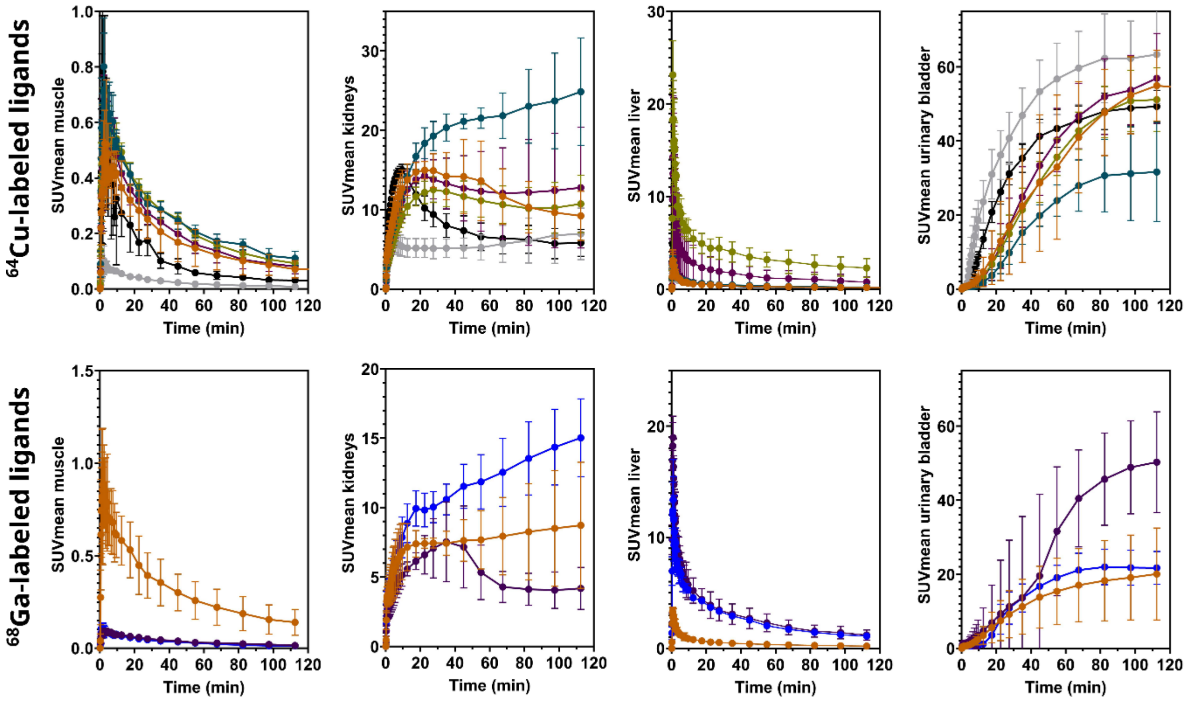

Figure S16: Tumor uptake of [^{64}Cu]Cu-1e and [^{64}Cu]Cu-3a under blocking conditions

A) PET/CT images at 1-2 h after intravenous injection of **[^{64}Cu]Cu-1e** and **[^{64}Cu]Cu-3a** co-injected with 75 nmol of **1d** (7-11 MBq/animal; 0.3–0.5 nmol/animal) in HT-1376 tumor bearing mice. Images are presented as maximum intensity projections and shown at common scale. Anatomical positions of tumor (tu), kidney (ki), and urinary bladder (ub) are shown. **B/C**) Time-activity curves (SUVmean, decay-corrected, as a function of time up to 2 h) for heart (**B**) and tumor (**C**) obtained by PET acquisition are depicted (0-2 h were dynamically recorded). **D**/**E**) Time-dependent SUVmean ratios up to 2 h *p.i.* for tumor-to-muscle (**D**) and tumor-to-heart (**E**). **F**) Time-dependent body retention in %ID up to 2 h p.i. **G**) Biodistribution at 1-2 h p.i. obtained by PET acquisition (br – brain, ht – heart, it – intestine, li – liver, mu – muscle, tu – tumor, ub – urinary bladder, ki – kidney). Data points in **B**-**G** are mean values (±SD) of a group of HT-1376 tumor-bearing mice (n=2). In **B**-**G**, data for **[^{64}Cu]Cu-1e** (dark orange) and **[^{64}Cu]Cu-3a** (dark blue) without co-administration of **1d** were included for comparison.

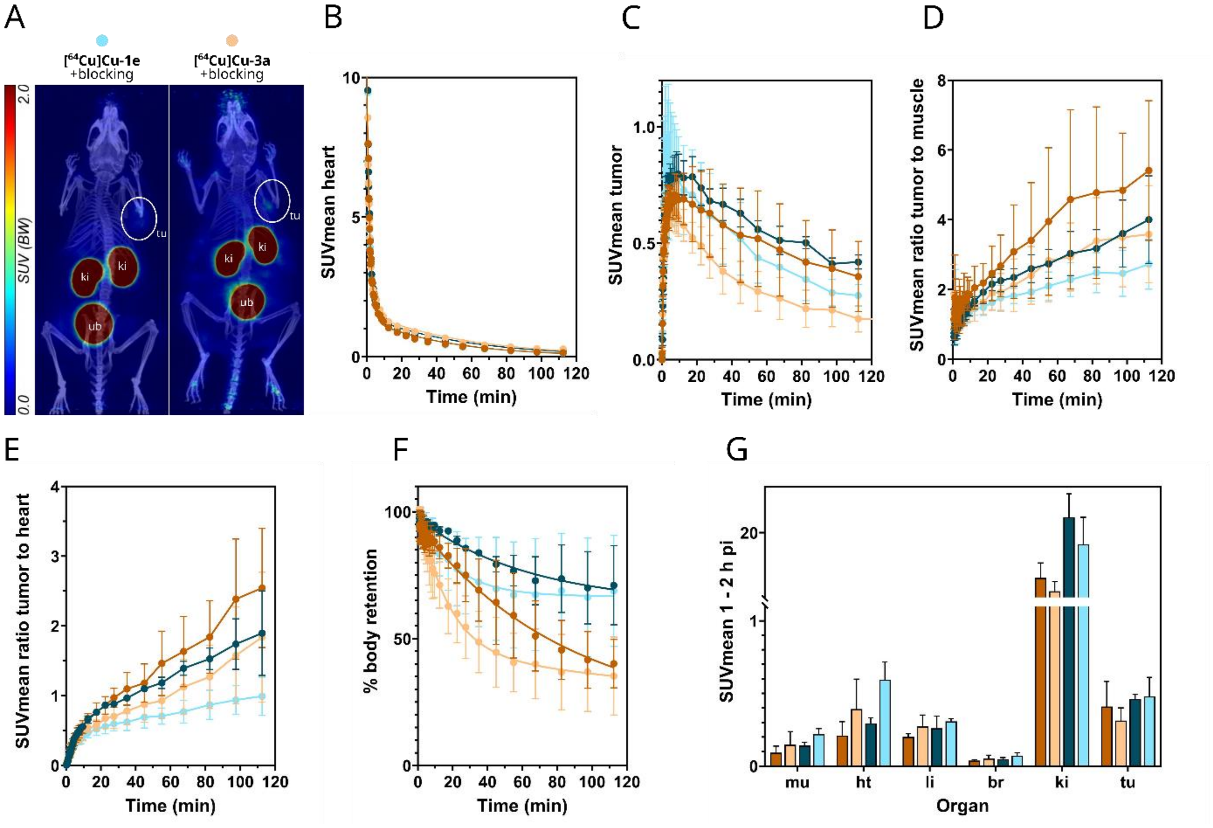

H

	[64Cu]Cu-1e<br>+ blocking	[64Cu]Cu-3a<br>+ blocking
**tumor SUVmean $_{1\text{-}2\ h\ p.i.}$**	0.22 ±0.06	0.33 ±0.07
	**SUVmean ratios (1-2 h p.i.)**	
**tumor to muscle**	3.35 ±1.08	2.48 ±0.49
**tumor to heart**	1.45 ±0.53	0.99 ±0.16
**tumor to liver**	1.11 ±0.13	1.55 ±0.43
**tumor to kidney**	0.03 ±0.01	0.02 ±0.00

Figure S17: PET images and derived data for [^{64}Cu]Cu-1e at 24 h *p.i.*

A) PET/CT images at 24 h after intravenous injection of **[^{64}Cu]Cu-1e** alone or co-injected with 75 nmol of **1d** (blocking) in HT-1376 tumor bearing mice and of **[^{64}Cu]Cu-1e** in 5637 tumor bearing mice. 7-11 MBq/animal (0.3–0.5 nmol/animal) were injected. Images are presented as maximum intensity projections and shown at common scale. Anatomical positions of tumor (tu), kidney (ki), and urinary bladder (ub) are shown. **B**) Time-activity curves (SUVmean, decay-corrected, as a function of time up to 2 h) for the tumor obtained by PET acquisition are depicted (0-2 h were dynamically recorded, 24 h in static mode). **C**/**D**) Time-dependent SUVmean ratios up to 2 h p.i. for tumor-to-muscle (**C**) and tumor-to-heart (**D**) up to 24 h *p.i.* **E**) Summary of values for SUVmean and SUVmean ratios at 24 h *p.i.*

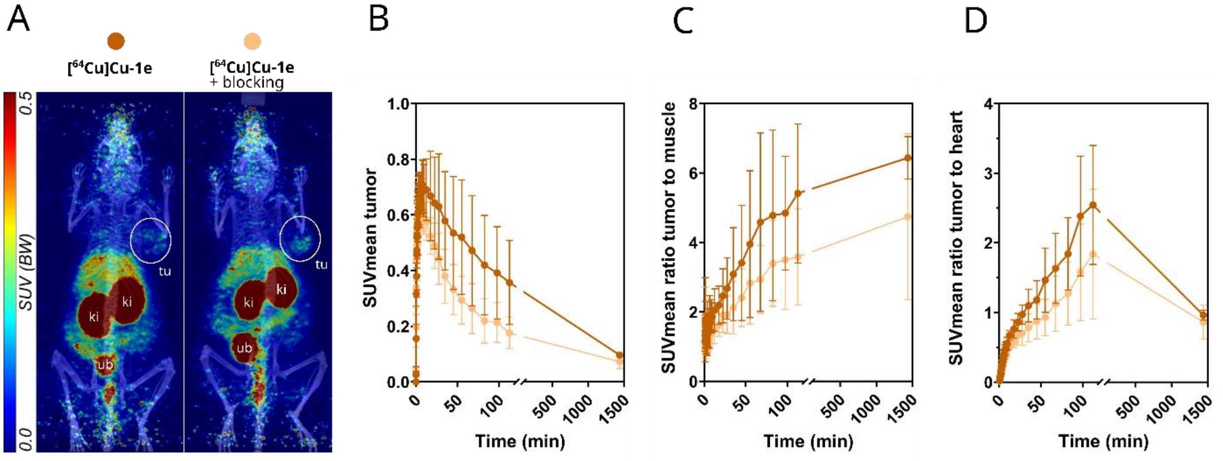

E

	[^{64}Cu]Cu-1e	[^{64}Cu]Cu-1e + blocking
**tumor SUV $_{24h}$**	0.10 ±0.01	0.07 ±0.02
	**SUVmean ratios (24 h p.i.)**	
**tumor to muscle**	6.43 ±0.43	4.74 ±1.69
**tumor to heart**	0.96 ±0.05	0.86 ±0.17
**tumor to liver**	0.45 ±0.01	0.39 ±0.08
**tumor to kidney**	0.05 ±0.00	0.05 ±0.01

Figure S18: PET images and derived data for [^{64}Cu]Cu-4 and [^{64}Cu]Cu-8b at low molar amounts of total radioligand

A) PET/CT images at 1-2 h after intravenous injection of **[^{64}Cu]Cu-4** and **[^{64}Cu]Cu-8a** at a high molar activity of 200 MBq/nmol (2 MBq/animal, 0.01 nmol/animal) in HT-1376 tumor bearing mice. **B**) Time-activity curves (SUVmean, decay-corrected, as a function of time up to 2 h) for the tumor obtained by PET acquisition are depicted (0-2 h were dynamically recorded). In **B**, data for **[^{64}Cu]Cu-4** (dark purple) and **[^{64}Cu]Cu-8a** (dark green) at lower molar activity (25 MBq/nmol) were included for comparison. **C**) Summary of values for SUVmean and SUVmean ratios at 1-2 h *p.i.*

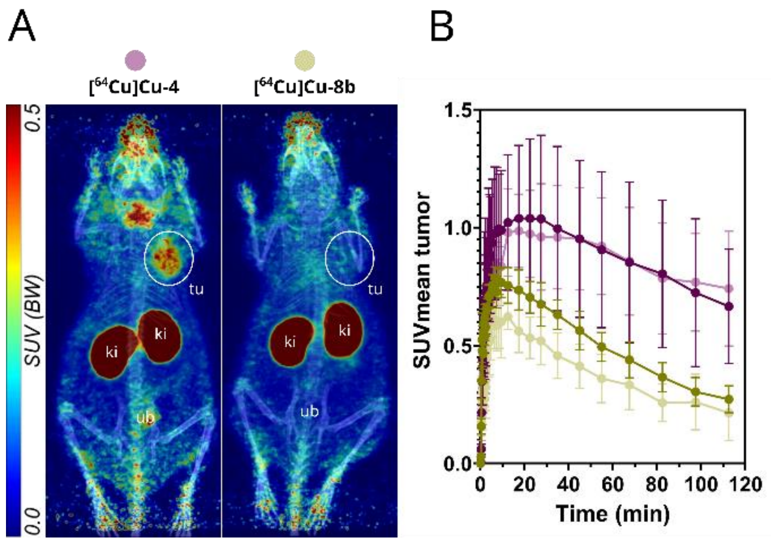

C

	[^{64}Cu]Cu-4<br>high Am	[^{64}Cu]Cu-8c<br>high Am
**tumor SUV $_{1\text{-}2h}$**	0.79 ±0.19	0.27 ±0.09
**SUVmean ratios (1-2 h p.i.)**		
**tumor to muscle**	3.49 ±0.71	2.69 ±0.82
**tumor to heart**	1.62 ±0.37	1.42 ±0.53
**tumor to liver**	2.35 ±0.65	1.60 ±0.47
**tumor to kidney**	0.03 ±0.01	0.02 ±0.01

Figure S19: Side-by-side comparison of time-activity curves of [^{64}Cu]Cu-1e (bearing Met^4) and [^{64}Cu]Cu-2 (bearing Met(O)^4)

A) PET/CT image at 1-2 h after intravenous injection of **[^{64}Cu]Cu-2** (8 MBq/animal, 0.4 nmol/animal) in HT-1376 tumor bearing mice. **B**) Time-activity curves (SUVmean, decay-corrected, as a function of time up to 2 h) for the tumor obtained by PET acquisition are depicted (0-2 h were dynamically recorded). **C**/**D**) Time-dependent SUVmean ratios up to 2 h *p.i.* for tumor-to-muscle (**D**) and tumor-to-heart (**D**) up to 2 h *p.i.* In **B**-**D**, data for **[^{64}Cu]Cu-1e** (dark orange) were included for comparison.

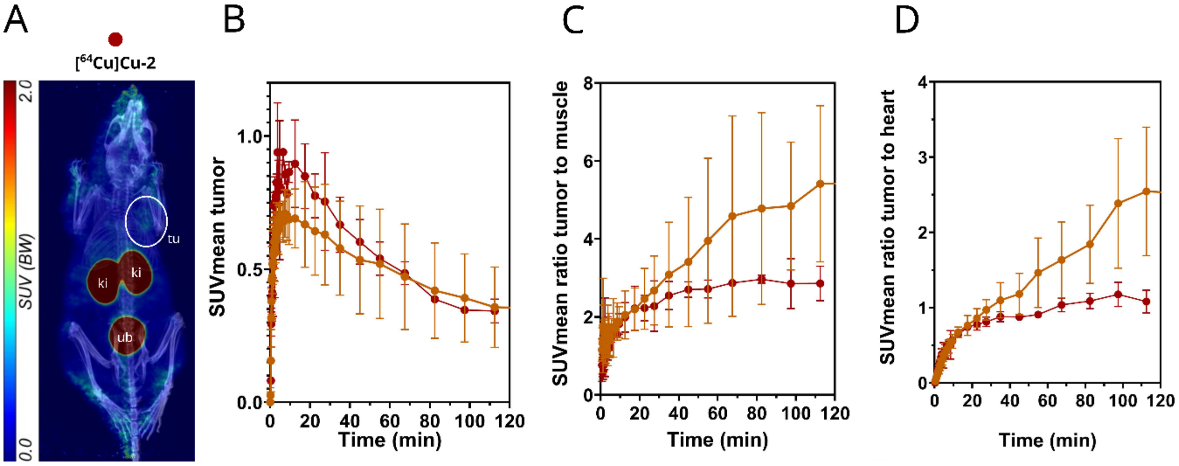

E

	[^{64}Cu]Cu-2
**tumor SUVmean $_{1\text{-}2\ h\ p.i.}$**	0.38 ±0.41
	**SUVmean ratios (1-2 h p.i.)**
**tumor to muscle**	2.89 ±0.18
**tumor to heart**	1.09 ±0.09
**tumor to liver**	0.94 ±0.06
**tumor to kidney**	0.02 ±0.00

Scheme S1: General synthesis of the bicyclic peptides

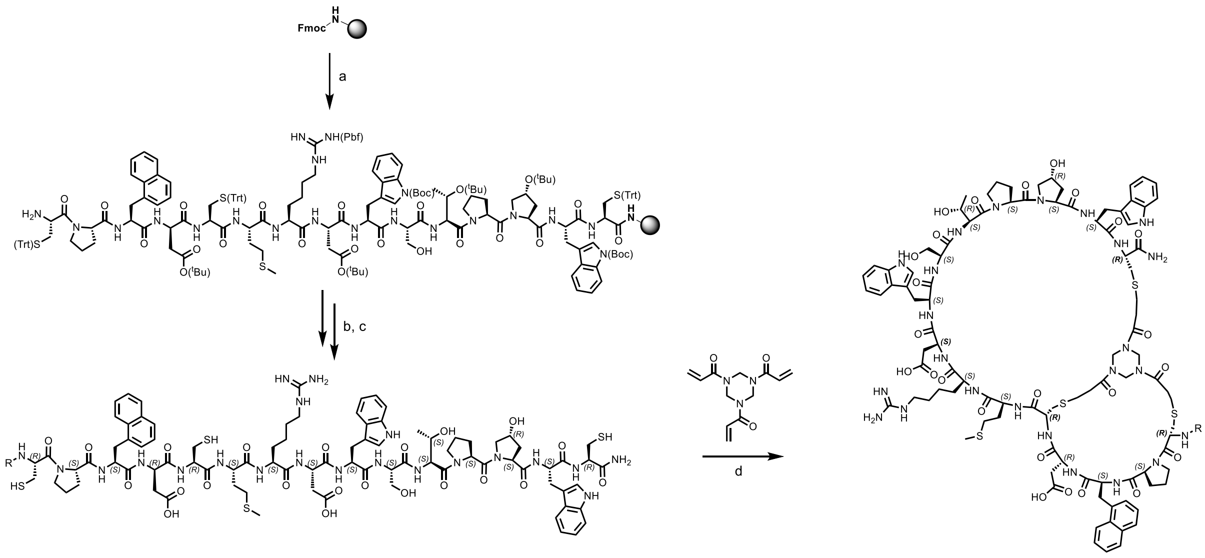

a) Fmoc/*t*Bu-based solid-phase peptide synthesis using an automated microwave peptide synthesizer (Biotage Initiator+ Alstra) with standard protocols for coupling (HATU/DIPEA) and Fmoc removal (20% piperidine in DMF); **b$_1$**) acetic anhydride, DIPEA, CH_2Cl_2, 2 h or **b$_2$**) R-COOH, HATU, DIPEA, DMF, 1-18 h depending on the particular carboxylic acid derivative; **c**) TFA/H_2O/TIPS (95:2.5:2.5, v/v/v), 40°C, 4 h; **d**) 1,3,5-Triacryloylhexahydro-1,3,5-triazine (TATA), NH_4HCO_3, TCEP, 1 h.

Table S1: IC_{50} values and Hill coefficients (n) determined with the FA based competitive binding assay

Data shown are mean values of two separate experiments (each performed in duplicate) with estimated confidence interval (68.3%) for the IC_{50} values in brackets.

compound	IC_{50} (nM)	n
**1c**	51.8 (50.8-52.8)	2.12
**1d**	34.7 (33.8-35.6)	2.37
**1e**	35.6 (31.5-40.6)	1.52
**^{nat}Cu-1e**	23.7 (21.9-25.7)	1.78
**^{nat}Ga-1e**	203 (202-203)	2.05
**1f**	177 (138-260)	1.18
**2**	568 (550-588)	1.07
**3a**	41.6 (38.1-45.6)	1.30
**^{nat}Cu-3a**	45.4 (43.4-47.7)	1.51
**3b**	31.5 (29.7-33.4)	1.80
**4**	54.6 (51.7-57.9)	1.72
**5**	48.7 (45.1-52.7)	1.69
**6**	379 (339-431)	1.42
**7**	151 (123-208)	1.93
**8a**	479 (323-915)	0.99
**8c**	3290 (2910-3850)	1.23
**8d**	4219 (3955-4496)	2.53
**9**	270 (260-281)	1.57
**10**	32.1 (31.0-33.3)	2.05

Table S2: Preliminary assignments of ^{1}H NMR chemical shifts for 1d at 70°C

Pos.	AA	$C_\alpha H$	$C_\beta H_2$	$C_\gamma H_2$	$C_\delta H_2$	$C_\varepsilon H_2$	$N_\alpha H$	Other
	Ac-Cys	4.748	2.983/2.746	-	-	-	8.029	1.850 (CH_3 of Ac)
1	Pro	4.109	1.764/1.952	1.466/1.764	3.612	-	-	-
2	1Nal	4.47	3.699/3.298	-	-	-	-	8.116 (H-8), 7.913 (H-5), 7.787 (H-4), 7.566 (H-7), 7.416 (H-3), 7.361 (H-2)
3	D-Asp	4.520	2.654/2.540	-	-	-	8.013	-
	Cys	4.630<br>4.591	2.729/2.584<br>3.191/3.016	-	-	-		-
4	Met	4.395	1.842/1.942	2.863/2.42	-	-	7.982	1.86 (CH_3)
5	hArg	4.199	1.643/1.508	1.260	1.427	3.047	7.580	7.282 (N_eH)
6	Asp	4.520	2.728/2.596	-	-	-	7.888	-
7	Trp	4.45	3.009	-	-	-	7.865	7.514 (H-4), 7.324 (H-7), 7.145/7.096 (H-2), 7.052 (H-6), 6.972 (H-5), ≈10.6 (indole NH)
8	Ser	4.286	3.573	-	-	-	7.669	
9	Thr	4.419	3.902 ($C_\beta H$)	1.114 ($C_\gamma H_3$)	-	-	7.575	-
10	Pro	4.513	1.975/1.811	1.811/1.521	3.785/3.552	-	-	-
11	Hyp	4.411	2.740/2.910	n.d.	3.581	-	-	-
12	Trp	4.442	3.194/3.125	-	-	-	7.841	7.514 (H-4), 7.324 (H-7), 7.145/7.096 (H-2), 7.052 (H-6), 6.972 (H-5); ≈10.6 (indole NH)
	Cys	4.630<br>4.591	2.729/2.584<br>3.191/3.016	-	-	-	7.575	-
	TATA	-	-	-	-	-	-	2.74 (6×CH_2)<br>5.33 (3×NCH_2)

NMR spectra of 1d

^{1}H-NMR spectra of 1d at various temperatures in DMSO-d_6

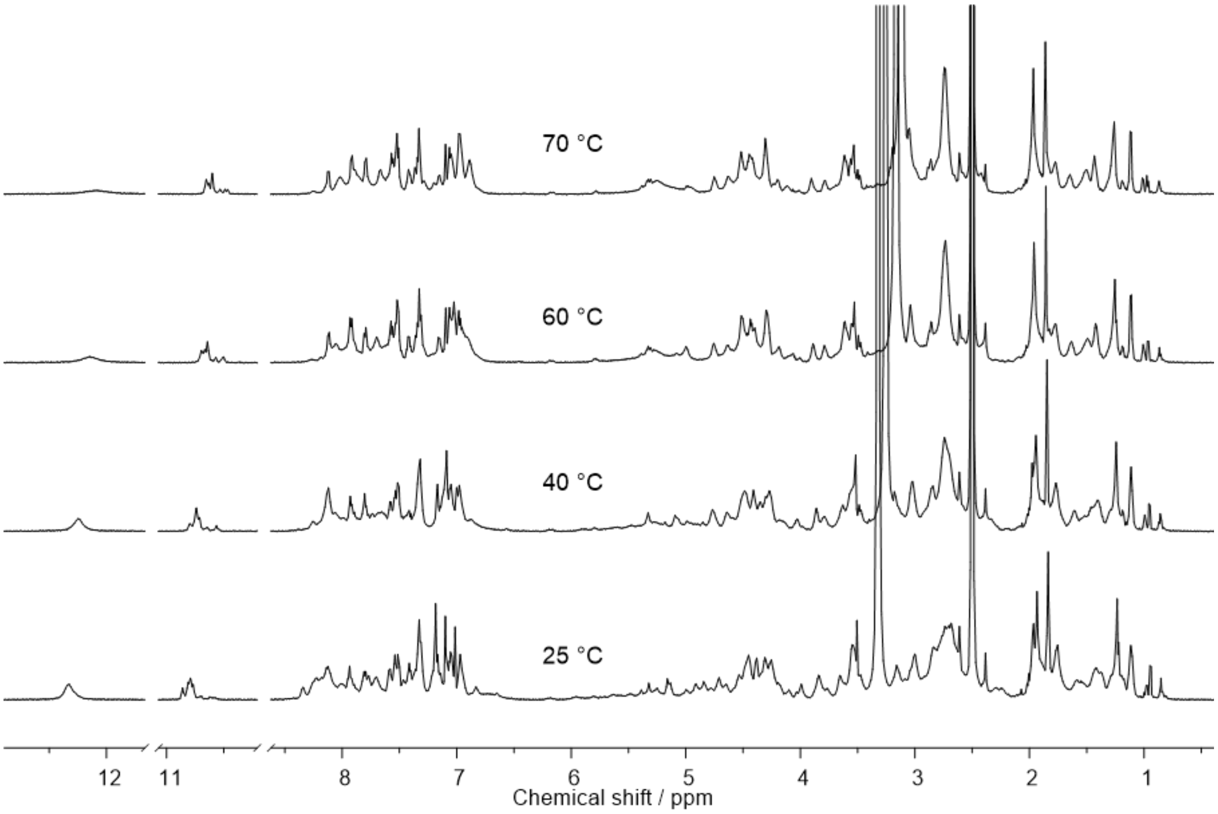

COSY spectrum of 1d at 25 °C in DMSO-d_6

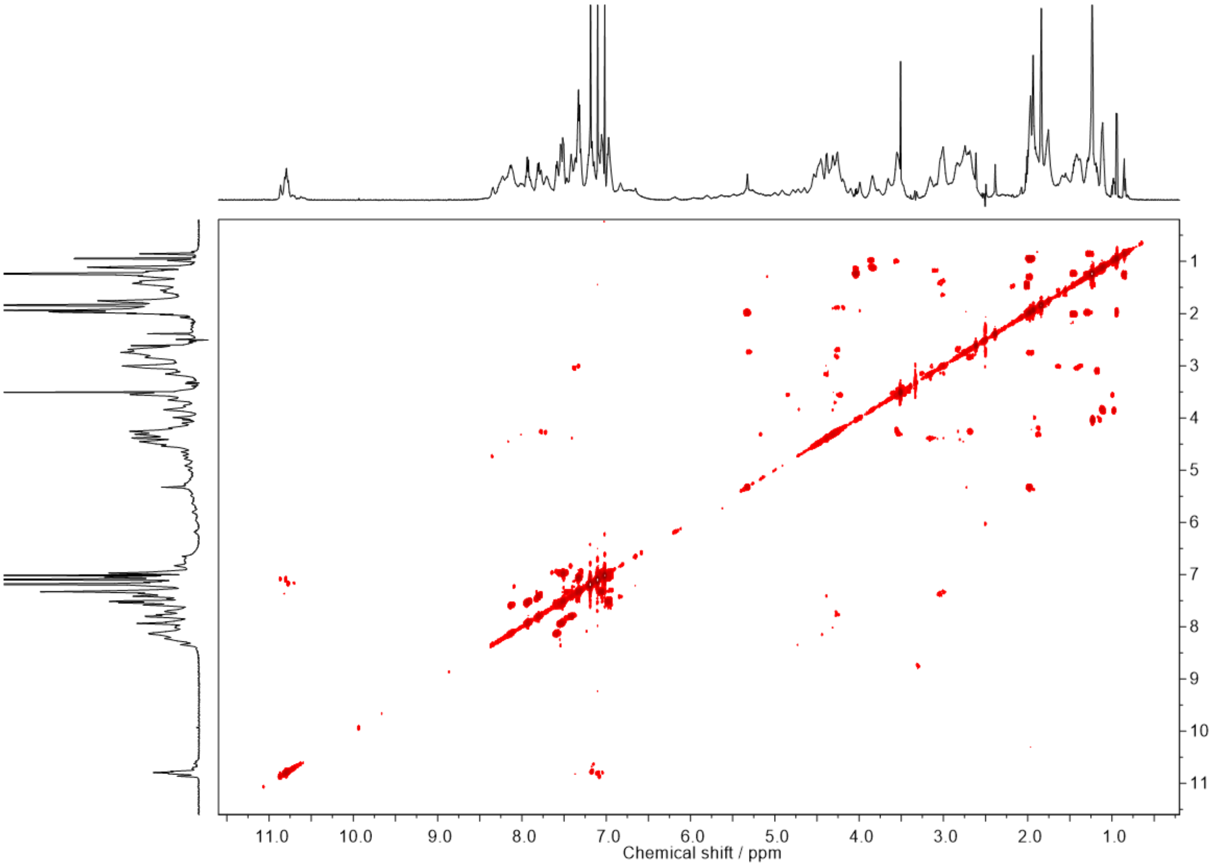

TOCSY spectrum of 1d at 25 °C in DMSO-d_6

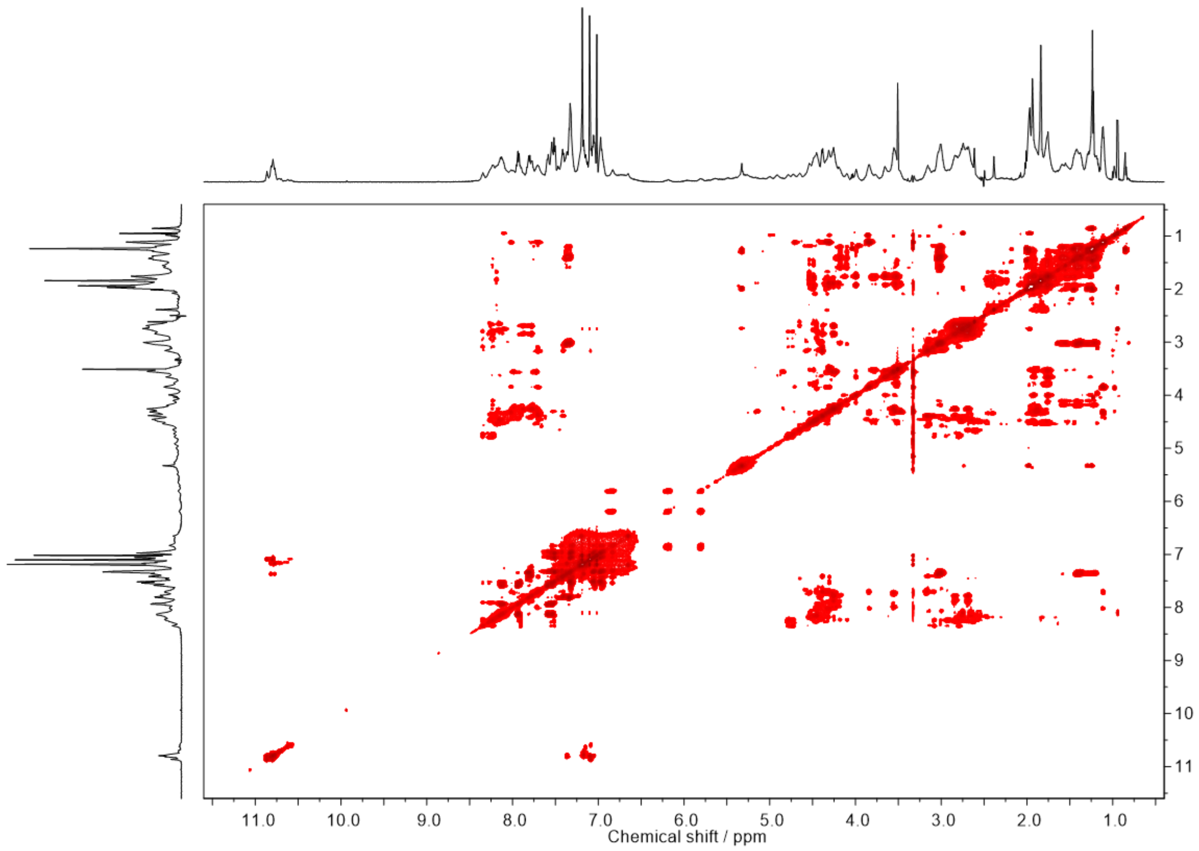

ROESY spectrum of 1d at 25 °C in DMSO-d_6

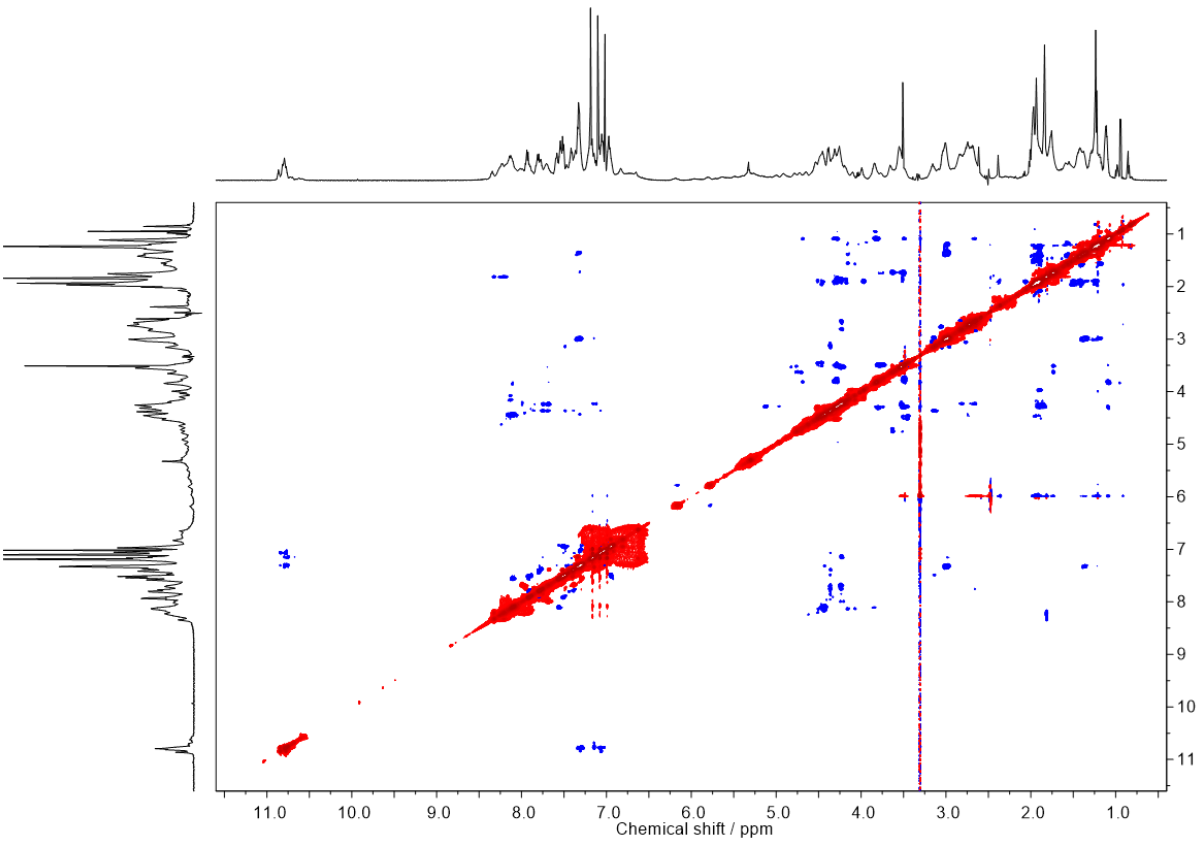

HSQC spectrum of 1d at 25 °C in DMSO-d_6

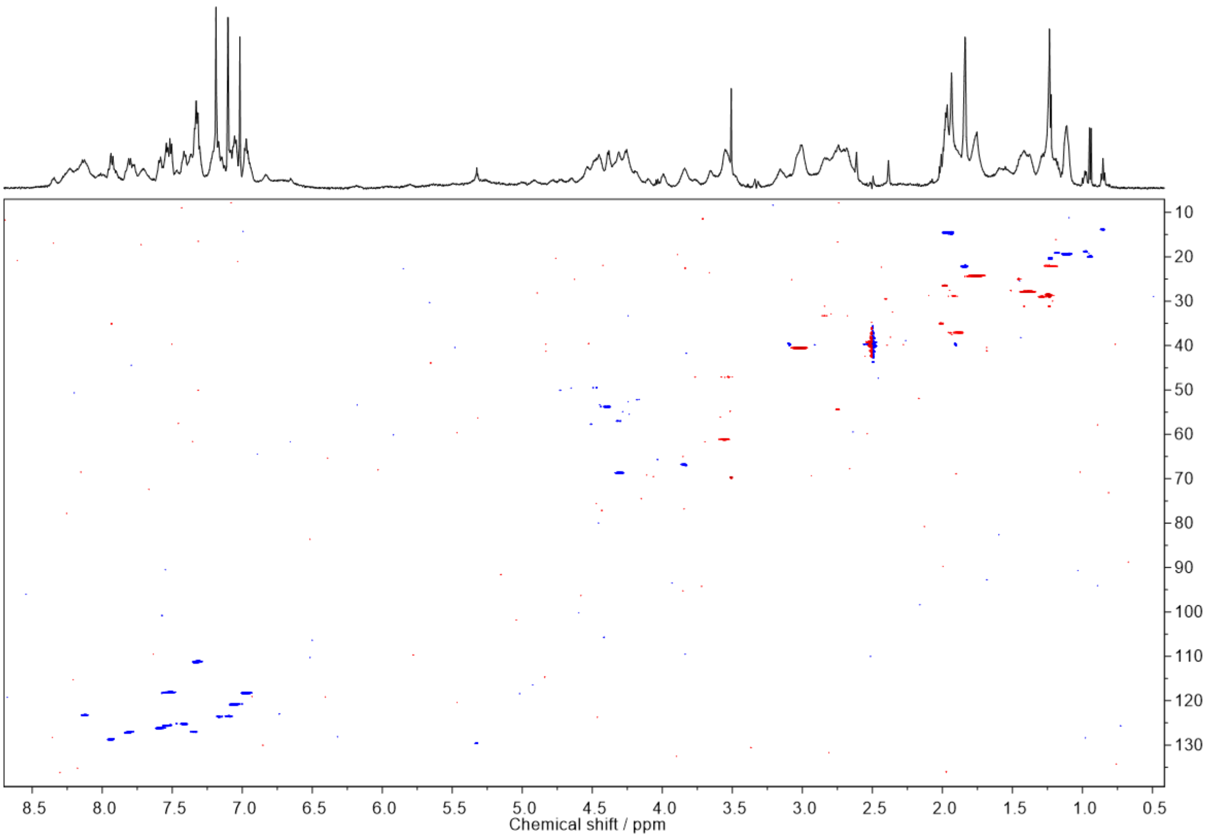

HMBC spectrum of 1d at 25 °C in DMSO-d_6

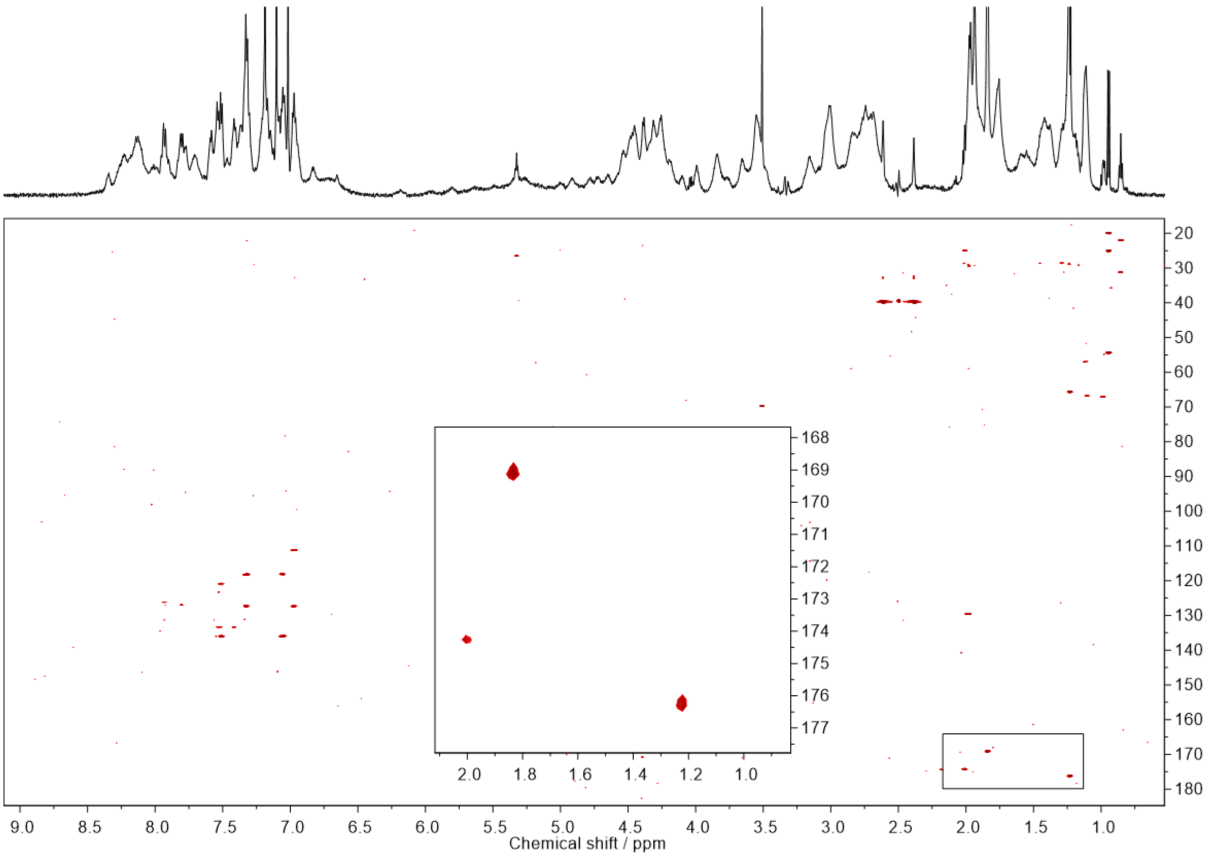

^{1}H-NMR spectrum at 70°C in DMSO-d_6

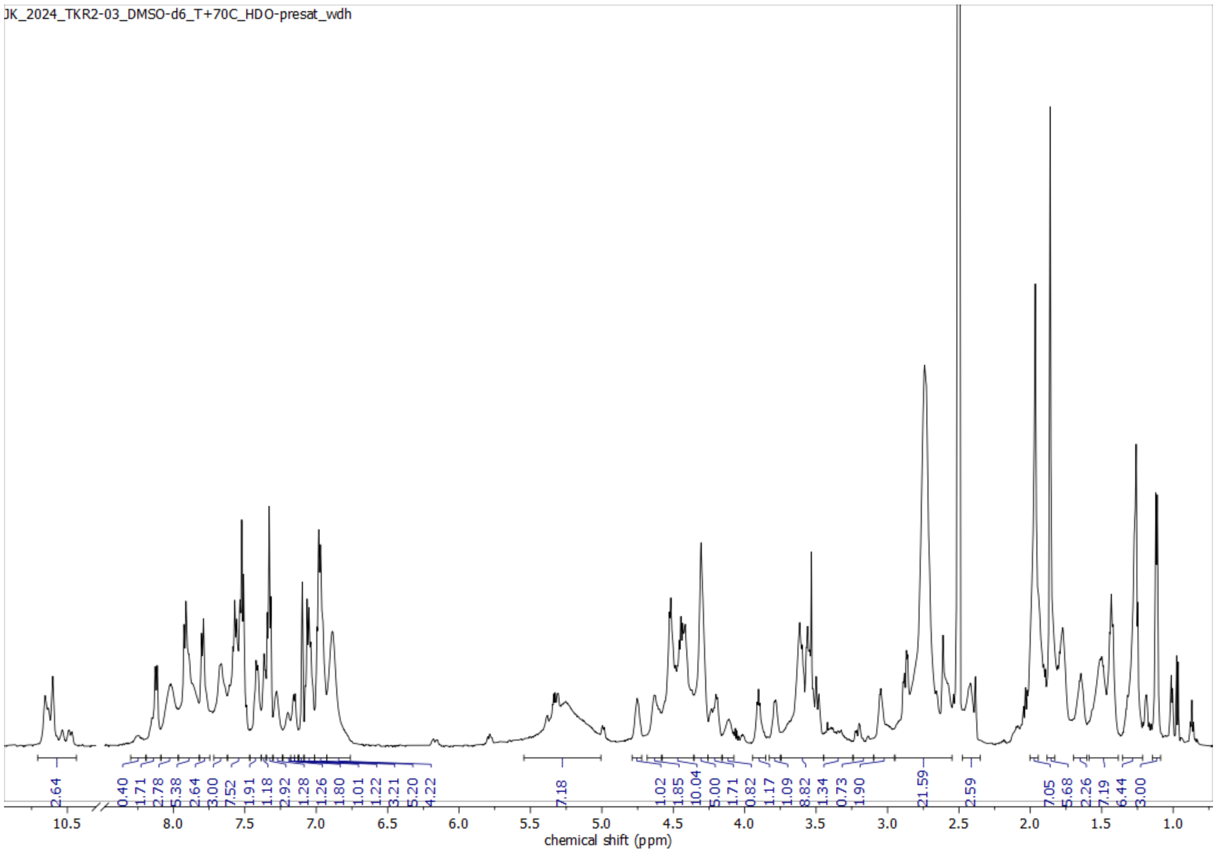

COSY spectrum of 1d at 70°C in DMSO-*d*$_6$

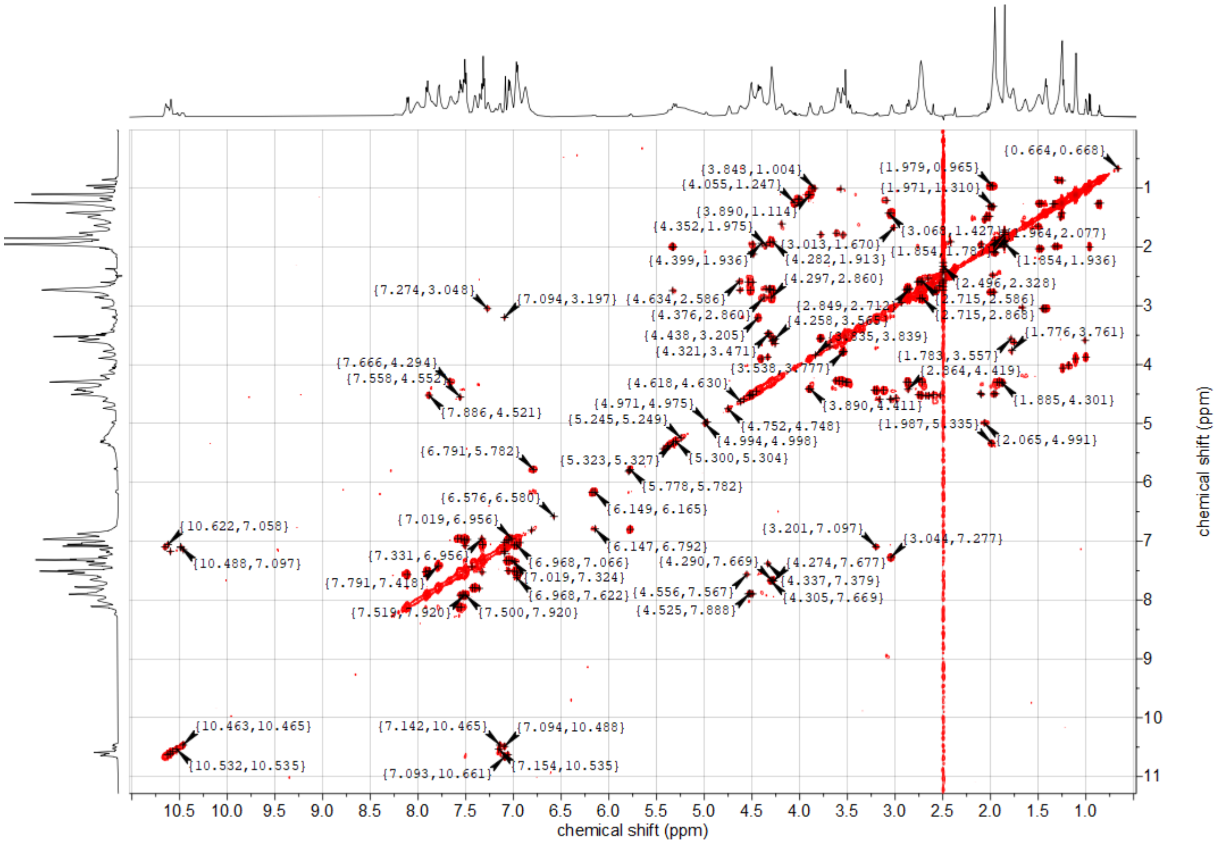

TOCSY spectrum of 1d at 70°C in DMSO-d_6

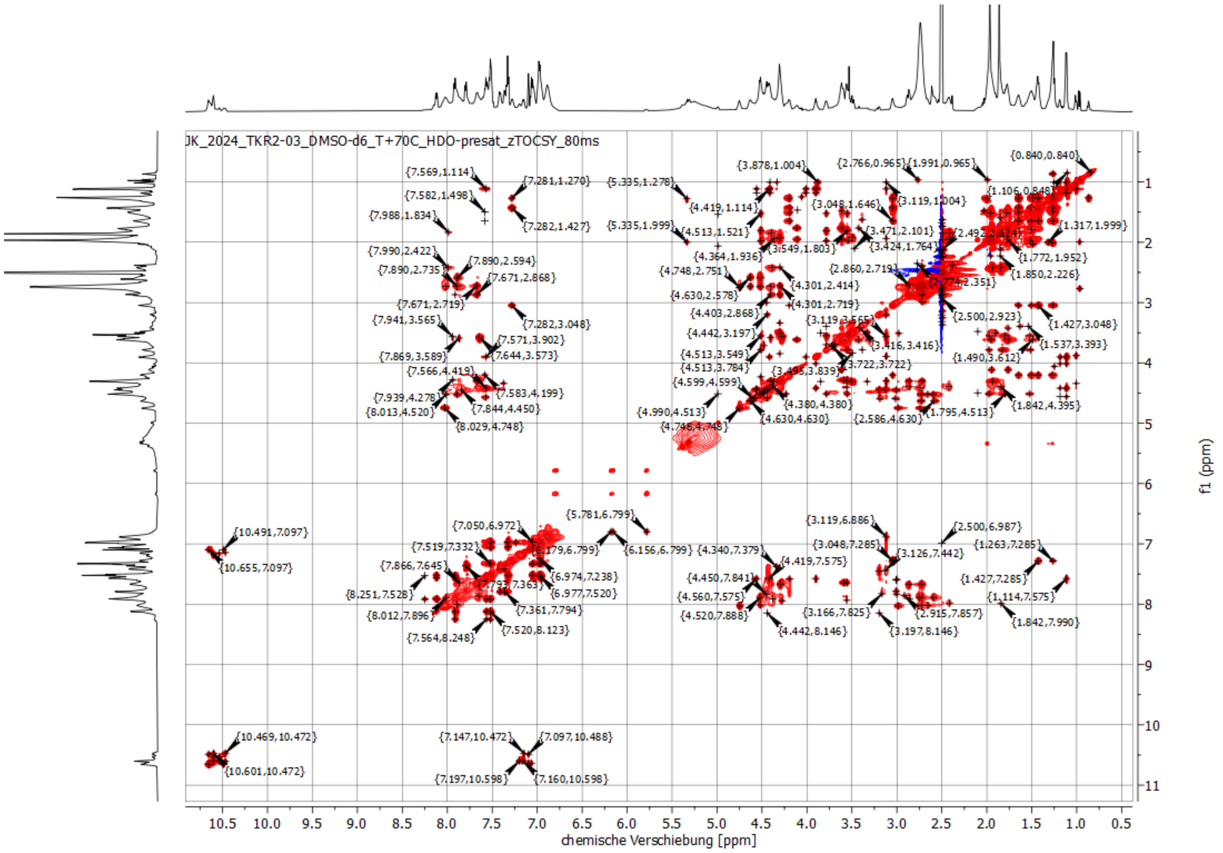

ROESY spectrum of 1d at 70°C in DMSO-d_6

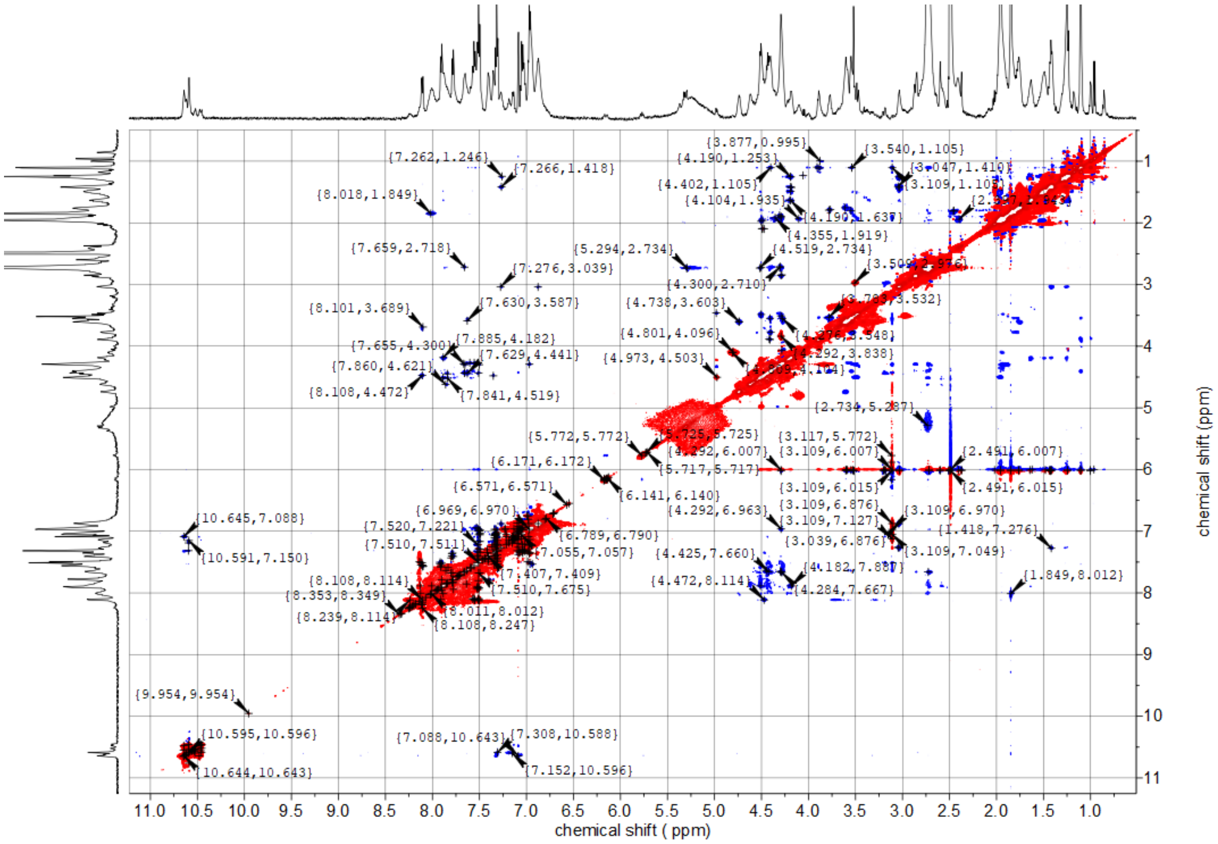

HSQC spectrum of 1d at 70 °C in DMSO-d_6

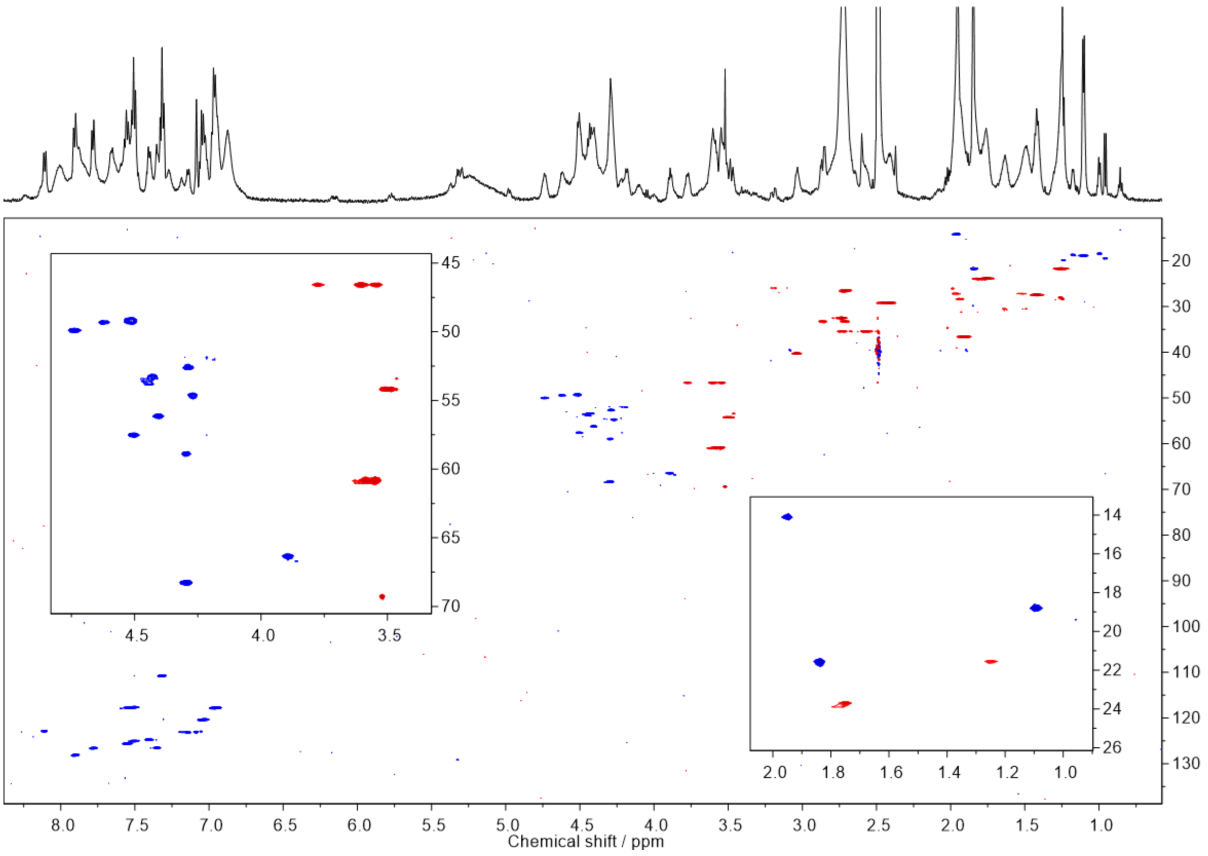

HMBC spectrum of 1d at 70 °C in DMSO-d_6

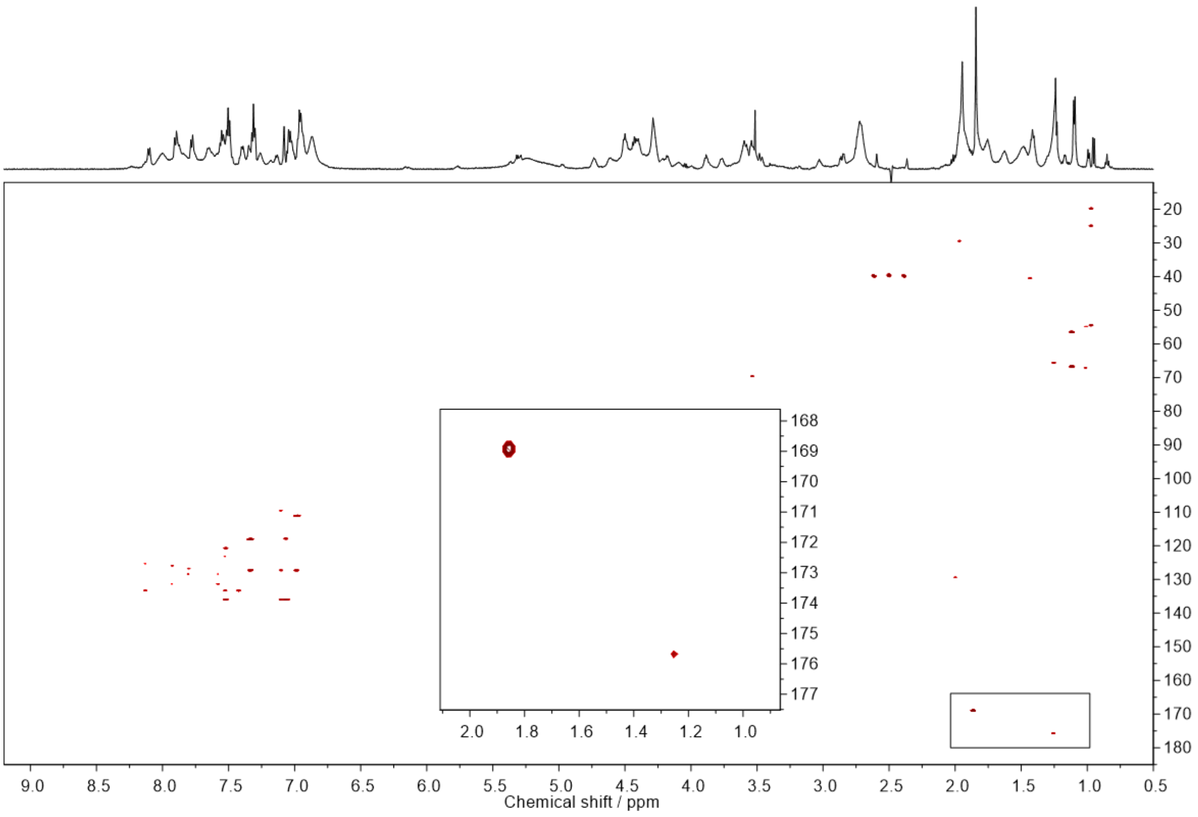

Table S3: CHI $IAM_{7.4}$ values for selected peptides

compound	CHI $IAM_{7.4}$
**1e**	19.9
**^{nat}Cu-1e**	23.4
**3a**	20.6
**^{nat}Cu-3a**	23.8
**4**	20.2
**5**	19.5
**8a**	20.3
**10**	19.9

Table S4: Blood kinetic parameters for the ^{64}Cu-labeled ligands

Parameters were derived from non-linear regression of the TAC for heart uptake according to the model of two-phase decay.

	**[^{64}Cu]Cu-1e**	**[^{64}Cu]Cu-3a**	**[^{64}Cu]Cu-4**	**[^{64}Cu]Cu-8a**
**Percent fast [%]**	86.95 ±2.10	85.43 ±1.50	85.28 ±1.62	84.87 ±1.35
**Blood half life fast [min]**	1.21 ±0.21	1.24 ±0.15	1.30 ±0.17	1.27 ±0.13
**Blood half life slow [min]**	32.52 ±3.08	38.24 ±2.68	32.05 ±2.09	35.91 ±2.08

Table S5: Blood kinetic parameters for the ^{68}Ga-labeled ligands

Parameters were derived from non-linear regression of the TAC for heart uptake according to the model of two-phase decay.

	[^{68}Ga]Ga-1e	[^{68}Ga]Ga-4	[^{68}Ga]Ga-8c	[^{68}Ga]Ga-8d
**Percent fast [%]**	80.73 ±3.35	78.47 ±3.76	78.28 ±1.97	83.87 ±1.35
**Blood half life fast [min]**	1.51 ±0.31	1.36 ±0.41	1.44 ±0.19	1.50 ±0.15
**Blood half life slow [min]**	42.24 ±4.20	39.14 ±6.67	38.77 ±2.49	37.12 ±2.19

Compound characterization data

Bicyclic peptides

1a

1a (2.0 mg, 4 %) was obtained as a purple solid.

HPLC chromatogram

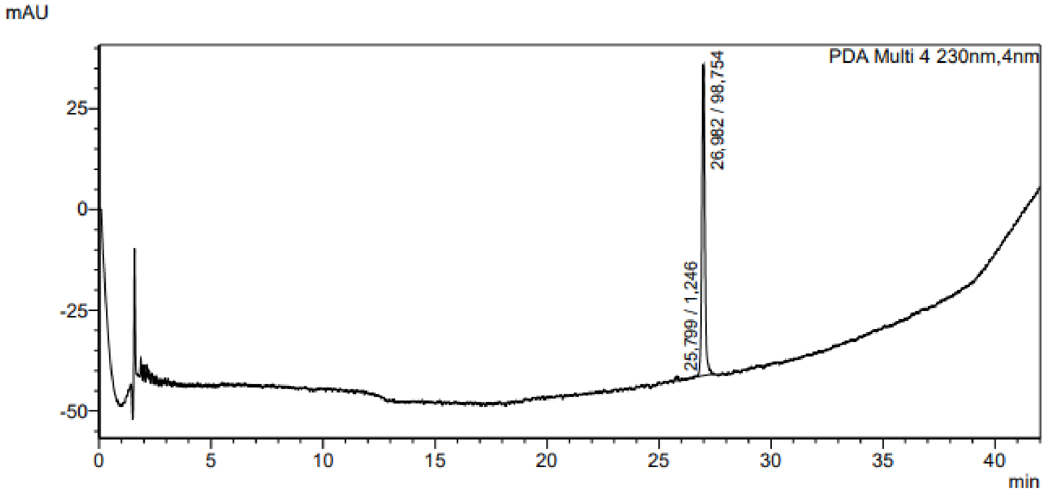

Peak#	Ret. Time	Area	Area%
1	25,799	8741	1,246
2	26,982	693005	98,754
Total		701746	100,000

HRMS spectrum (ESI+)

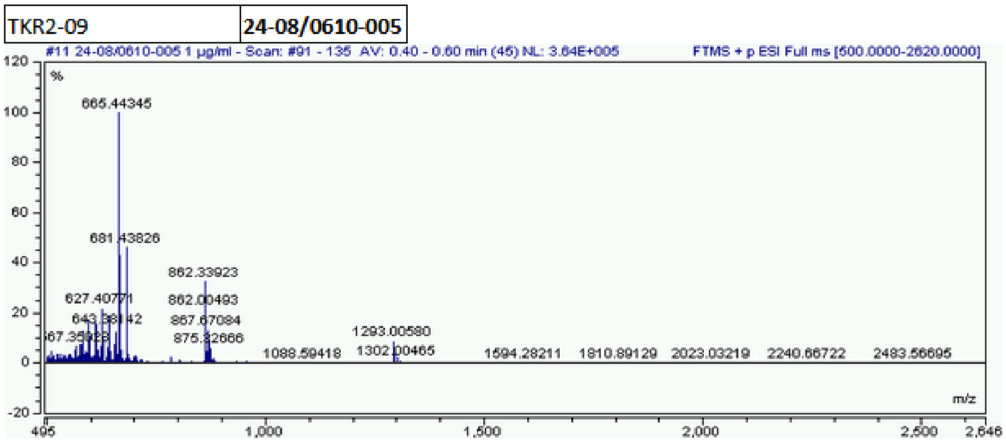

1b

1b (4.2 mg, 12 %) was obtained as a yellow solid.

HPLC chromatogram

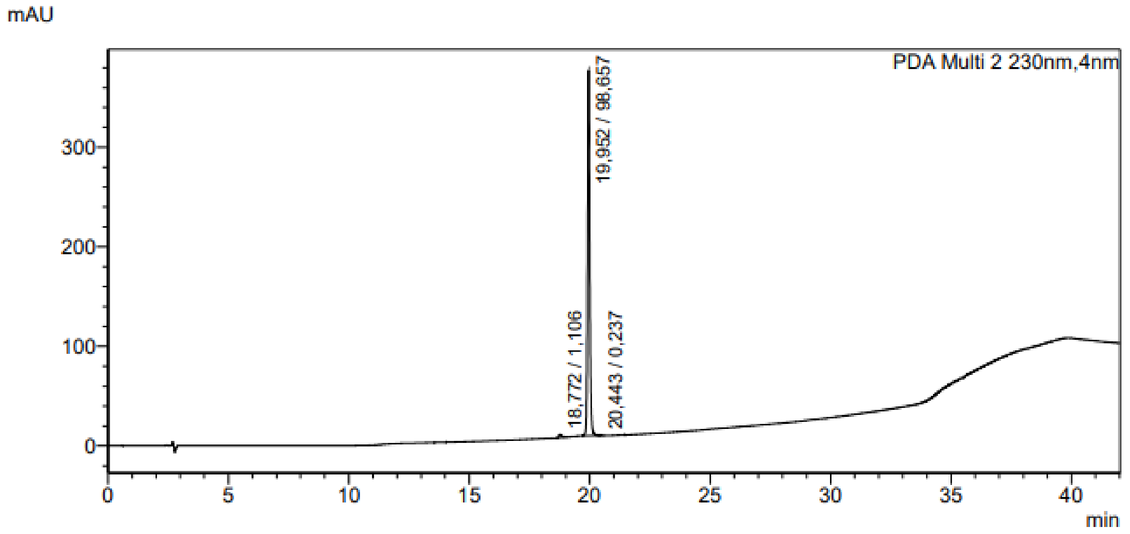

PDA Ch2 230nm			
Peak#	Ret. Time	Area	Area%
1	18,772	26234	1,106
2	19,952	2339816	98,657
3	20,443	5615	0,237
Total		2371665	100,000

HRMS spectrum (ESI+)

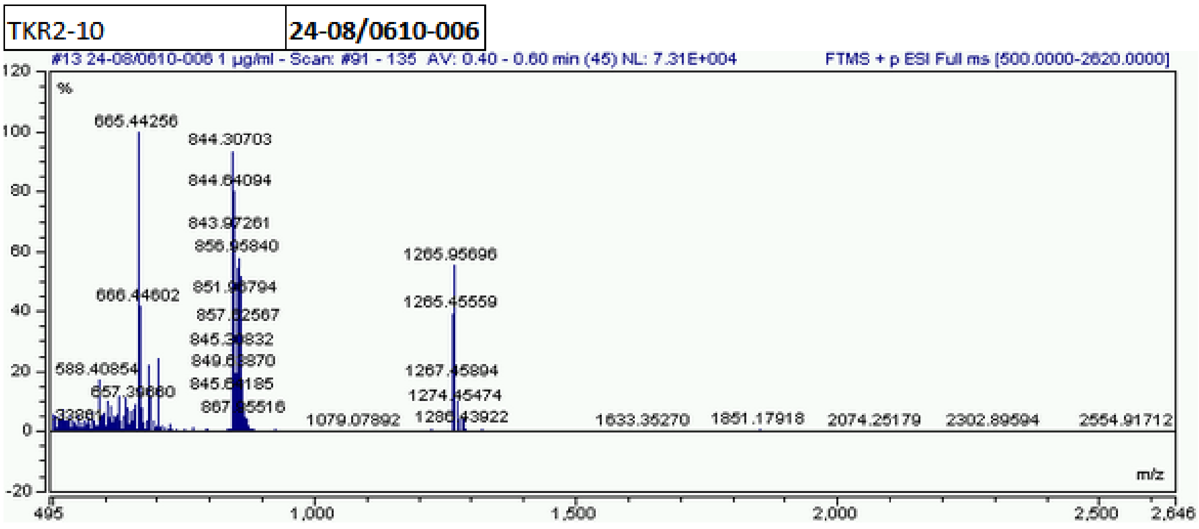

1c

1c (2.0 mg, 4 %) was obtained as a white solid.

HPLC chromatogram

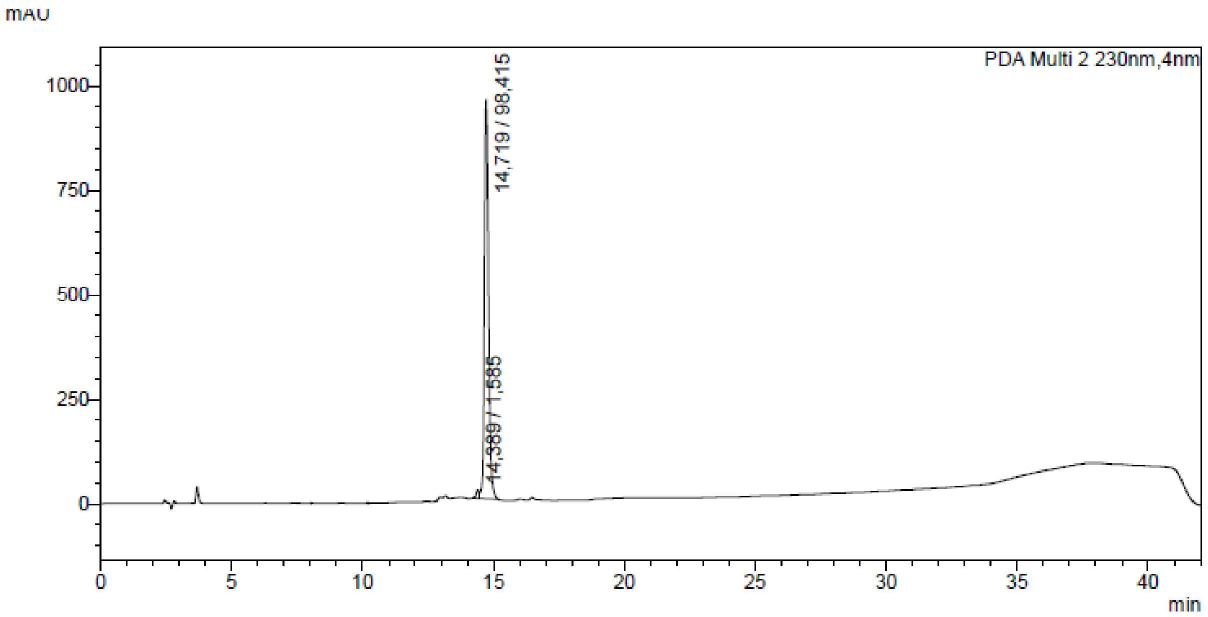

PDA Ch2 230nm

Peak#	Ret. Time	Area	Height	Conc.	Unit	Area%
1	14,389	170256	20719	1,585		1,585
2	14,719	10573863	955125	98,415		98,415
Total		10744119	975844			100,000

HRMS spectrum (ESI+)

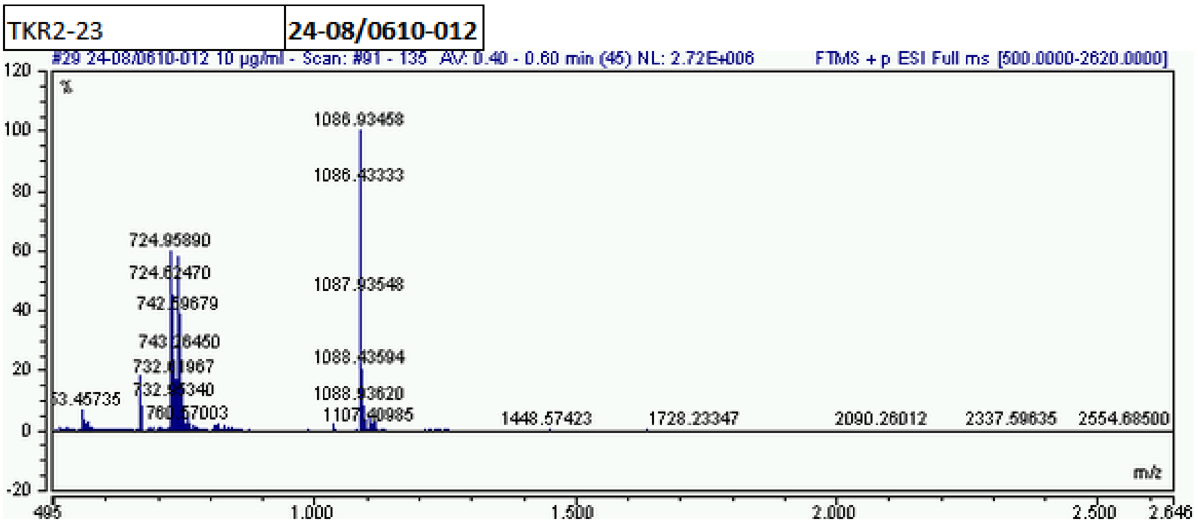

1d

1d (7.1 mg, 10%) was obtained as a white solid.

HPLC chromatogram

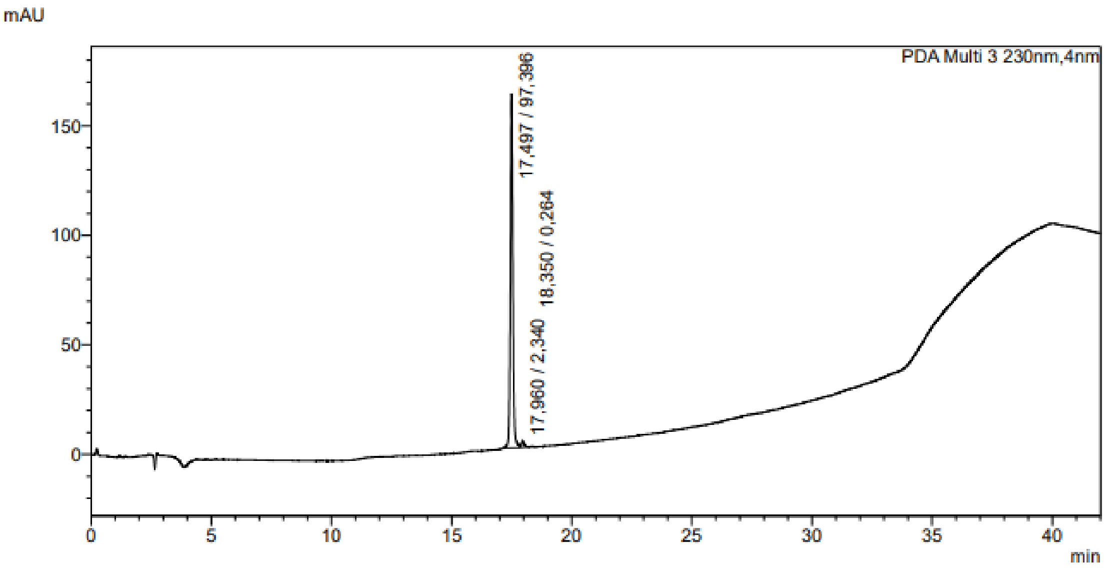

PDA Ch3 230nm						
Peak#	Ret. Time	Area	Height	Conc.	Unit	Area%
1	17,497	1152161	161079	0,000		97,396
2	17,960	27678	3145	0,000		2,340
3	18,350	3126	391	0,000		0,264
Total		1182965	164615			100,000

HRMS spectrum (ESI+)

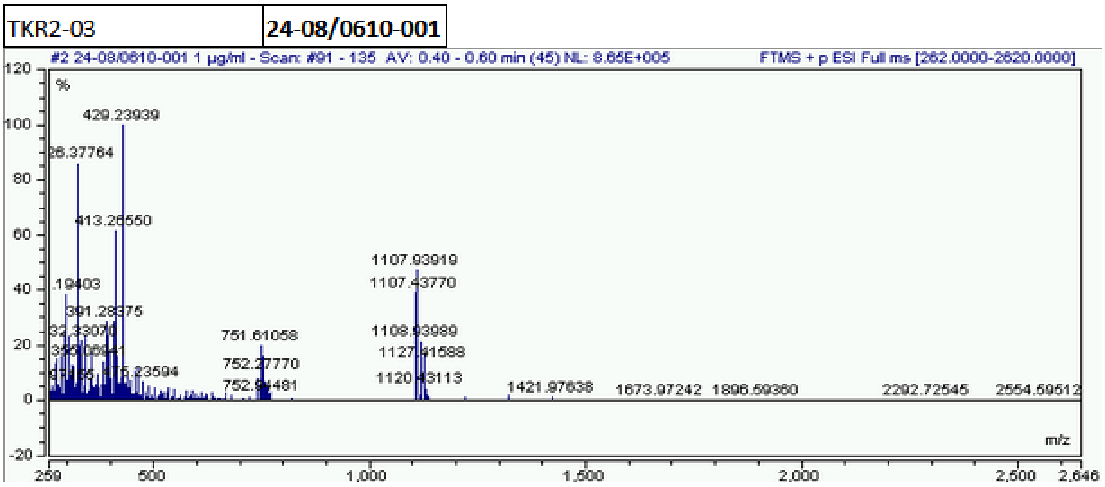

1e

1e (5.0 mg, 12%) was obtained as a white solid.

HPLC chromatogram (DMSO Stock)

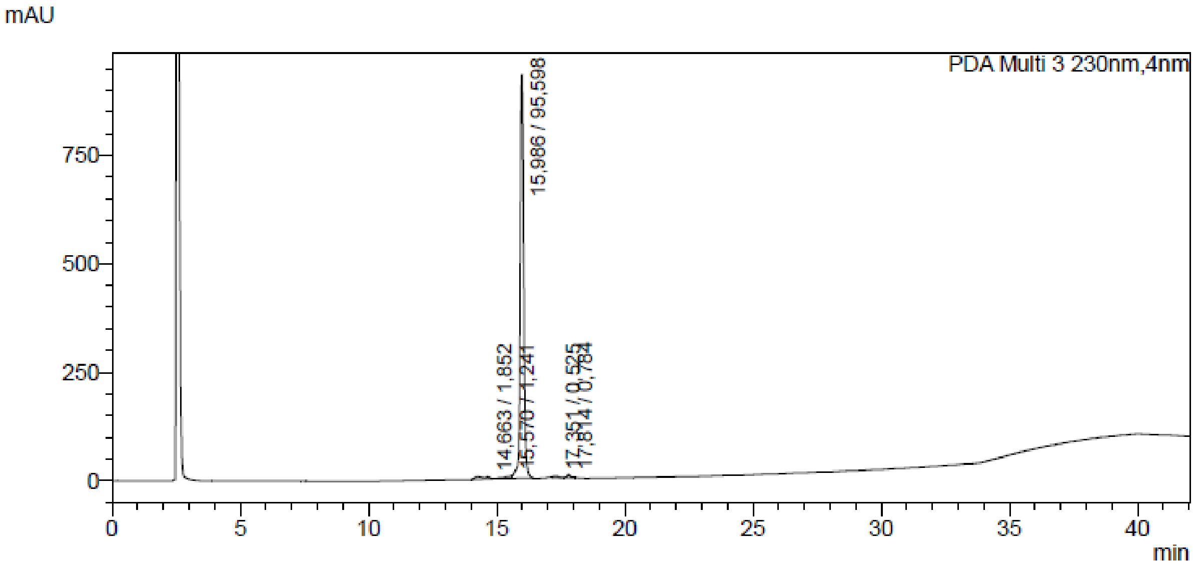

PDA Ch3 230nm

Peak#	Ret. Time	Area	Height	Area%
1	14,663	168444	5234	1,852
2	15,570	112853	5923	1,241
3	15,986	8694087	927558	95,598
4	17,351	47756	3630	0,525
5	17,814	71308	7625	0,784
Total		9094448	949969	100,000

HRMS spectrum (ESI+)

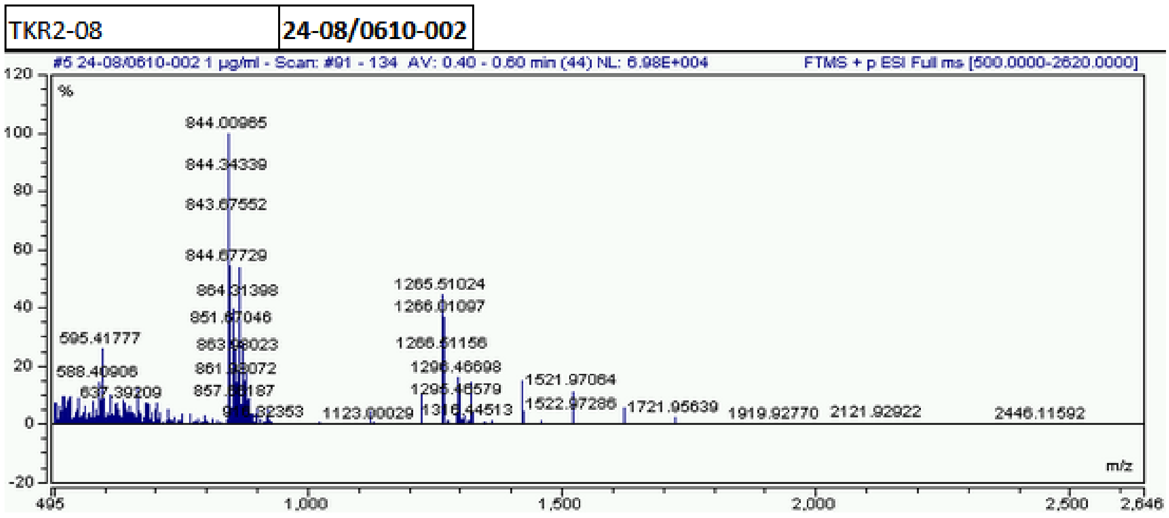

^{nat}Cu-1e

^{nat}Cu-1e (0.7 mg, 80%) was obtained as a bluish solid.

HPLC chromatogram

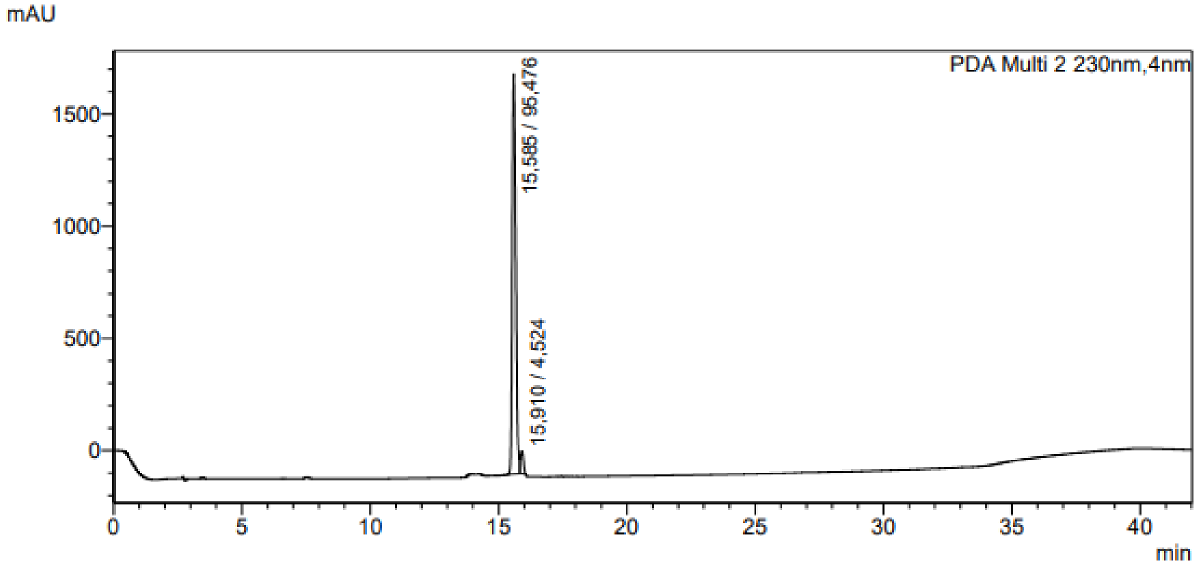

PDA Ch2 230nm

Peak#	Ret. Time	Area	Area%
1	15,585	17427005	95,476
2	15,910	825696	4,524
Total		18252700	100,000

HRMS spectrum (ESI+)

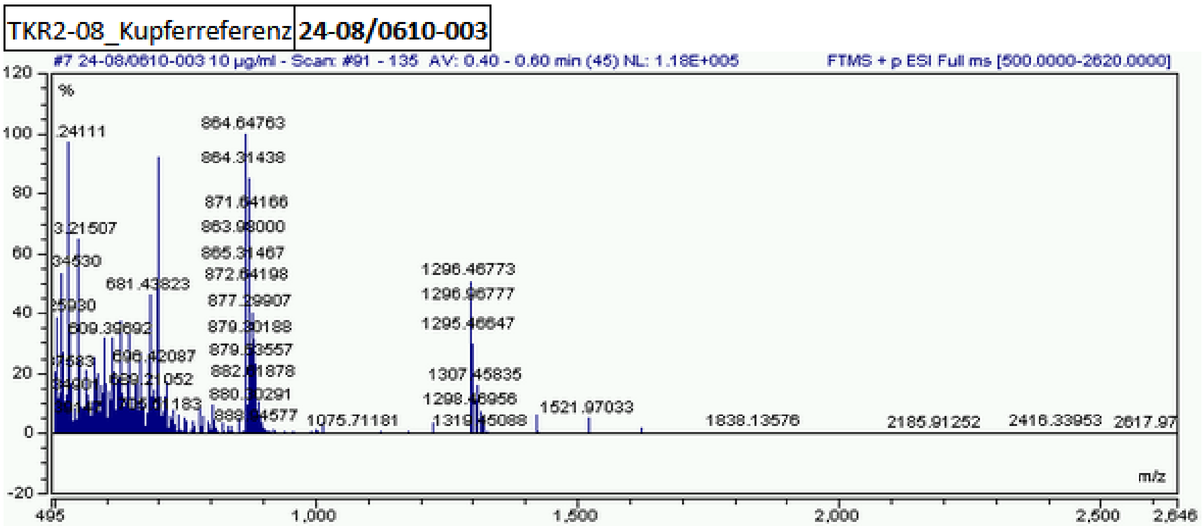

^{nat}Ga-1e

^{nat}Ga-1e (0.7 mg, 80%) was obtained as a white solid.

HPLC chromatogram (DMSO Stock)

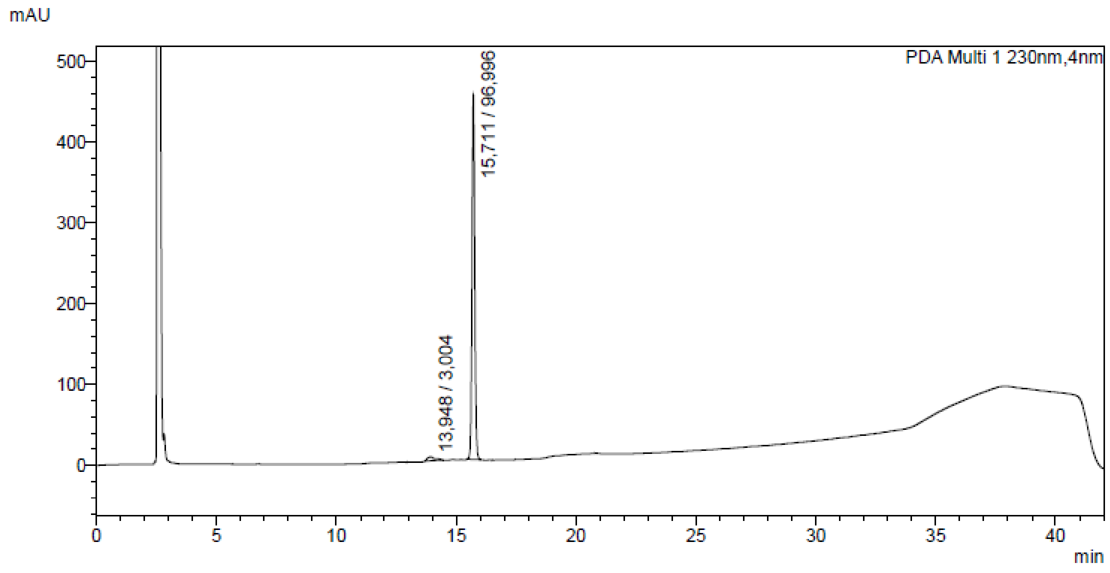

PDA Ch1 230nm						
Peak#	Ret. Time	Area	Height	Conc.	Unit	Area%
1	13,948	110222	4820	0,000		3,004
2	15,711	3558847	451564	0,000		96,996
Total		3669069	456384			100,000

HRMS spectrum (ESI+)

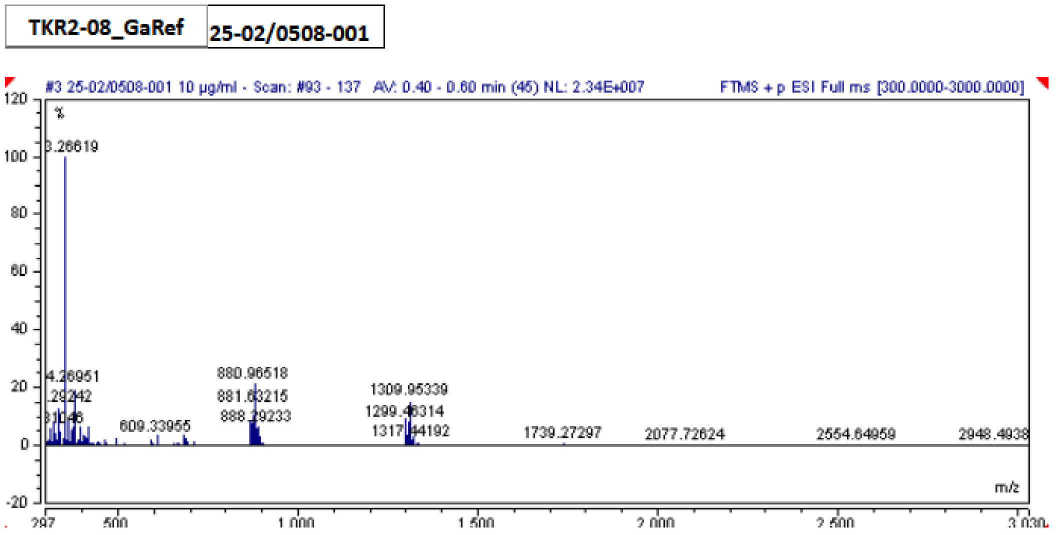

1f

1f (5.8 mg, 12 %) was obtained as a white solid.

HPLC chromatogram (DMSO Stock)

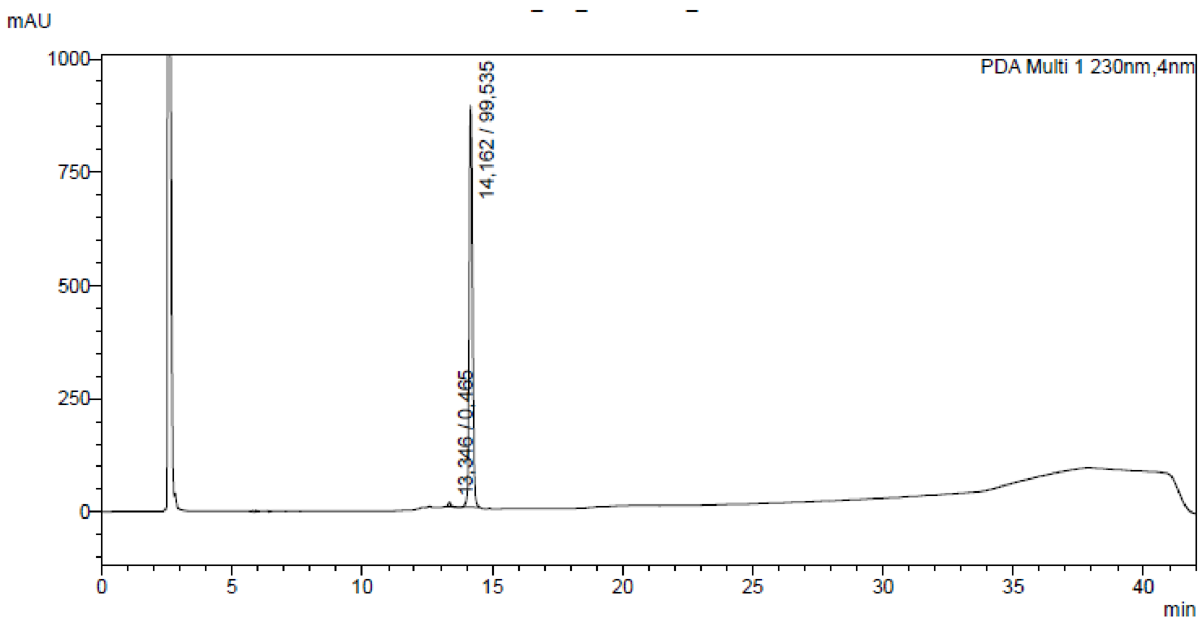

PDA Ch1 230nm

Peak#	Ret. Time	Area	Height	Conc.	Unit	Area%
1	13,346	36974	9397	0,000		0,465
2	14,162	7921881	881750	0,000		99,535
Total		7958855	891147			100,000

HRMS spectrum (ESI+)

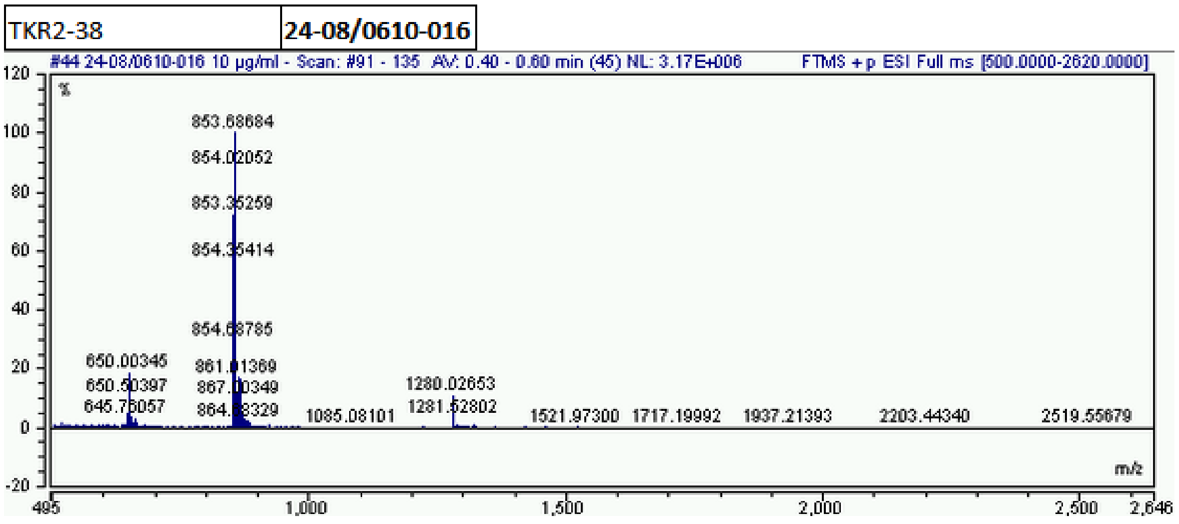

2

2 (0.5 mg, 60%) was obtained as a white solid.

HPLC chromatogram (DMSO Stock)

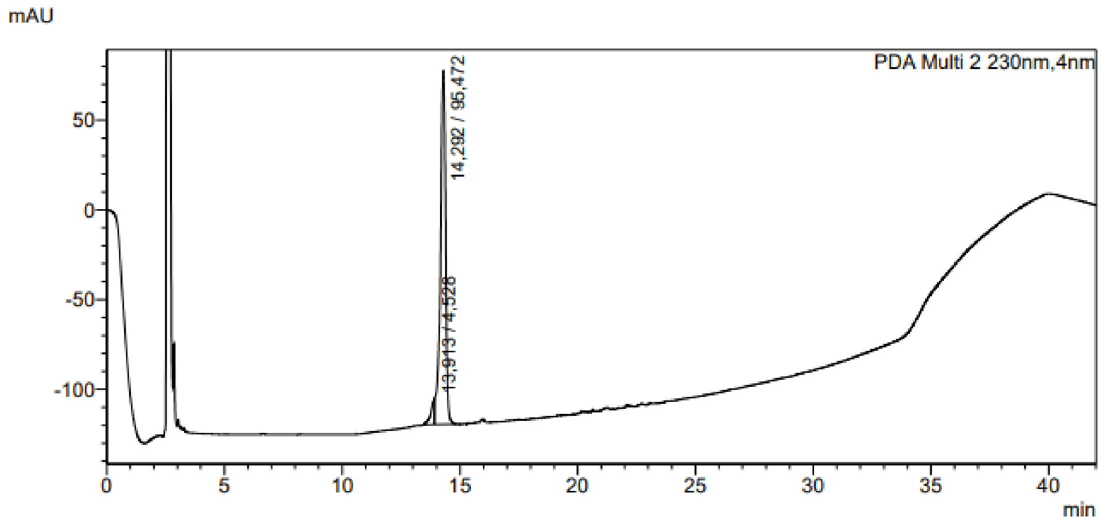

PDA Ch2 230nm

Peak#	Ret. Time	Area%
1	13,913	4,528
2	14,292	95,472
Total		100,000

HRMS spectrum (ESI+)

TKR2-08_Oxidiert	24-08/0610-004

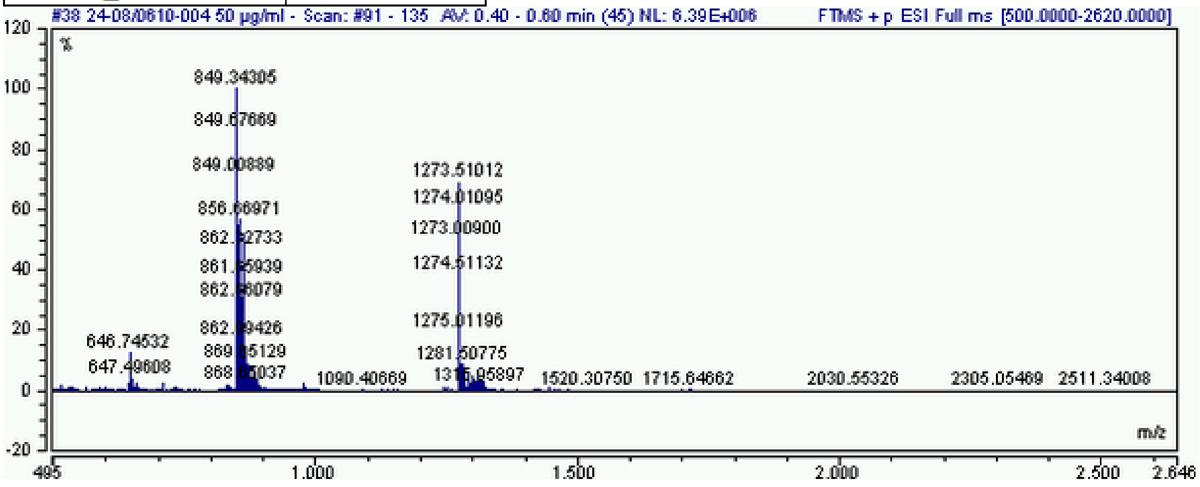

3a

3a (6.0 mg, 13 %) was obtained as a white solid.

HPLC chromatogram (DMSO Stock)

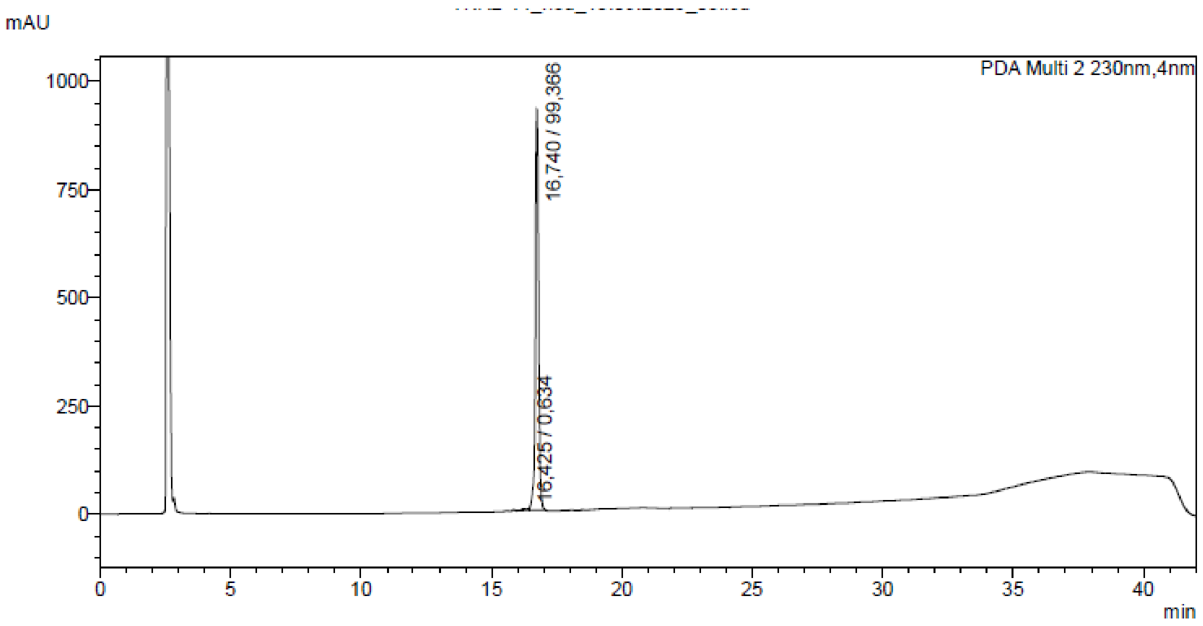

PDA Ch2 230nm Peak#	Ret. Time	Area	Height	Conc.	Unit	Area%
1	16,425	50972	3524	0,634		0,634
2	16,740	7987499	927095	99,366		99,366
Total		8038471	930619			100,000

HRMS spectrum (ESI+)

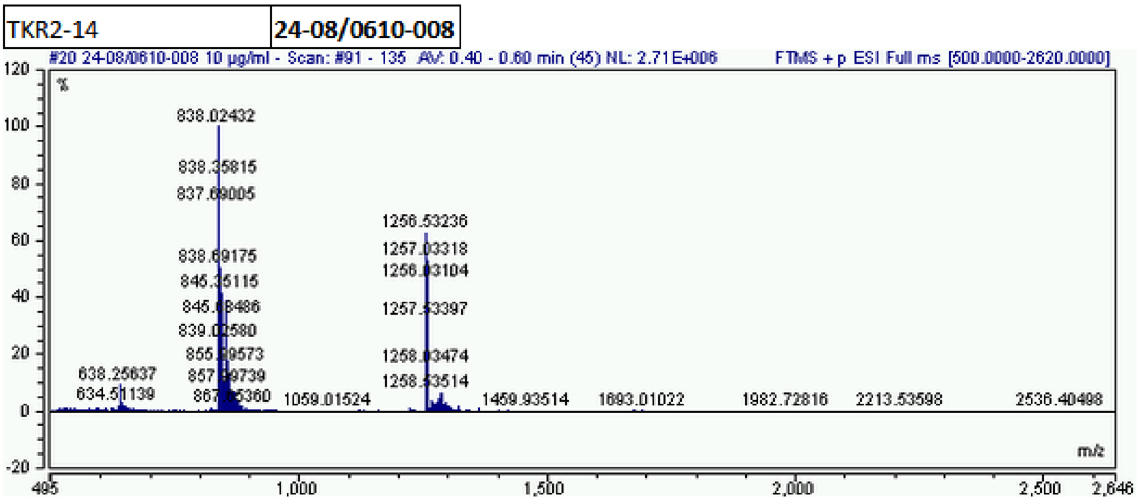

^{nat}Cu-3a

^{nat}Cu-3a (0.7 mg, 80 %) was obtained as a white solid.

HPLC chromatogram

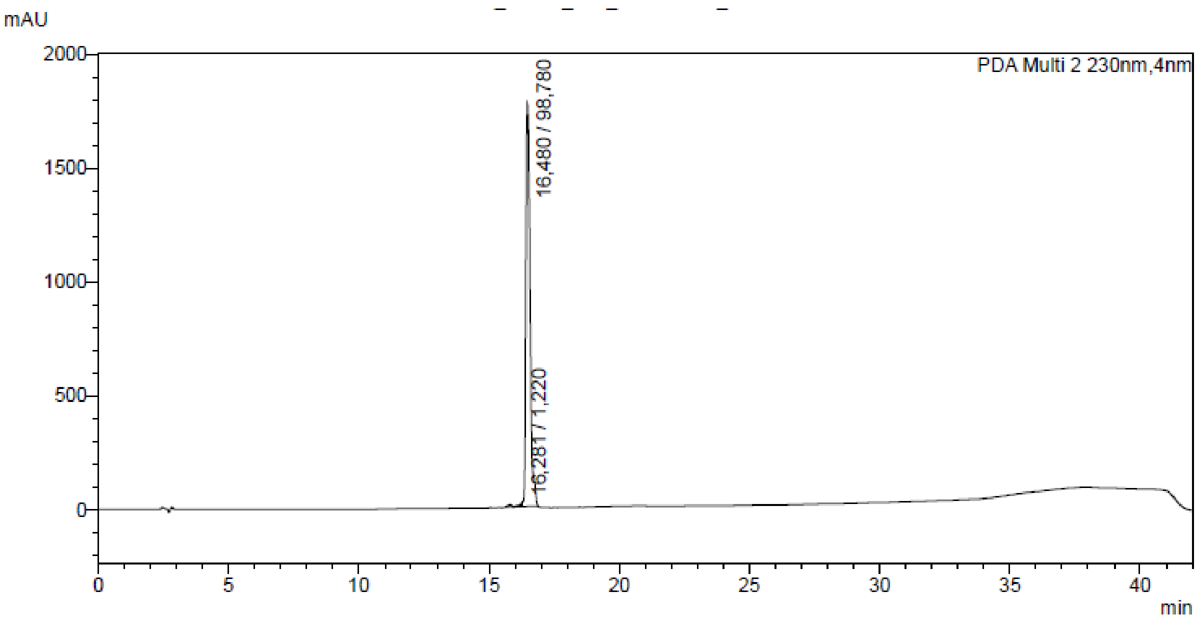

PDA Ch2 230nm						
Peak#	Ret. Time	Area	Height	Conc.	Unit	Area%
1	16,281	240684	18614	1,220		1,220
2	16,480	19480460	1761959	98,780		98,780
Total		19721144	1780573			100,000

HRMS spectrum (ESI+)

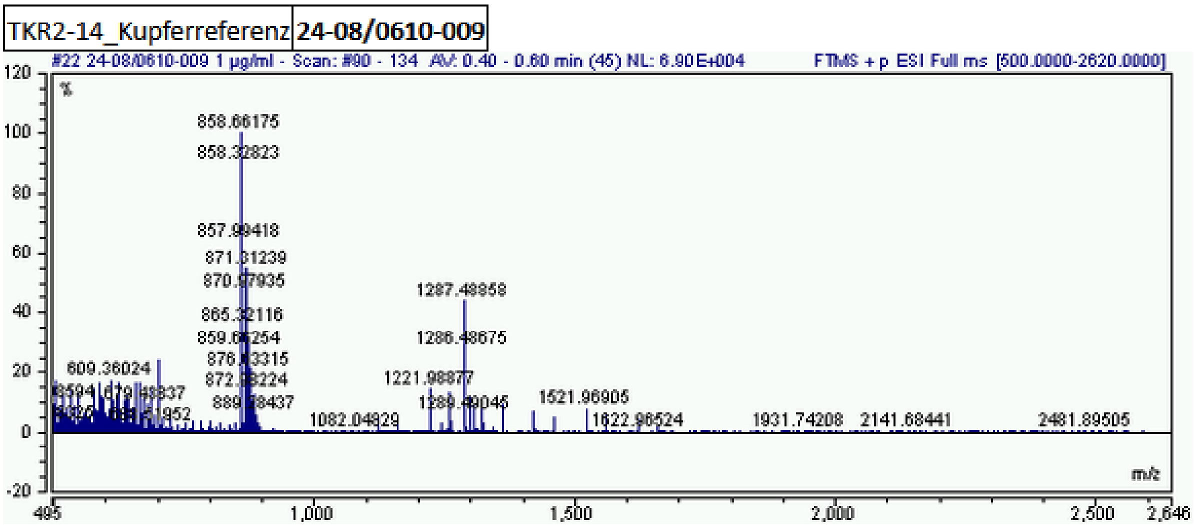

3b

3b (9.8 mg, 10 %) was obtained as a white solid.

HPLC chromatogram

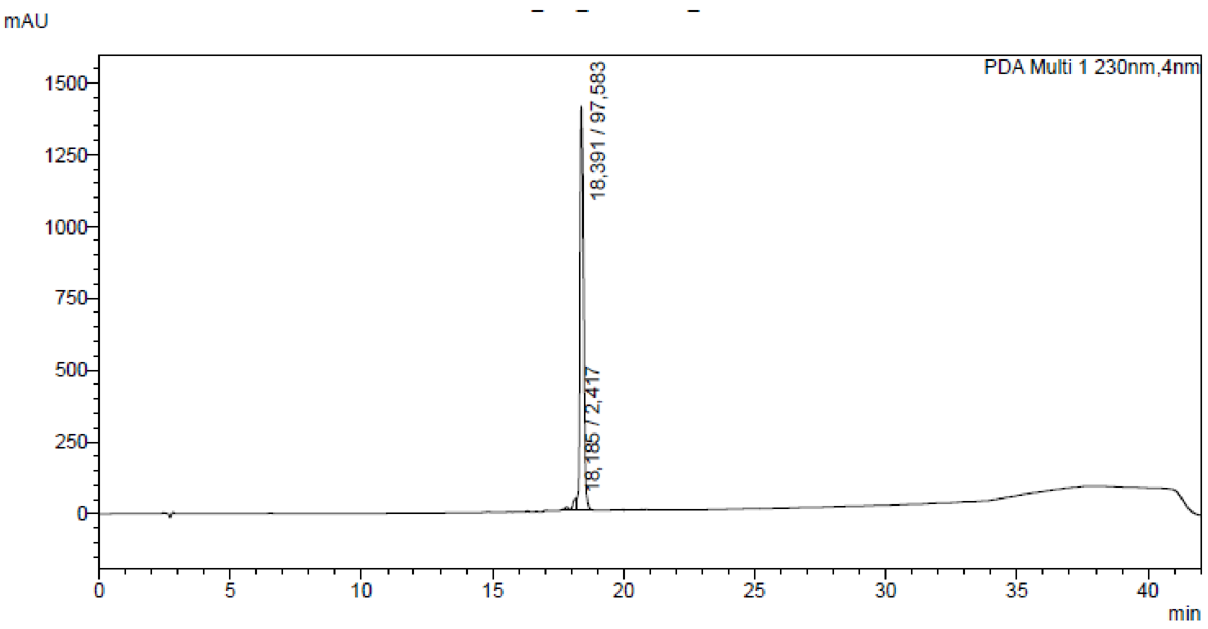

PDA Ch1 230nm

Peak#	Ret. Time	Area	Height	Conc.	Unit	Area%
1	18,185	335834	36575	0,000		2,417
2	18,391	13559055	1397118	0,000		97,583
Total		13894889	1433693			100,000

HRMS spectrum (ESI+)

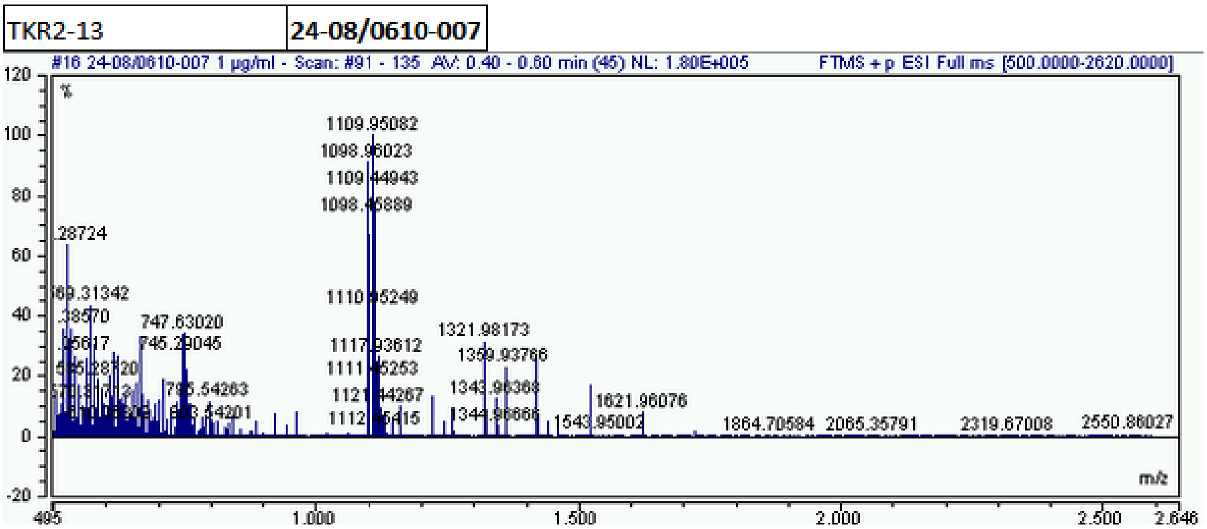

4

4 (6.4 mg, 9%) was obtained as a white solid.

HPLC chromatogram

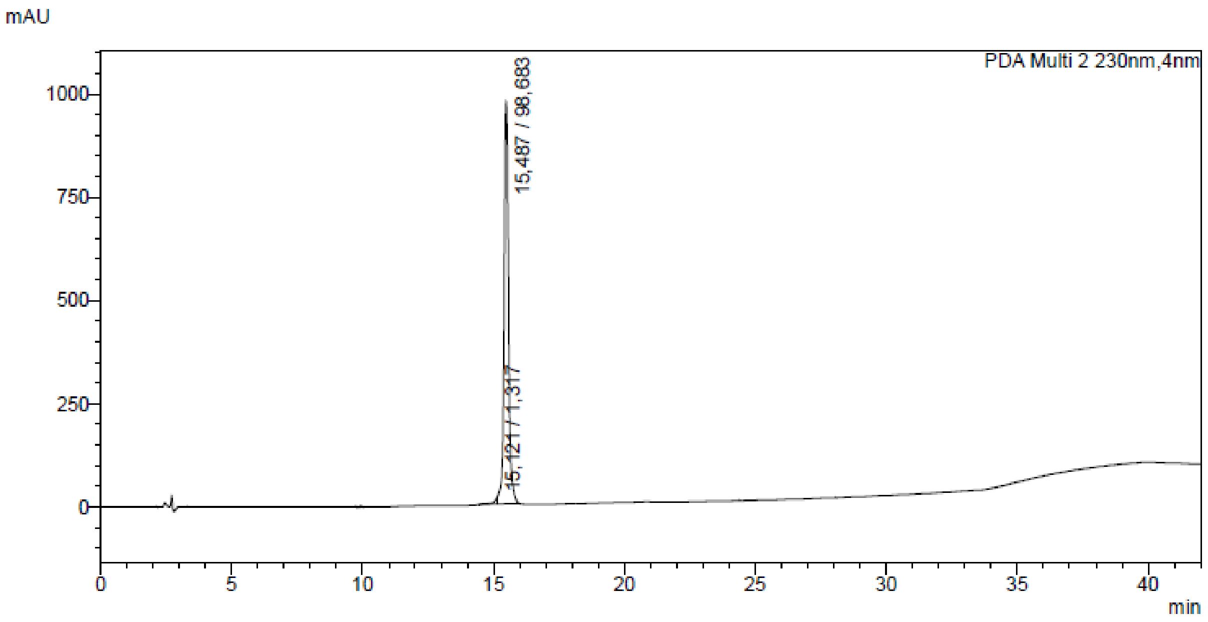

PDA Ch2 230nm

Peak#	Ret. Time	Area	Height	Conc.	Unit	Area%
1	15,121	154203	13961	0,000		1,317
2	15,487	11553777	974226	0,000		98,683
Total		11707980	988187			100,000

HRMS spectrum (ESI+)

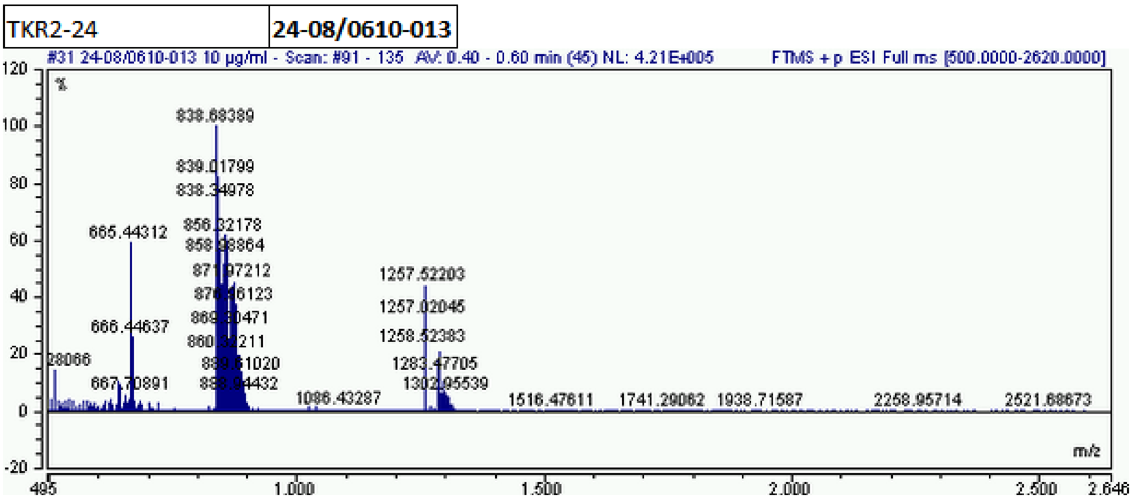

5

5 (2.3 mg, 6%) was obtained as a white solid.

HPLC chromatogram

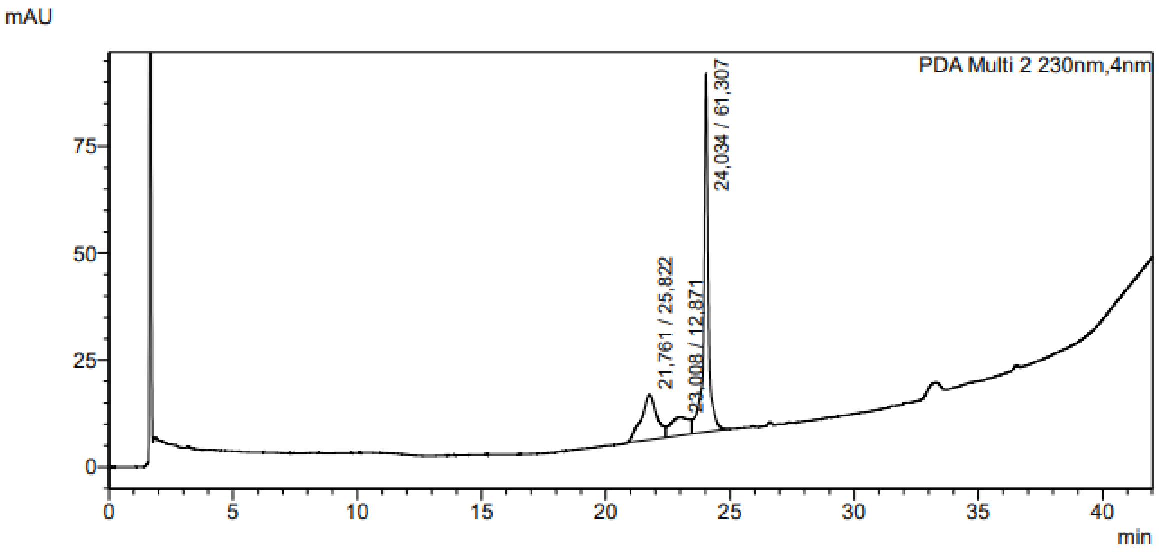

PDA Ch2 230nm

Peak#	Ret. Time	Area	Area%
1	21,761	458113	25,822
2	23,008	228336	12,871
3	24,034	1087648	61,307
Total		1774097	100,000

HRMS spectrum (ESI+)

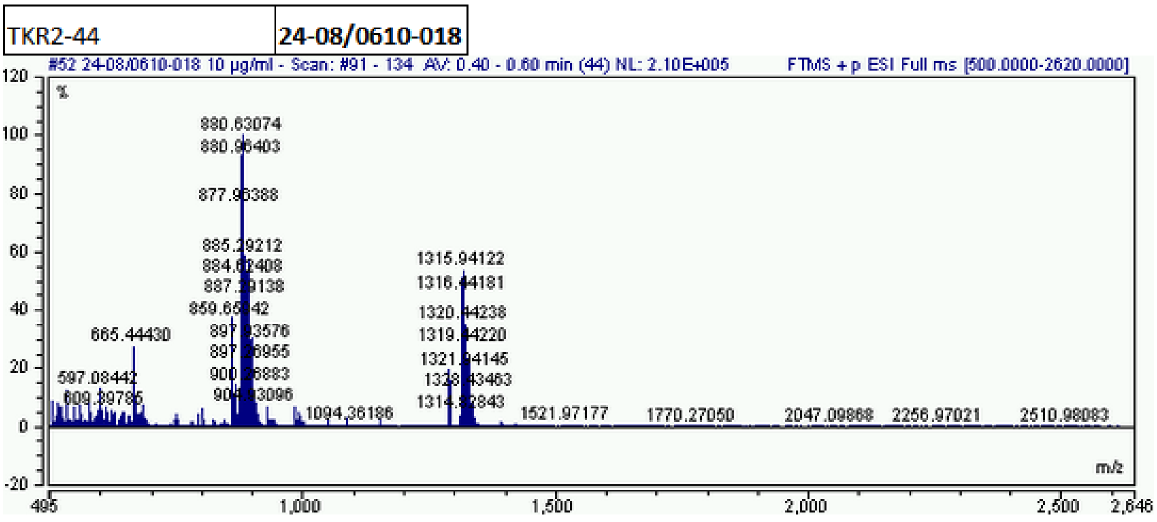

6

6 (4.7 mg, 7%) was obtained as a white solid.

HPLC chromatogram

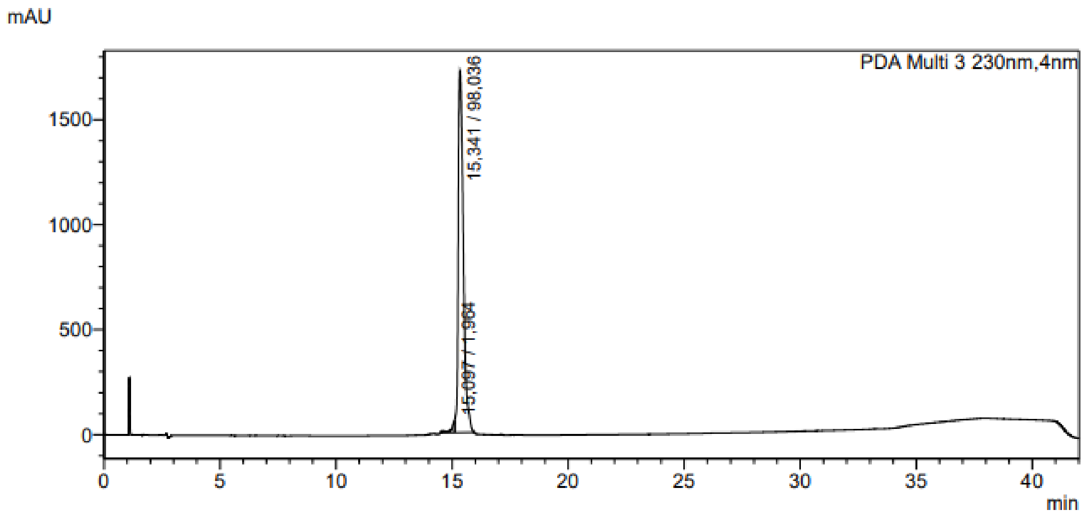

PDA Ch3 230nm

Peak#	Ret. Time	Area%
1	15,097	1,964
2	15,341	98,036
Total		100,000

HRMS spectrum (ESI+)

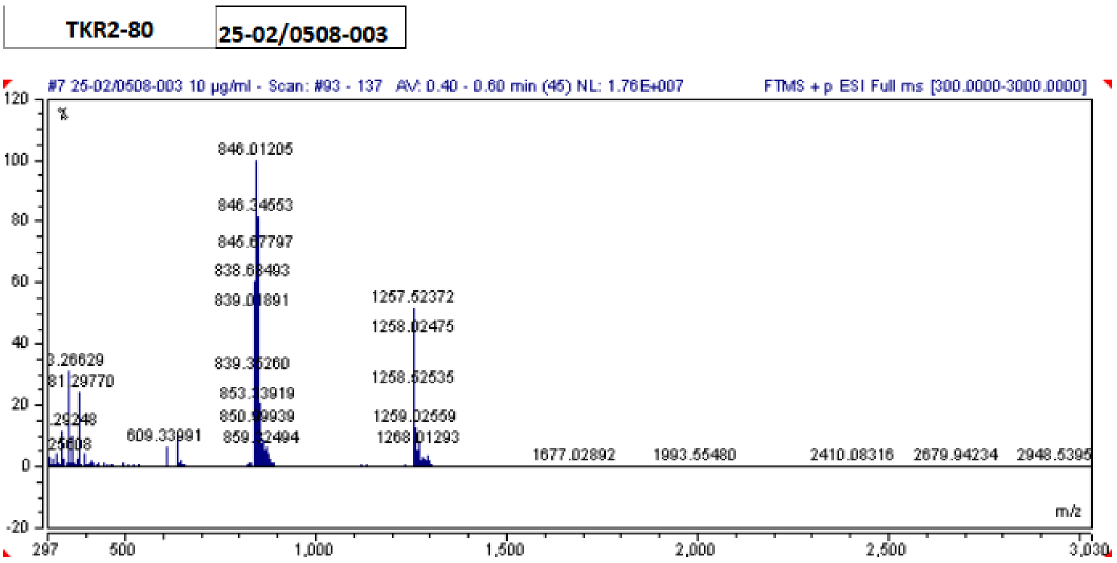

7

7 (5.2 mg, 8%) was obtained as a white solid.

HPLC chromatogram

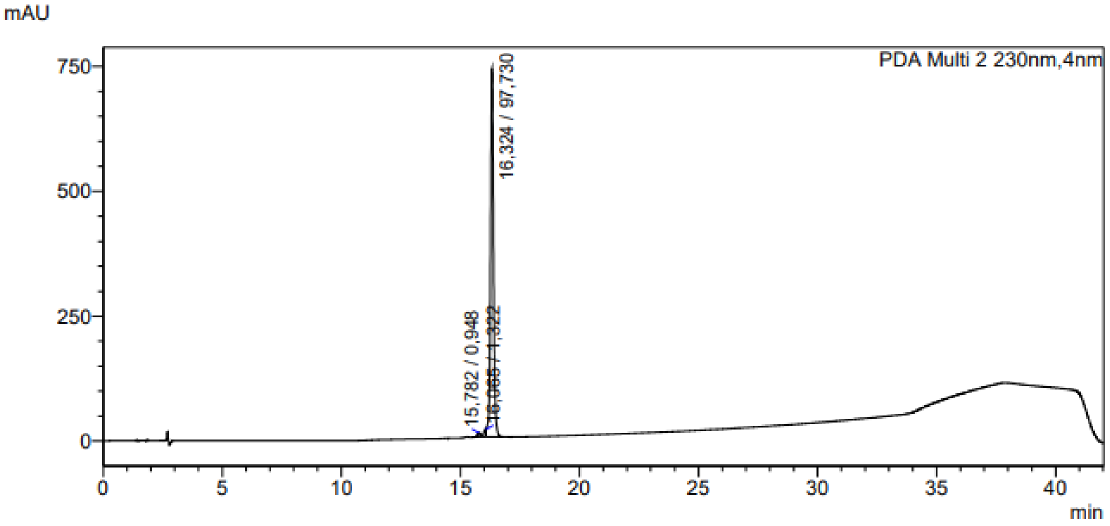

PDA Ch2 230nm

Peak#	Ret. Time	Area%
1	15,782	0,948
2	16,065	1,322
3	16,324	97,730
Total		100,000

HRMS spectrum (ESI+)

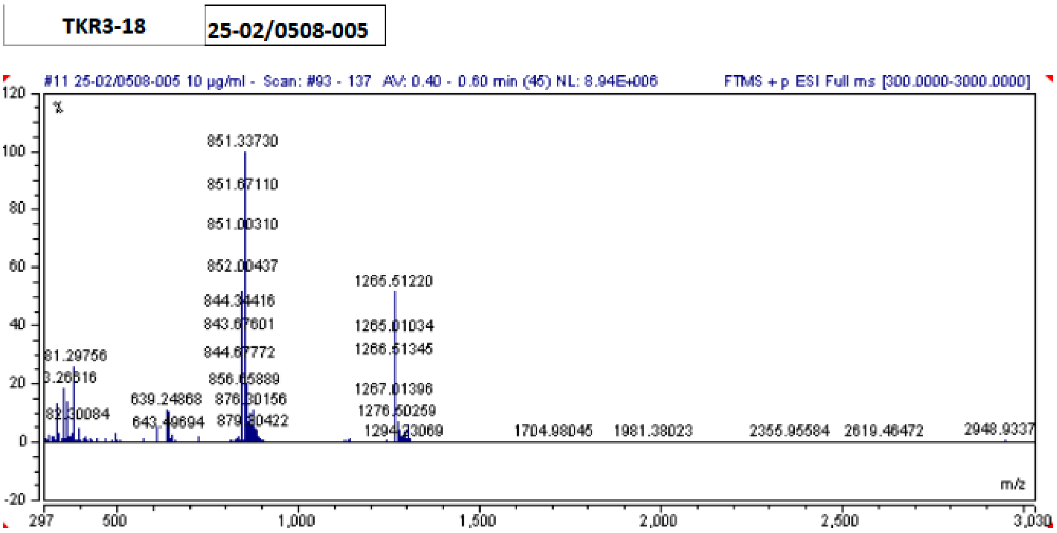

8a

8a (5.0 mg, 6%) was obtained as a white solid.

HPLC chromatogram

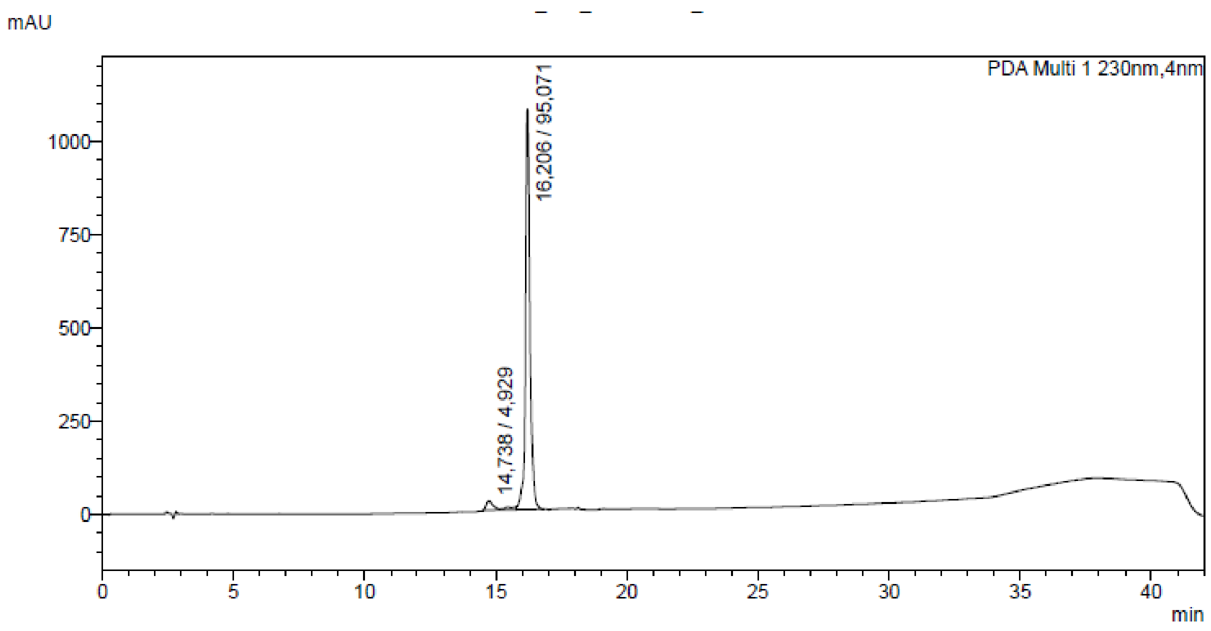

PDA Ch1 230nm

Peak#	Ret. Time	Area	Height	Conc.	Unit	Area%
1	14,738	649414	25675	0,000		4,929
2	16,206	12526166	1074146	0,000		95,071
Total		13175580	1099821			100,000

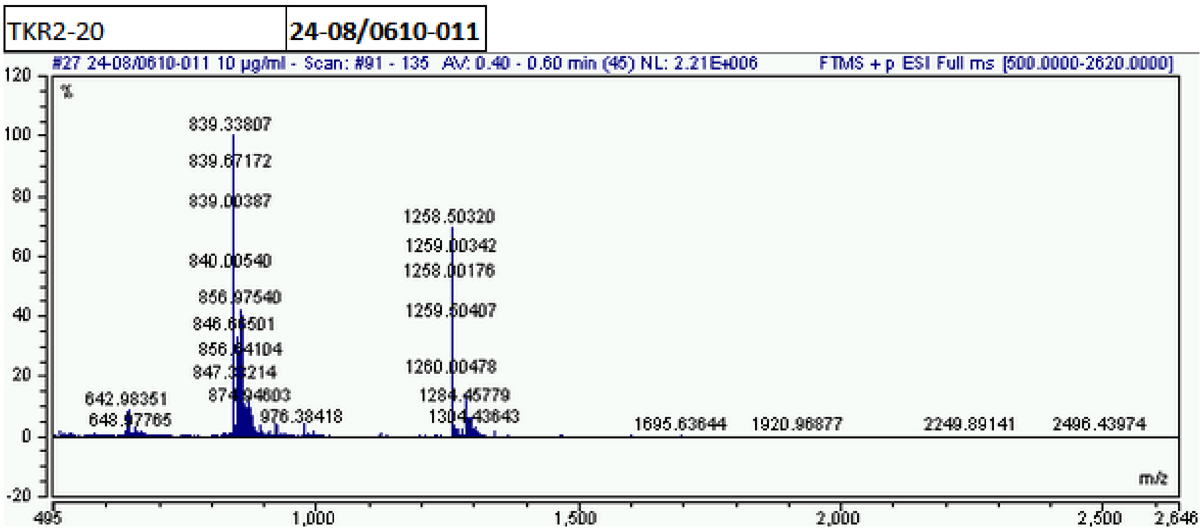

8b

8b (9.0 mg, 5%) was obtained as a white solid.

HPLC chromatogram

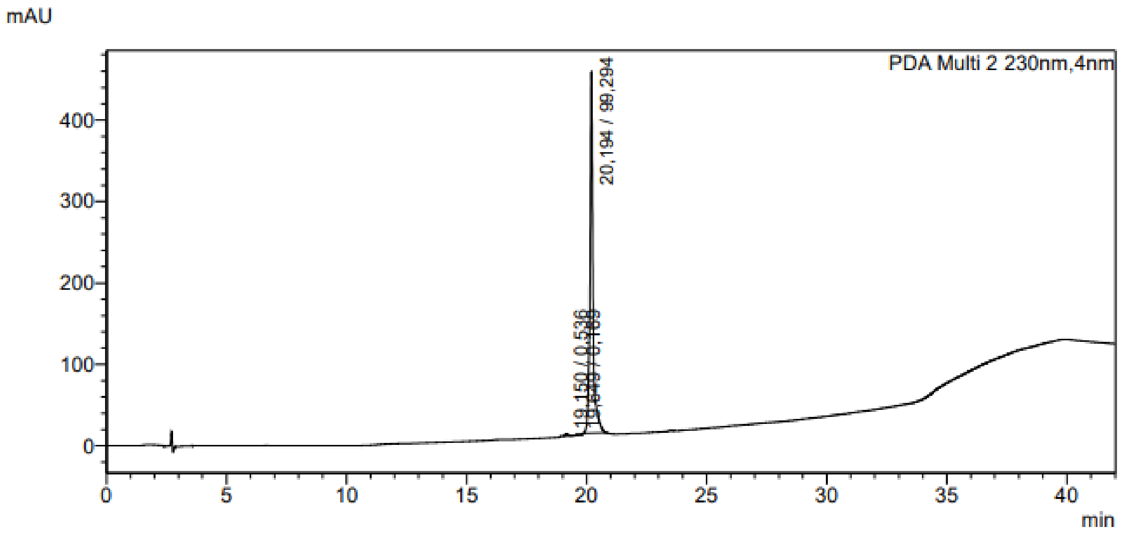

PDA Ch2 230nm

Peak#	Ret. Time	Area%
1	19,150	0,536
2	19,649	0,169
3	20,194	99,294
Total		100,000

HRMS spectrum (ESI+)

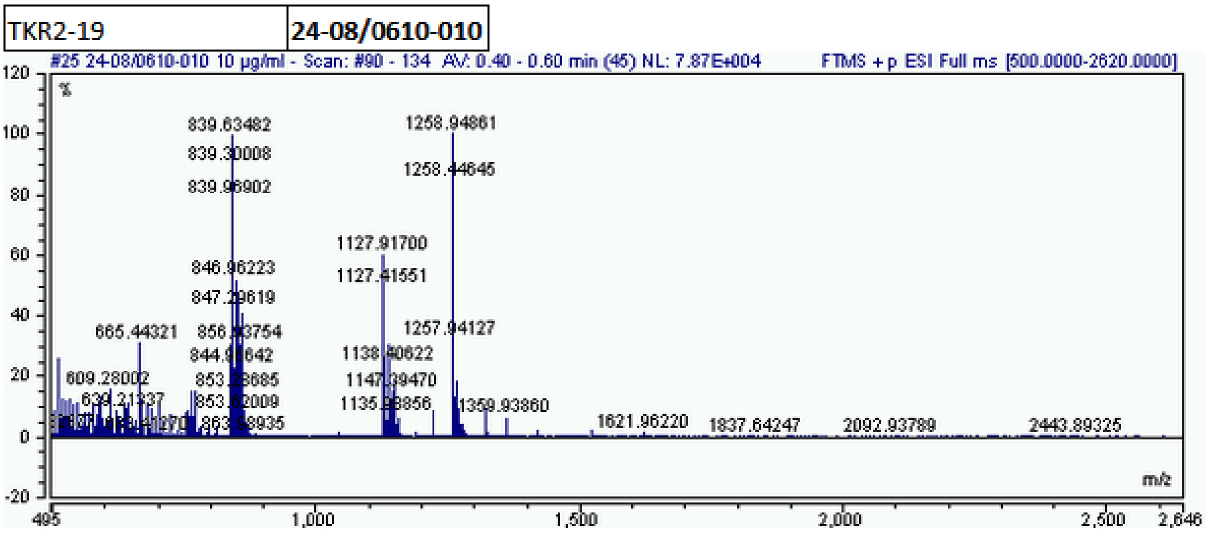

8c

8c (1.7 mg, 4%) was obtained as a white solid.

HPLC chromatogram

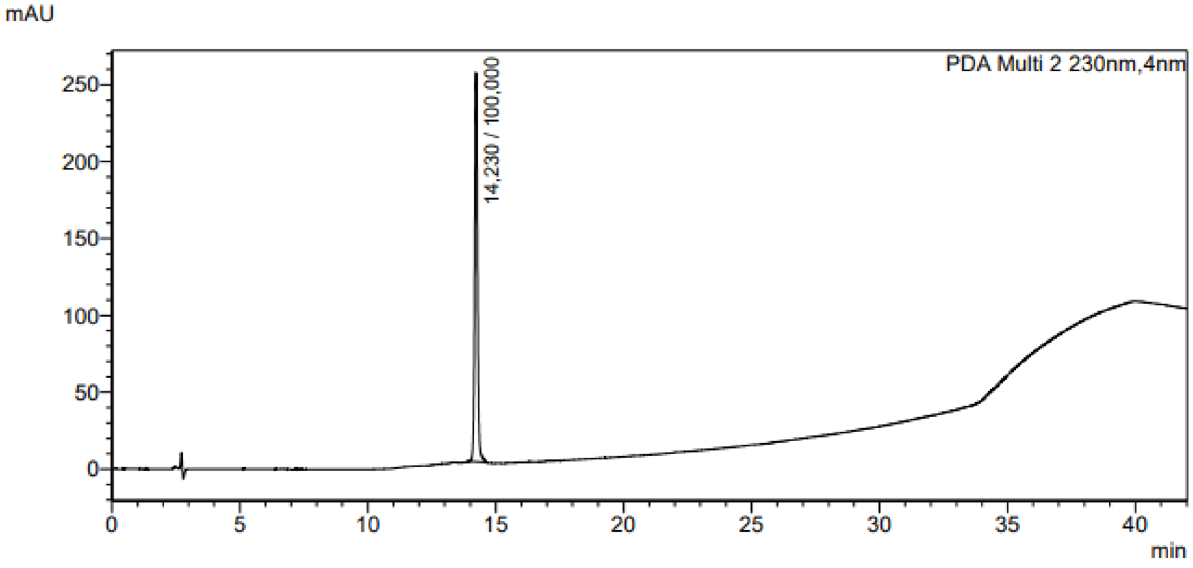

PDA Ch2 230nm

Peak#	Ret. Time	Area	Height	Area%
1	14,230	1925612	252547	100,000
Total		1925612	252547	100,000

HRMS spectrum (ESI+)

TKR2-52	24-08/0610-021

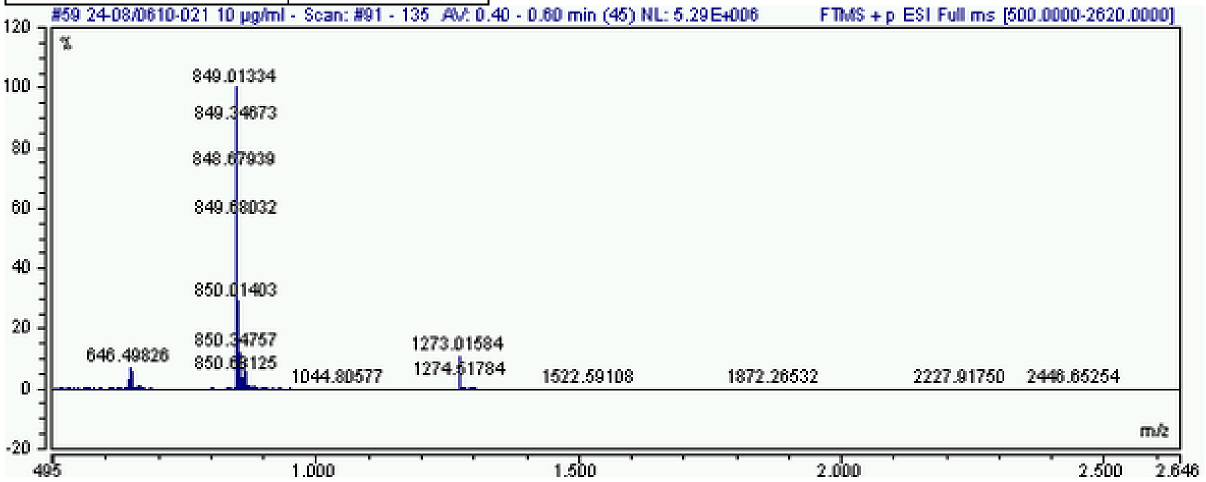

8d

8d (2.8 mg, 6 %) was obtained as a white solid.

HPLC chromatogram

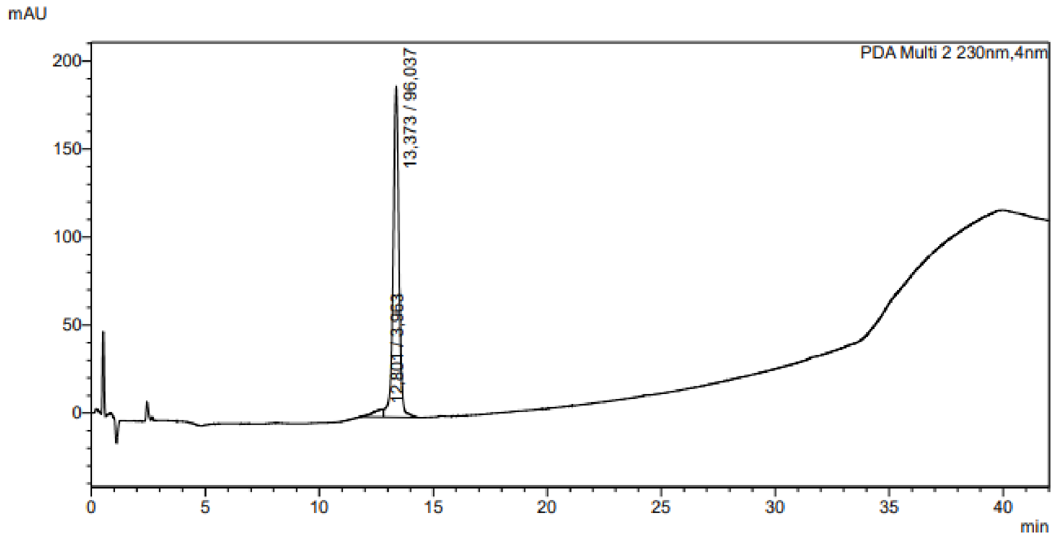

PDA Ch2 230nm

Peak#	Ret. Time	Area	Height	Conc.	Unit	Area%
1	12,801	136617	4480	0,000		3,963
2	13,373	3310473	187661	0,000		96,037
Total		3447090	192141			100,000

HRMS spectrum (ESI+)

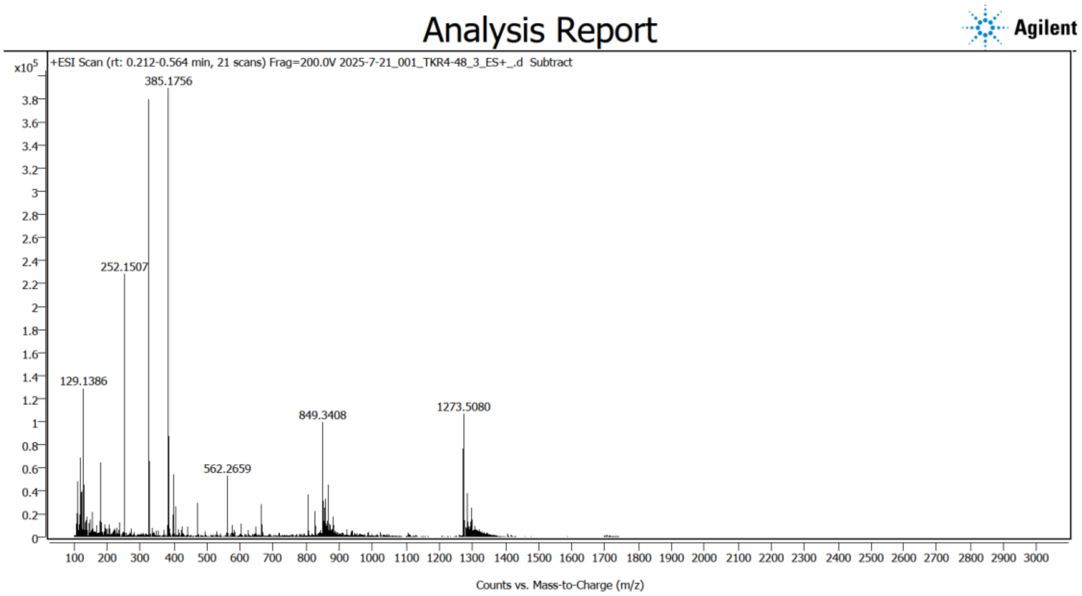

9

9 (3.1 mg, 8%) was obtained as a white solid.

HPLC chromatogram

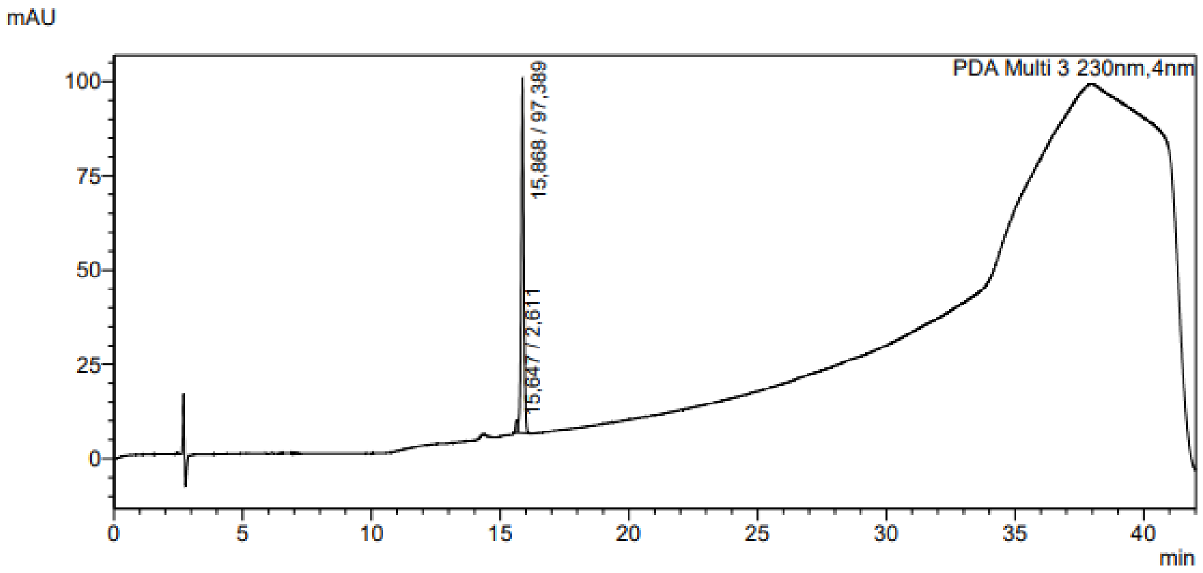

PDA Ch3 230nm

Peak#	Ret. Time	Area%
1	15,647	2,611
2	15,868	97,389
Total		100,000

HRMS spectrum (ESI+)

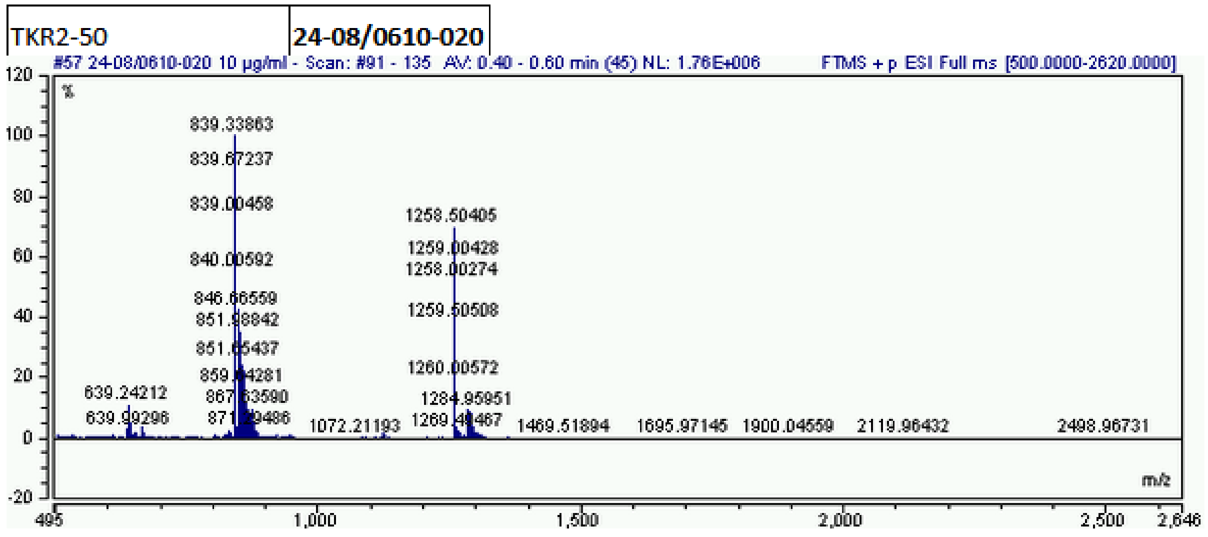

10

10 (5.8 mg, 8%) was obtained as a white solid.

HPLC chromatogram

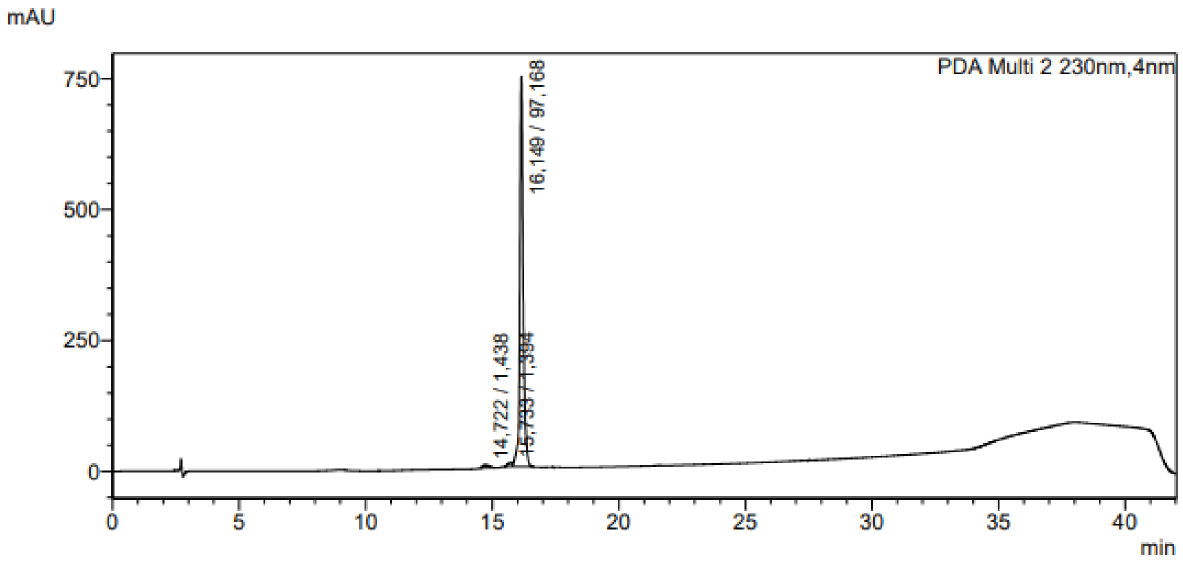

PDA Ch2 230nm

Peak#	Ret. Time	Area%
1	14,722	1,438
2	15,733	1,394
3	16,149	97,168
Total		100,000

HRMS spectrum (ESI+)

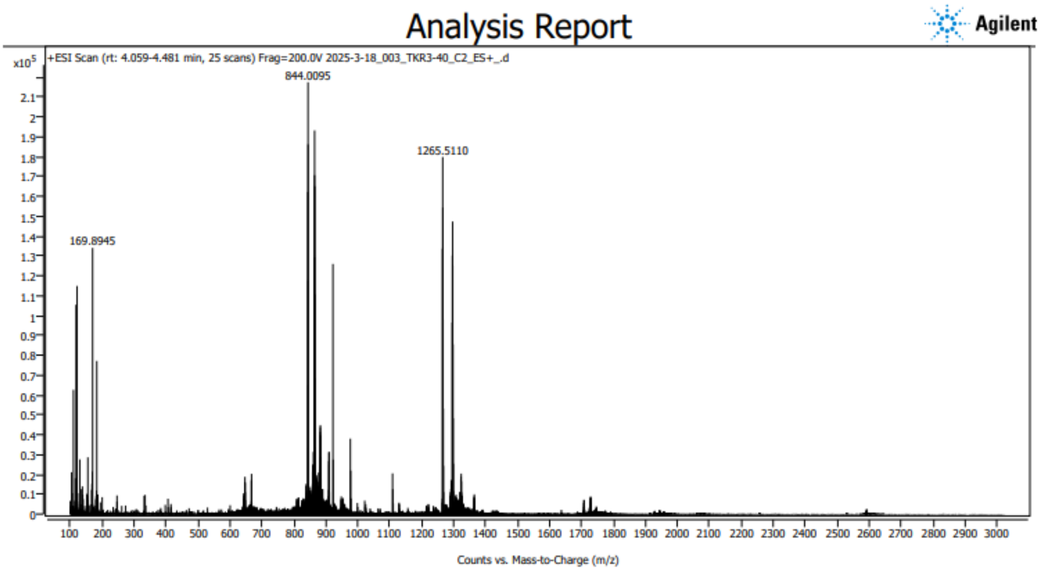

Radiolabeled bicyclic peptides

Exemplary radioactivity-detected chromatograms are shown below after labeling of the different peptides with copper-64 or gallium-68. Labeling yields and purity were usually ≥95% with the exception of compounds bearing methionine or selenomethionine for which radiochemical purity was ≤95% due to the formation of radiolabeled side products (see main article for discussion).

[^{64}Cu]Cu-1e

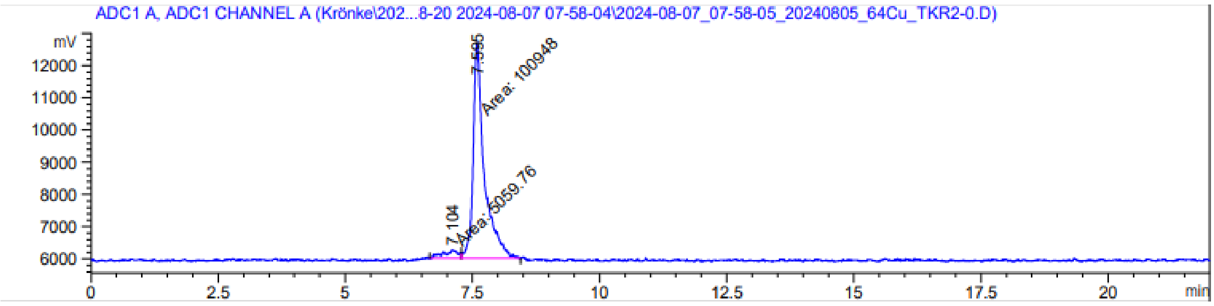

Peak #	RetTime [min]	Type	Width [min]	Area [mV*s]	Height [mV]	Area %
1	7.104	MF	0.3244	5059.75781	259.96082	4.7730
2	7.595	FM	0.2520	1.00948e5	6676.88818	95.2270

[^{68}Ga]Ga-1e

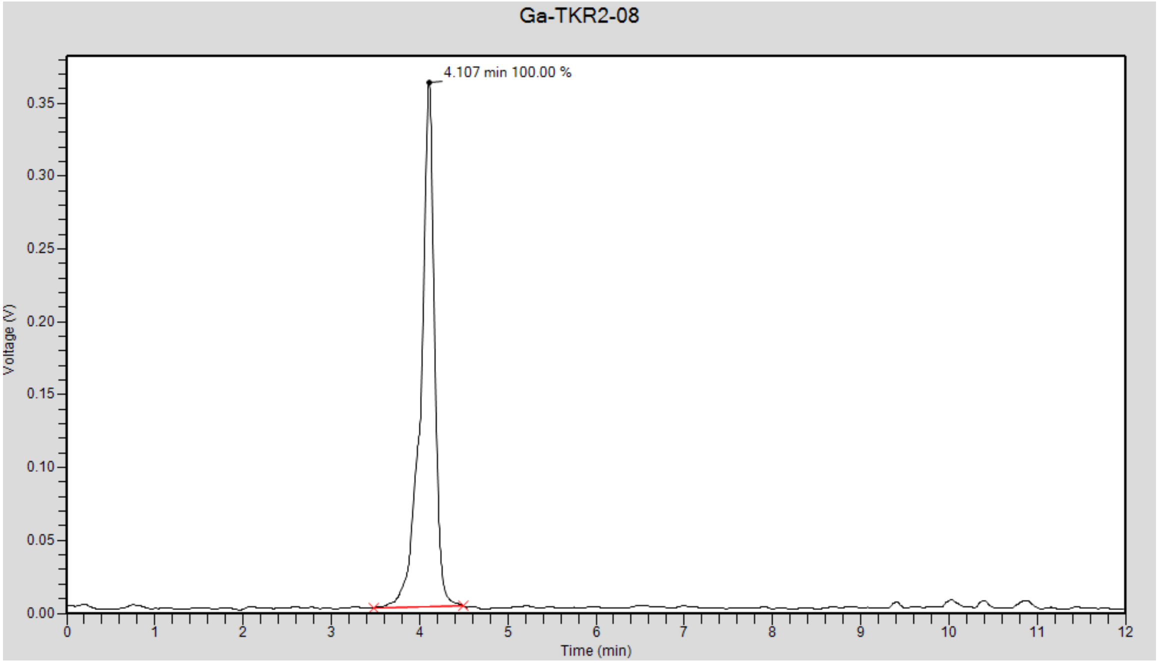

[^{64}Cu]Cu-2

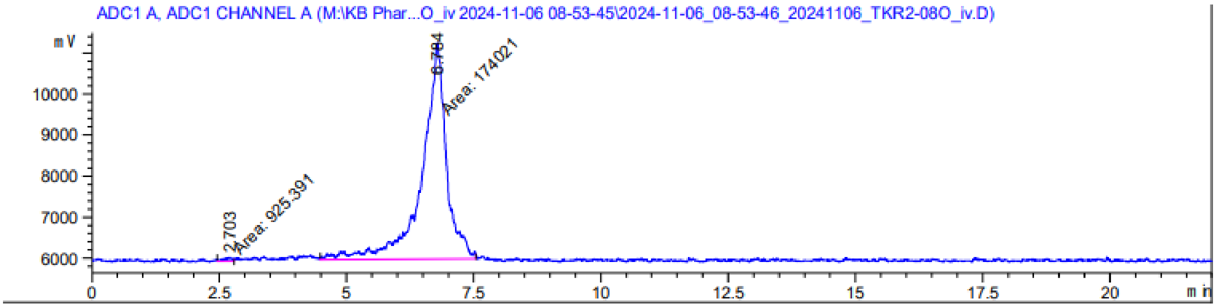

Peak #	RetTime [min]	Type	Width [min]	Area [mV*s]	Height [mV]	Area %
1	2.703	MM	0.1749	925.39056	88.19115	0.5290
2	6.784	MM	0.5513	1.74021e5	5260.71729	99.4710

[^{64}Cu]Cu-3a

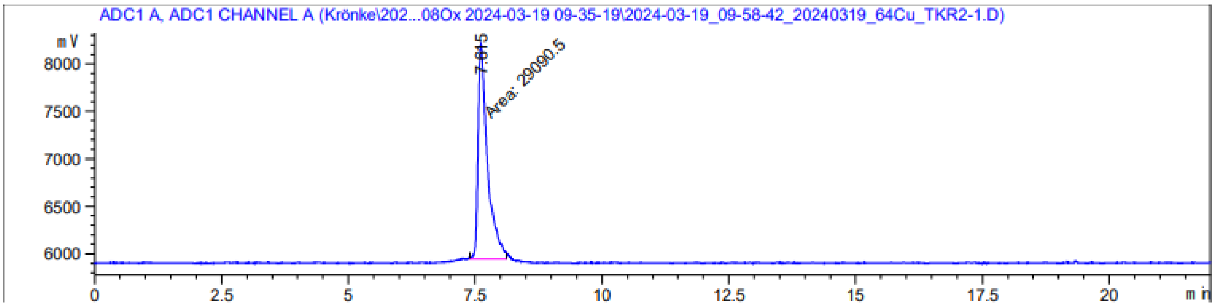

Peak #	RetTime [min]	Type	Width [min]	Area [mV*s]	Height [mV]	Area %
1	7.615	MM	0.2139	2.90905e4	2266.21460	100.0000

[^{64}Cu]Cu-4

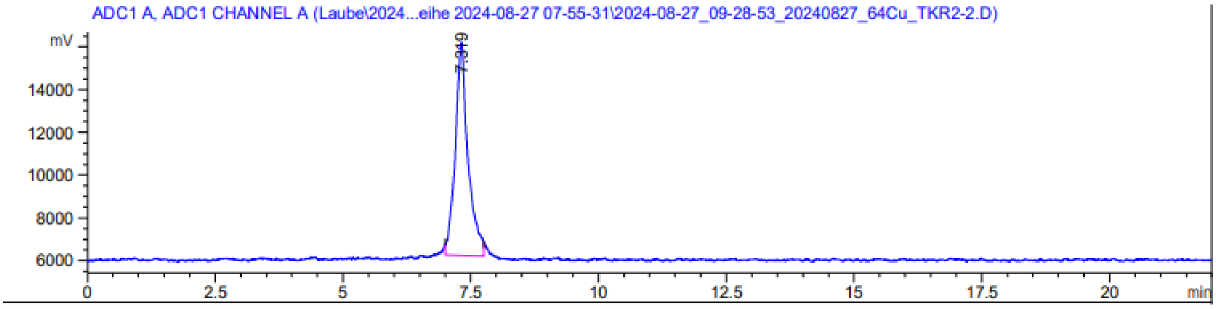

Peak #	RetTime [min]	Type	Width [min]	Area [mV*s]	Height [mV]	Area %
1	7.319	BB	0.2496	1.73824e5	9960.44629	100.0000

[^{64}Cu]Cu-5

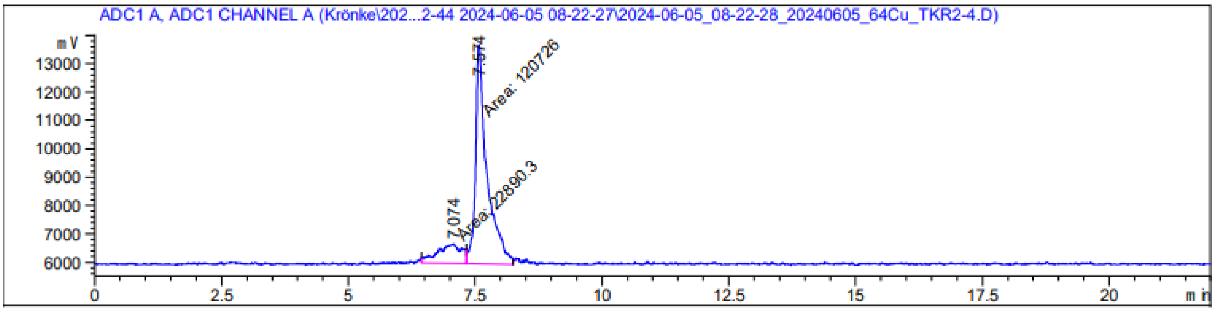

Peak #	RetTime [min]	Type	Width [min]	Area [mV*s]	Height [mV]	Area %
1	7.074	MF	0.5597	2.28903e4	681.58246	15.9385
2	7.574	FM	0.2608	1.20726e5	7716.27979	84.0615

[^{64}Cu]Cu-6

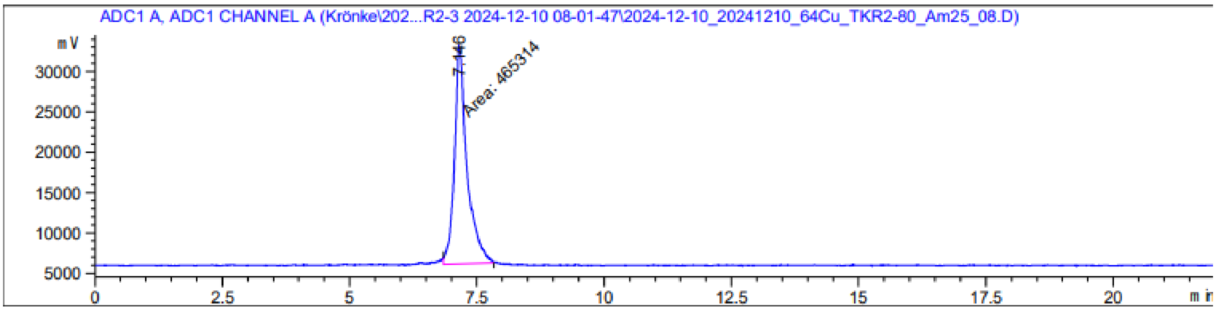

Peak #	RetTime [min]	Type	Width [min]	Area [mV*s]	Height [mV]	Area %
1	7.146	FM	0.2869	4.65314e5	2.70320e4	100.0000
Totals :				4.65314e5	2.70320e4	

[^{64}Cu]Cu-7

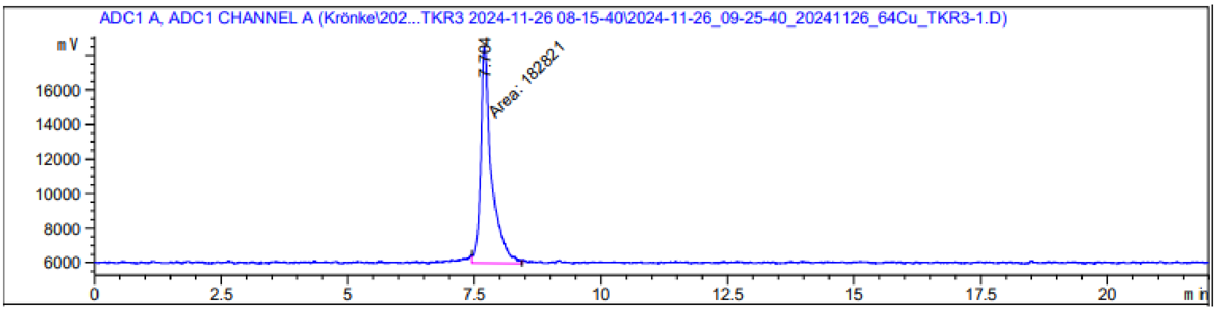

Peak #	RetTime [min]	Type	Width [min]	Area [mV*s]	Height [mV]	Area %
1	7.704	FM	0.2428	1.82821e5	1.25484e4	100.0000
Totals :				1.82821e5	1.25484e4	

[^{64}Cu]Cu-8a

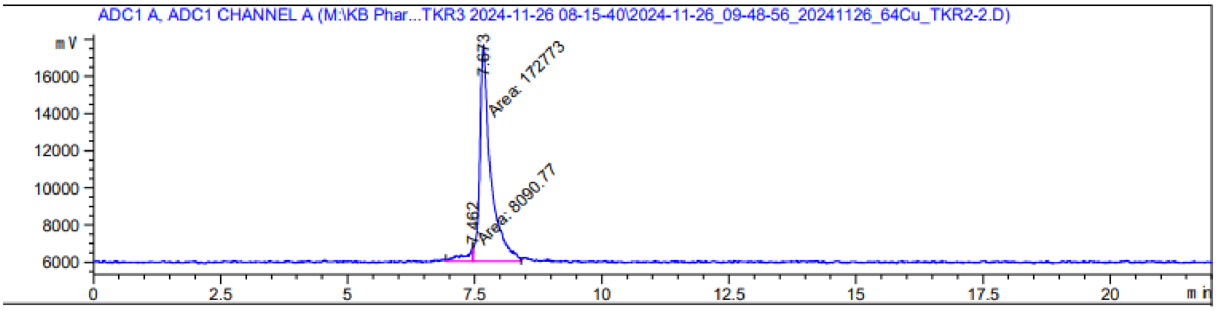

Peak #	RetTime [min]	Type	Width [min]	Area [mV*s]	Height [mV]	Area %
1	7.462	MF	0.1915	8090.77002	704.31268	4.4734
2	7.673	FM	0.2472	1.72773e5	1.16495e4	95.5266

[^{68}Ga]Ga-8c

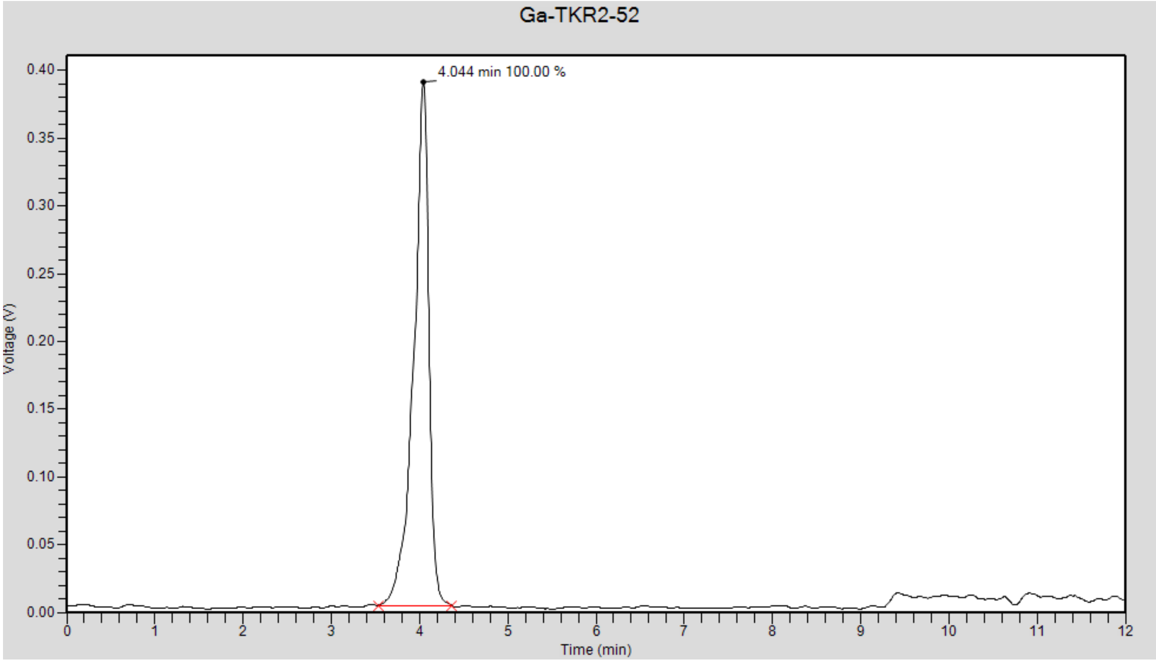

[^{68}Ga]Ga-8d

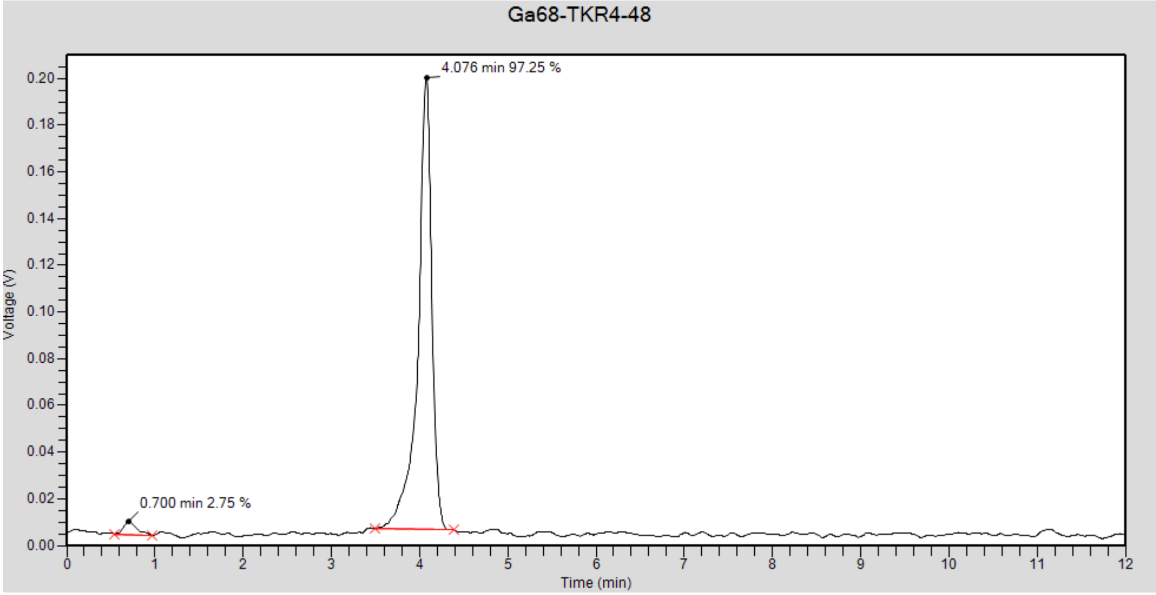

[^{64}Cu]Cu-9

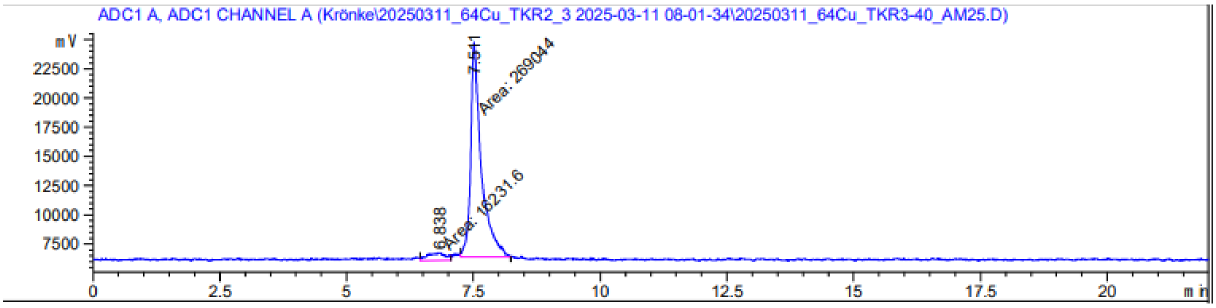

Peak #	RetTime [min]	Type	Width [min]	Area [mV*s]	Height [mV]	Area %
1	6.838	MM	0.4237	1.62316e4	638.55817	5.6898
2	7.511	MM	0.2455	2.69044e5	1.82635e4	94.3102
Totals :				2.85275e5	1.89020e4	

[^{64}Cu]Cu-10

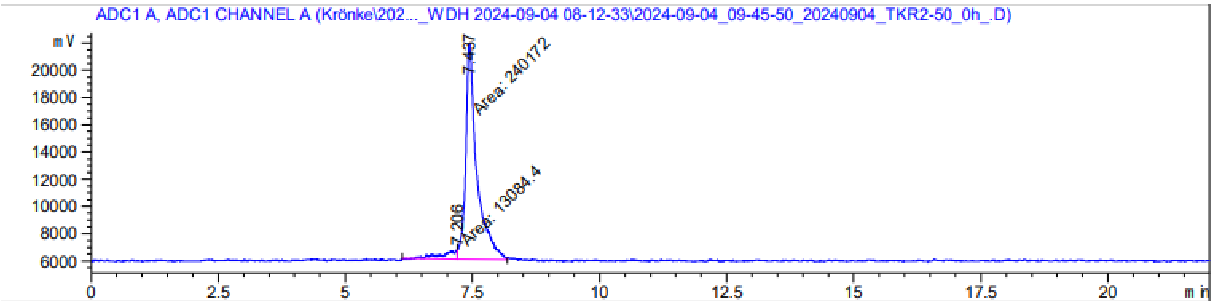

Peak #	RetTime [min]	Type	Width [min]	Area [mV*s]	Height [mV]	Area %
1	7.206	MF	0.3250	1.30844e4	671.03094	5.1664
2	7.437	FM	0.2528	2.40172e5	1.58315e4	94.8336

References

1. Brandt, F.; Ullrich, M.; Laube, M.; Kopka, K.; Bachmann, M.; Löser, R.; Pietzsch, J.; Pietzsch, H. J.; van den Hoff, J. and Wodtke, R. "Clickable" albumin binders for modulating the tumor uptake of targeted radiopharmaceuticals. *J. Med. Chem.* **2022**, *65*, 710-733.
